# The MicroRNAome of Pregnancy: Deciphering miRNA Networks at the Maternal-Fetal Interface

**DOI:** 10.1371/journal.pone.0072264

**Published:** 2013-11-22

**Authors:** Jocelyn M. Wessels, Andrew K. Edwards, Kasra Khalaj, Rami T. Kridli, Mallikarjun Bidarimath, Chandrakant Tayade

**Affiliations:** 1 Department of Biomedical Sciences, Ontario Veterinary College, University of Guelph, Guelph, Ontario, Canada; 2 Department of Biomedical and Molecular Sciences, Queen's University, Kingston, Ontario, Canada; 3 Department of Animal Production, Faculty of Agriculture, Jordan University of Science and Technology, Irbid, Jordan; University of Arkansas for Medical Sciences, United States of America

## Abstract

MicroRNAs (miRNAs) post-transcriptionally regulate a vast network of genes by inhibiting mRNA translation. Aberrant miRNA expression profiles have been implicated in pathologies and physiological processes including pregnancy and angiogenesis. Using our established model of implantation failure and spontaneous fetal loss in pigs (*Sus scrofa*), 236 miRNAs were profiled and compared between 1) non-pregnant and pregnant endometrium, 2) maternal and fetal tissues, and 3) viable and growth-arrested conceptus attachment sites by microarray and Real-Time PCR. Many significant differences in miRNA expression were observed between each of the aforementioned comparisons, and several were validated by PCR. Results indicated which miRNAs were important during pregnancy, which were elevated on the maternal or fetal side of the maternal-fetal interface, and they implicated the maternal expression of *miR-10a, 27a, 29c, 323, 331-5p, 339-3p, 374b-5p*, and *935* in the spontaneous loss observed in pigs. Several putative mRNA targets of the miRNAs (elevated in endometrium associated with arresting conceptuses) were assessed by quantitative Real-Time PCR and were depressed, supporting their regulation by miRNAs. Finally, targets were clustered by function to obtain ranked lists of gene networks that indicated which pathways/physiological processes might be important in non-pregnant (extracellular matrix factors) versus pregnant endometrium (nuclear transcription factor regulation), maternal (blood vessel development) versus fetal (neuronal differentiation) tissue, and healthy (extracellular matrix factors) versus arresting (GRAM domain) conceptus attachment sites. Overall, we demonstrate the presence of miRNAs on both sides of the maternal-fetal interface, implicate them in spontaneous fetal loss, and present a unique glimpse into the vast microRNAome of pregnancy.

## Introduction

MicroRNAs (miRNAs) are short, non-coding RNA segments that participate in the post-transcriptional regulation of gene expression. In general, they bind and destabilize or degrade their complementary mRNA, repressing gene translation [1]. To date, 1600 precursors and 2042 mature miRNAs have been reported in humans [2]. MiRNAs are promiscuous; a single miRNA has hundreds of mRNA targets [3]. As such, they have been shown to participate in a myriad of physiological processes including: embryonic and neuronal development, cellular proliferation, apoptosis, haematopoiesis, and angiogenesis [4], [5]. Altered miRNA expression profiles have been implicated in numerous disease states including: cancer [6], heart disease [7], interstitial lung disease [8], skeletal muscle disease [9], Alzheimer's disease [10], and endometriosis, a reproductive disorder [11]. While the roles of miRNAs in reproductive biology are just beginning to be unveiled, they have been shown to induce endometrial stromal cell differentiation *in vitro* [12], and participate in endometrial receptivity [13], implantation [14] and labour [15].

While many miRNA studies have been conducted in humans, the pig represents a unique model system to study the miRNA expression profiles of pregnancy. Pigs have an epitheliochorial type of placentation where there is no mixing of fetal and maternal tissues, and they have a high rate of naturally occurring spontaneous conceptus loss. During early pregnancy, around gestation day (gd) 20, up to 30% of conceptuses are spontaneously lost [16]–[18]. Previous studies found discrepancies in the number of mRNA transcripts and expression of angiogenic factors at viable conceptus attachment sites as compared to arrested littermate attachment sites [19]–[21]. Another study found 17 differentially expressed miRNAs at gd30 and 90, where the genes were responsible for cell growth, trophoblast differentiation, angiogenesis and formation/maintenance of cell junctions [22]. Thus, a more thorough understanding of the molecular differences and their regulatory cascades at the maternal-fetal interface will help decipher the fundamentals of pregnancy, and spontaneous fetal loss. Here we present a comprehensive examination of the miRNA networks, and their putative target mRNAs between: 1) non-pregnant and pregnant endometrium, 2) maternal and fetal tissues, and 3) viable and non-viable littermates.

## Materials and Methods

### Research Animals

Specific pathogen-free Yorkshire pigs from the Arkell Swine Research Station (University of Guelph, Guelph, ON, Canada) were used for this study. All animal procedures were approved by the Animal Care Committee of the University of Guelph (Animal Utilization Protocol Number 10R061). First to third parity sows were checked daily for estrus using an intact boar. At estrus, sows were placed in stalls and bred by artificial insemination using fresh pooled semen. Sows were re-bred 24 hours later. Sows (n = 3) were euthanized at gd20. Reproductive tracts were immediately collected at the University of Guelph abattoir, transported to the laboratory on ice, and examined for abnormalities. The uteri were cut longitudinally along the anti-mesometrial side, exposing all conceptuses. One healthy conceptus, and one arresting conceptus were selected per sow, based on disparity in size and vascularity as previously described [19], [20], [23], [24]. Paired samples of mesometrial endometrium and trophoblast were collected from each attachment site. Non-pregnant samples were collected from random, mesometrial endometrial sites from sows at mid-estrus (n = 4). Samples were immediately frozen and stored at −80°C.

### miRNA Extraction

Total RNA including miRNA was extracted from all samples using miRNeasy mini kits (Qiagen, Mississauga, ON, Canada) according to the manufacturer's directions. The concentration and purity of the RNA extracted was measured using the GeneQuant pro RNA/DNA calculator (Biochrom Ltd., Cambridge, UK). RNA was stored at −80°C until required.

### miRNA Microarray

Five micrograms of RNA were diluted to a volume of 50 µl with DNase/RNase free water (Gibco, Burlington, ON, Canada). A mixture of 5 µl of 3M NaOAc, pH 5.2 and 150 µl of 100% ethanol was added to each sample. The samples were immediately shipped on dry ice to LC Sciences (LC Sciences, Houston, TX, USA) for a miRNA microarray for all 236 porcine-specific miRNA sequences available in the miRBase sequence database [25], version 16 (www.mirbase.org). Briefly, small RNAs were tagged with Cy3 or Cy5, hybridized to a μParaflo microfluidic chip that contained 12 copies of all 236 porcine specific miRNA probes. After hybridization, a 16-plex microarray was performed. Fluorescent images were captured with a laser scanner and digitized. Background intensity was subtracted, signals were normalized using a LOWESS filter (locally-weighted regression), and each chip contained multiple control probes for quality control and assurance, and to allow for inter-chip comparisons. The average signal intensity for each miRNA was calculated. Biological replicates were grouped, and averaged. Fold change was calculated by dividing averaged signal intensities for each miRNA. Student's t-tests were performed for all comparisons: non-pregnant endometrium (NP) vs. endometrium associated with healthy conceptus attachment sites (HE), NP vs. endometrium associated with arresting conceptus attachment sites (AE), HE vs. trophoblast associated with healthy conceptus attachment site (HT), AE vs. trophoblast associated with arresting conceptus attachment sites (AT), HE vs. AE, HT vs. AT, and differentially detected signals were those with a *p* value<0.05. The microarray data in this paper has been published in the NCBI Gene Expression Omnibus [26] and can be accessed at (http://www.ncbi.nlm.nih.gov/geo/query/acc.cgi?acc=GSE45761). (GEO Accession Number: GSE45761).

### miRNA Target Prediction

To understand the biological significance of the differentially expressed miRNAs, the miRNA-target prediction resource miRecords [27] (http://mirecords.biolead.org/) was used to create a list of putative mRNA targets for the miRNAs found to differ between tissues with a *p* value<0.05. miRecords integrated results from 11 target prediction programs (DIANA-microT, MicroInspector, miRanda, miRDB/miRTarget2, miTarget, NBMirTar, PicTar, PITA, RNA22, RNAhybrid, and TargetScan/TargetScanS), each with their own unique target prediction algorithm.

Although porcine-specific miRNA microarrays were commercially available, miRecords did not have the capacity to search for porcine miRNA targets. Identical miRBase gene names across species indicate orthologs, and here we have assumed sequence conservation between humans (hsa-miR) and pigs (ssc-miR) [28], [29]. Additionally, in a comparison of miRNA binding sites in the 3′ UTR it was reported that the majority of these sites were preferentially conserved across species [29]. Therefore, predictions were based on the miRNA/mRNA interactions in humans, not pigs. The ‘Validated Targets’ and ‘Predicted Targets’ components of miRecords were used to search for putative targets of each differentially expressed miRNA. Only targets predicted by 6 of the 11 (55%) prediction programs were included in the list of putative targets. For *miR-331-5p*, *339-3p*, and *935* these parameters were loosened to targets predicted by 4 of 11 programs. This generated a large database containing both validated and predicted miRNA targets for each miRNA which was differentially expressed between pig reproductive tissues (NP vs. HE; HE vs. HT; AE vs. AT; and HE vs. AE) (Material S2: Putative miRNA Targets).

### Gene Functional Classification and Annotation

Each list of miRNA target genes was run through a high-throughput data mining software. The Database for Annotation, Visualization, and Integrated Discovery (DAVID) software (http://david.abcc.ncifcrf.gov/) [30] was used to analyze these lists in an attempt to derive biological meaning from them. First, gene functional classifications were performed to classify each list of genes into groups, and rank those groups according to the number of times functionally related genes were represented within the list (Material S4: Functional Classifications of Target mRNAs). Second, functional annotation charts were created to determine the magnitude of enrichment of the genes in the submitted lists compared to the human genome as a background (Material S5: Functional Annotation Charts of Target mRNAs). Finally, DAVID functional annotation clustering was used to group genes within the list based on their co-associations and function (Material S3: Functional Annotation Clusters of Target mRNAs).

### Selection of miRNAs and Putative mRNA Targets for Validation by Real-Time PCR

Of the 236 miRNAs probed in the microarray, 12 (*miR-10a*, *27a*, *29a*, *29c*, *30b-5p*, *99a*, *148a*, *148b*, *323*, *331-5p*, *339-3p*, and *374b-5p*) were selected for secondary validation by quantitative Real-Time PCR. The 12 miRNAs chosen were observed to be significantly different (p<0.05) by microarray in various early gestational porcine tissues.

Of the thousands of putative mRNA targets identified by the target prediction software, 11 were selected (5 validated, and 6 predicted; *AhR, CCNG1*, *CDC42*, *DNMT3A*, *FADD*, *FOXO1*, *GPR37*, *IFI30*, *MMD*, *USF2*, and *USP46*) for secondary validation by quantitative Real-Time PCR. These 11 mRNA targets were chosen from the list mRNA targets of the miRNAs which were significantly elevated in arresting endometrium as compared to healthy endometrium.

### cDNA Preparation

Total RNA including miRNA from endometrial and trophoblast samples was reverse transcribed using the miScript Reverse Transcription kit (Qiagen, Mississauga, ON, Canada) as per the manufacturer's protocol. Additional cDNA from endometrium associated with healthy and arresting attachment sites was available for the Real-Time PCR experiment where mRNA targets were quantified. For this experiment, healthy (n = 6) and arresting (n = 6) gd20 endometrium was used for relative quantification.

### Validation of miRNA Microarray by Real-Time PCR

Quantitative Real-Time PCR was performed to validate microarray results using the miScript PCR System (Qiagen, Mississauga, ON, Canada). The expression level of the 12 miRNAs selected were measured using Poly-T miScript Universal Primers and porcine-specific custom-designed primers (Qiagen, Mississauga, ON, Canada). Real-Time PCR was performed using the plate-based LightCycler 480 PCR System (Roche Diagnostics, Laval, QC, Canada). All samples were run in quadruplicate for each of the 12 miRNA genes and in duplicate for the control gene RNU1A (denaturation: 95°C, 15 min; amplification: 45 cycles: 95°C for 15 s, 55°C for 30 s, 70°C for 30 s; melting curve: 70–95°C at a rate of 0.1°C per second). RNU1A was selected as a miRNA reference gene because it was previously demonstrated by our lab to be stably expressed during early porcine pregnancy [31]. Relative quantification was performed using the 2^−ΔCp^ method [32]. All miRNA PCR products were run on a 1% agarose gel to estimate their size. Specific information about the miRNAs (PCR efficiencies, melting temperatures, product sizes, GenBank accessions, etc.) can be found in Table 1.

**Table 1:** miRNAs Assessed by Real-Time PCR.

miRNA	Efficiency	Melting Temp. (°C)	Confirmed by Sequencing	Size (bp)	GenBank Accession
*ssc-miR-10a*	1.804	75.16	Yes	22	JX185556
*ssc-miR-27a*	1.655	75.91	No	∼20	N/A
*ssc-miR-29a*	1.609	75.25	Yes	∼20	JX185557
*ssc-miR-29c*	1.720	74.94	No	∼20	N/A
*ssc-miR-30b-5p*	1.680	75.29	Yes	21	JX185558
*ssc-miR-99a*	1.606	75.27	Yes	18	JX185559
*ssc-miR-148a*	1.476	75.14	No	∼20	N/A
*ssc-miR-148b*	1.713	75.26	Yes	20	JX185560
*ssc-miR-323*	1.743	76.92	Yes	20	JX185552
*ssc-miR-331-5p*	1.663	75.99	Yes	20	JX185553
*ssc-miR-339-3p*	1.689	76.58	Yes	17	JX185554
*ssc-miR-374b-5p*	1.537	75.10	Yes	21	JX185562
*RNU1A*	1.762	78.49	Yes	150	JN617883

### Assessment of miRNA Targets by Real-Time PCR

Transcripts for 11 mRNA targets of the miRNAs were relatively quantified by Real-Time PCR, in duplicate using the LightCycler 480 software (Roche Diagnostics, Laval, QC, Canada). Primers were designed using Primer3 software (http://frodo.wi.mit.edu/primer3/) from pig sequences available on NCBI's Nucleotide, and tested for hairpins, self-dimers, and hetero-dimers using OligoAnalyzer 3.1 (http://www.idtdna.com/analyzer/applications/oligoanalyzer/). Primer sequences are listed in Table 2. Relative quantification was performed using GAPDH as a control gene. GAPDH did not differ across groups by one-way ANOVA.

**Table 2:** mRNAs Assessed by Real-Time PCR.

Gene Name	Primer	Efficiency	Melting Temp. (°C)	Size (bp)	GenBank Accession
***AhR***	For: GCAGTCAAATGCACGCTTAG	2.057	80.32	276	KC012627
	Rev: GAGCTAGGGTTGAGGGAATC				
***CCNG1***	For: AGGTCTGCGGCTTGAGACTA	2.048	80.83	272	KC012621
	Rev: ATCAGTTGCCAGTGGGACAT				
***CDC42***	For: TGATTGGTGGAGAGCCATATAC	1.863	80.64	295	KC012622
	Rev: TCAGCAGTCTCTGGAGTGATAG				
***DNMT3A***	For: ACAACGACGAGAGCGACACT	1.765	86.42	294	KC012620
	Rev: ACTTCTGCCGCACCTCATAC				
***FADD***	For: CGCCATCGAGGAGAAGTATC	1.817	90.61	271	KC012625
	Rev: AAGAGCAGCGGGTCATCAG				
***FOXO1***	For: TACATTTCGCCCACGGACTA	1.908	83.78	262	KC012619
	Rev: GATGGTGCCTGGTGAAGACT				
***GPR37***	For: AGCCGAAATACCACCAGAGT	1.801	86.00	207	KC012626
	Rev: CAGCCAAACTTGCTGTCATA				
***IFI30***	For: GCAGGAGTGCAAGATGAACA	1.796	82.79	250	KC012618
	Rev: ATATTCATGGGGTGGCTTCA				
***MMD***	For: GCTTTCCCATTAGCCGTGTA	1.950	77.77	256	KC012628
	Rev: GCAATTTCTCCATGCTTCAC				
***USF2***	For: CCAGTTCCGCACAGAGAATA	1.864	77.18	263	KC012623
	Rev: CACCATTGCTGAAGGGATTT				
***USP46***	For: CAGGATGCTCACGAGTTTCTAA	1.808	80.32	282	KC012624
	Rev: GAGACAGTGGGTGATGGATGTA				
***GAPDH***	For: GCGTGAACCATGAGAAGTATG	1.880	88.09	276	Designed from: NM_001206359.1
	Rev: GTCAGATCCACAACCGACAC				

### Cloning and Sequencing

Fresh miRNA PCR products were cloned into a plasmid vector with the topoisomerase-TA cloning kit (Life Technologies, Burlington, ON, Canada), purified using the Genelute Plasmid Mini-Prep Kit (Sigma, St. Louis, MO, USA), and sent for sequencing (Laboratory Services, University of Guelph, Guelph, ON, Canada). PCR product for mRNAs were directly sent for sequencing. Each resulting sequence underwent BLASTN analysis on the National Center for Biotechnology Information website. Sequences were submitted to NCBI GenBank (Table 1). Three of the 12 miRNAs (*miR-27a*, *29c*, and *148a*) selected for Real-Time PCR validation of microarray results could not be positively identified by sequencing, even after cloning was repeated two additional times. However, the primers were purchased commercially and the product sizes were as expected. Sequences for mRNA studies were directly sequenced after PCR, and submitted to GenBank (Table 2).

### Statistical Analyses

Statistical analysis of microarray data was performed by LC Sciences (LC Sciences, Houston, TX, USA). Briefly, measurement of each miRNA in the microarray was repeated 12 times and the signal intensities were averaged. Groups were compared by t-test, and a *p* value of <0.05 was considered significant.

Real-Time PCR data were also compared by t-test (SigmaPlot 10.0 Systat Software Inc., Chicago, IL, USA) to match comparisons made for the microarray data. A *p* value of <0.05 between groups was considered significant. Bars on the graphs represent the mean plus the standard error of measurement (SEM).

For both the microarray and Real-Time PCR experiments the only statistical comparisons made were between NP vs. HE, NP vs. AE, HE vs. HT, AE vs. AT, HE vs. AE, and HT vs. AT. Other comparisons not made (eg. NP vs. AT) were deemed to be biologically irrelevant.

### NCBI GenBank Accession Numbers

*ssc-miR-10a* (JX185556); *ssc-miR-29a* (JX185557); *ssc-miR-30b-5p* (JX185558); *ssc-miR-99a* (JX185559); *ssc-miR-148b* (JX185560); *ssc-miR-323* (JX185552); *ssc-miR-331-5p* (JX185553); *ssc-miR-339-3p* (JX185554); *ssc-miR-374b-5p* (JX185562); *RNU1A* (JN617883); *AhR* (KC012627), *CCNG1* (KC012621); *CDC42* (KC012622); *DNMT3A* (KC012620); *FADD* (KC012625); *FOXO1* (KC012619); *GPR37* (KC012626); *IFI30* (KC012618); *MMD* (KC012628); *USF2* (KC012623); *USP46* (KC012624).

**NCBI Gene Expression Omnibus Number:** GSE45761

## Results

### miRNA Expression by Microarray during Porcine Pregnancy

**Figure 1:**
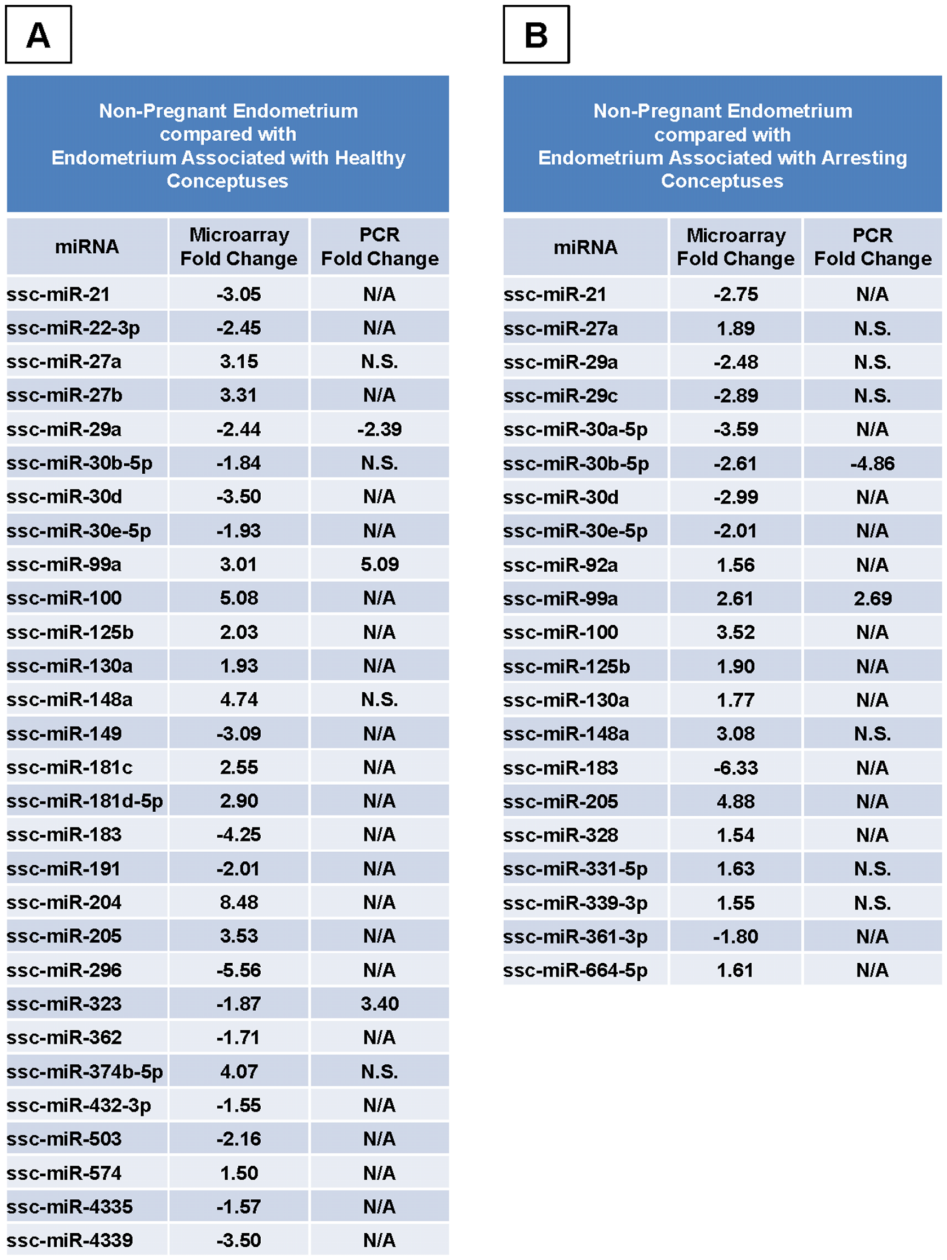
miRNA Expression during Porcine Pregnancy. Expression levels of miRNAs detected by microarray which were significantly different (*p*<0.05) between non-pregnant endometrium (n = 4) as compared to endometrium associated with healthy conceptus attachment sites (A, n = 3), and arresting conceptus attachment sites (B, n = 3). Real-Time PCR validation was performed for several miRNAs, and significant fold changes are listed for comparison. A positive fold change indicates that the miRNA was elevated in non-pregnant endometrium. A negative fold change indicates a decrease in the miRNA in the non-pregnant endometrium. N/A: not assessed, N.S.: not significant.

In total 29 miRNAs differed significantly (p<0.05) between non-pregnant endometrium, and gd20 endometrium isolated from healthy porcine attachment sites (Figure 1A and Material S1: Microarray Heat Maps). In non-pregnant endometrium, 13 miRNAs (*miR-27a, 27b, 99a, 100, 125b, 130a, 148a, 181c, 181d-5p, 204, 205, 374b-5p*, and *574*) were elevated, and 16 miRNAs (*miR-21, 22-3p, 29a, 30b-5p, 30d, 30e-5p, 149, 183, 191, 296, 323, 362, 432-3p, 503, 4335*, and *4339*) were decreased when compared to healthy gd20 endometrium.

When non-pregnant endometrium was compare to gd20 endometrium isolated from arresting porcine attachment sites 21 miRNAs differed significantly (p<0.05) (Figure 1B). In non-pregnant endometrium, 12 miRNAs (*miR-27a, 92a, 99a, 100, 125b, 130a, 148a, 205, 328, 331-5p, 339-3p*, and *664-5p*) were elevated, and 9 miRNAs (*miR-21, 29a, 29c, 30a-5p, 30b-5p, 30d, 30e-5p, 183*, and *361-3p*) were decreased when compared to arresting gd20 endometrium.

### miRNA Expression by Microarray in Endometrium and Trophoblast

**Figure 2:**
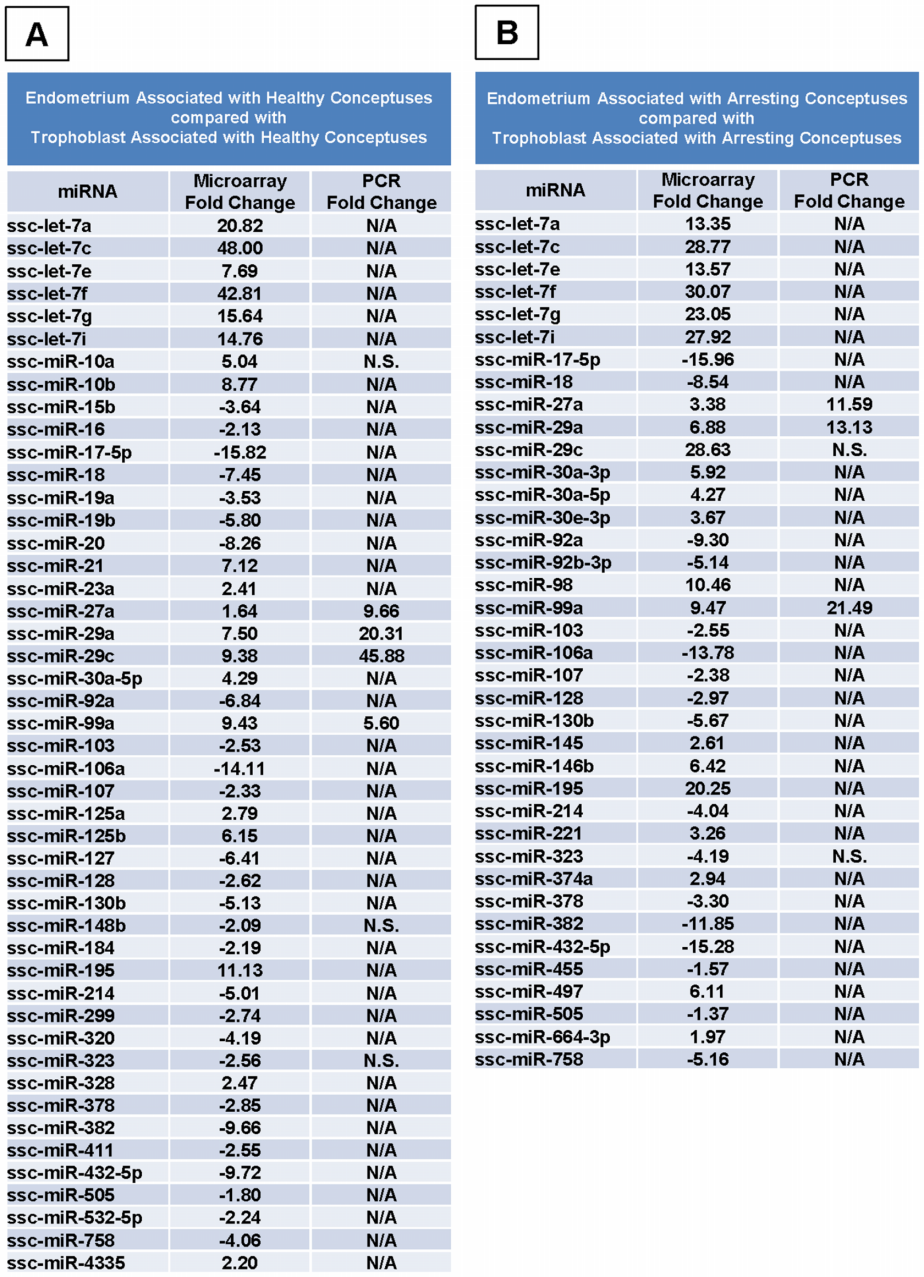
miRNA Expression in Endometrium and Trophoblast. Expression levels of miRNAs detected by microarray which were significantly different (*p*<0.05) between endometrium (n = 3) and trophoblast (n = 3) from isolated from healthy (A) and arresting conceptus attachment sites (B). Real-Time PCR validation was performed for several miRNAs, and significant fold changes are listed for comparison. A positive fold change indicates that the miRNA was elevated in endometrium. A negative fold change indicates a decrease in the miRNA in endometrium. N/A: not assessed, N.S.: not significant.

In total 47 miRNAs differed significantly (p<0.05) between gd20 endometrium, and trophoblast isolated from healthy porcine attachment sites (Figure 2A). In healthy gd20 endometrium, 20 miRNAs (*let-7a, let-7c, let-7e, let-7f, let-7g, let-7i, miR-10a, 10b, 21, 23a, 27a, 29a, 29c, 30a-5p, 99a, 125a, 125b, 195, 328*, and *4335*) were elevated and 27 miRNAs (*miR-15b, 16, 17-5p, 18, 19a, 19b, 20, 92a, 103, 106a, 107, 127, 128, 130b, 148b, 184, 214, 299, 320, 323, 378, 382, 411, 423-5p, 505, 532-5p*, and *758*) were decreased when compared to healthy gd20 trophoblast (Material S1: Microarray Heat Maps).

In total 38 miRNAs differed significantly (p<0.05) between gd20 endometrium, and trophoblast isolated from arresting porcine attachment sites (Figure 2B). In arresting gd20 endometrium, 21 miRNAs (*let-7a, let-7c, let-7e, let-7f, let-7g, let-7i, miR-27a, 29a, 29c, 30a-3p, 30a-5p, 30e-3p, 98, 99a, 145, 146b, 195, 221, 374a, 497*, and *664-3p*) were elevated and 17 miRNAs (*miR-17-5p, 18, 92a, 92b-3p, 103, 106a, 107, 128, 130b, 214, 323, 378, 382, 432-5p, 455, 505*, and *758*) were decreased when compared to arresting gd20 trophoblast.

### miRNA Expression by Microarray in Healthy and Arresting Tissues

**Figure 3:**
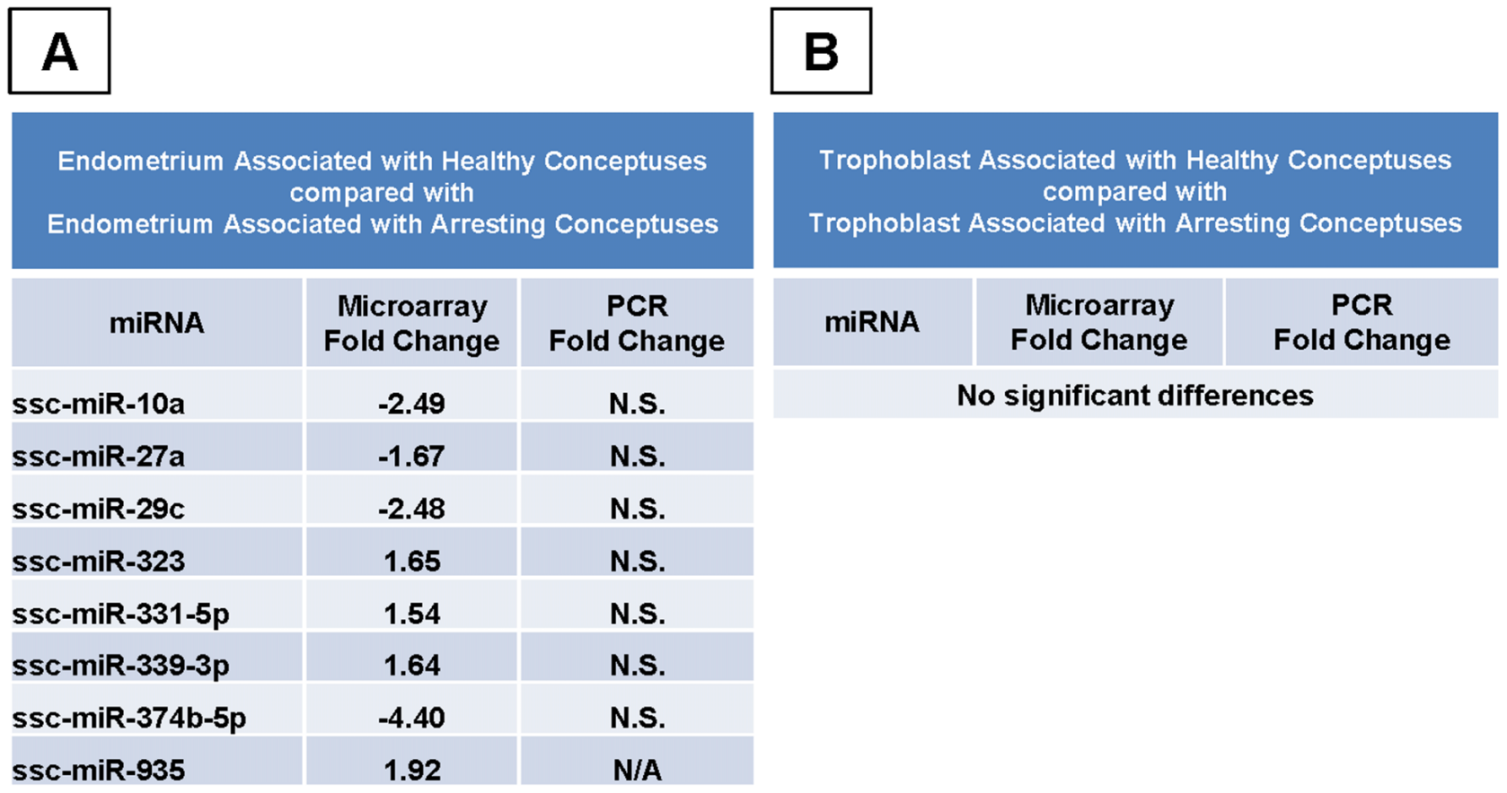
miRNA Expression in Healthy and Arresting Tissues. Expression levels of miRNAs detected by microarray which were significantly different (*p*<0.05) between endometrium from healthy conceptus attachment sites (n = 3) compared to endometrium from arresting attachment sites (n = 3) (A). No miRNAs differed significantly between trophoblast from healthy (n = 3) versus trophoblast from arresting attachment sites (n = 3) (B). Real-Time PCR validation was performed for several miRNAs, and significant fold changes are listed for comparison. A positive fold change indicates that the miRNA was elevated in the endometrium from healthy attachment sites. A negative fold change indicates a decrease in the miRNA in the endometrium from healthy attachment sites as compared to the endometrium from arresting sites. N/A: not assessed, N.S.: not significant.

In total 8 miRNAs differed significantly (p<0.05) between healthy gd20 endometrium, and arresting gd20 endometrium (Figure 3A). In healthy gd20 endometrium, 4 miRNAs (*miR-323, 331-5p, 339-3p*, and *935*) were elevated and 4 miRNAs (*miR-10a, 27a, 29c*, and 3*74b-5p*) were decreased when compared to arresting gd20 endometrium. (Material S1: Microarray Heat Maps).

No significant differences were found between miRNAs isolated from healthy compared to arresting trophoblast (Figure 3B). Healthy trophoblast expressed 1 miRNA, *miR-148b*, which approached significance (p<0.10), at a higher level than arresting trophoblast.

### Validation of miRNA by Real-Time PCR

**Figure 4:**
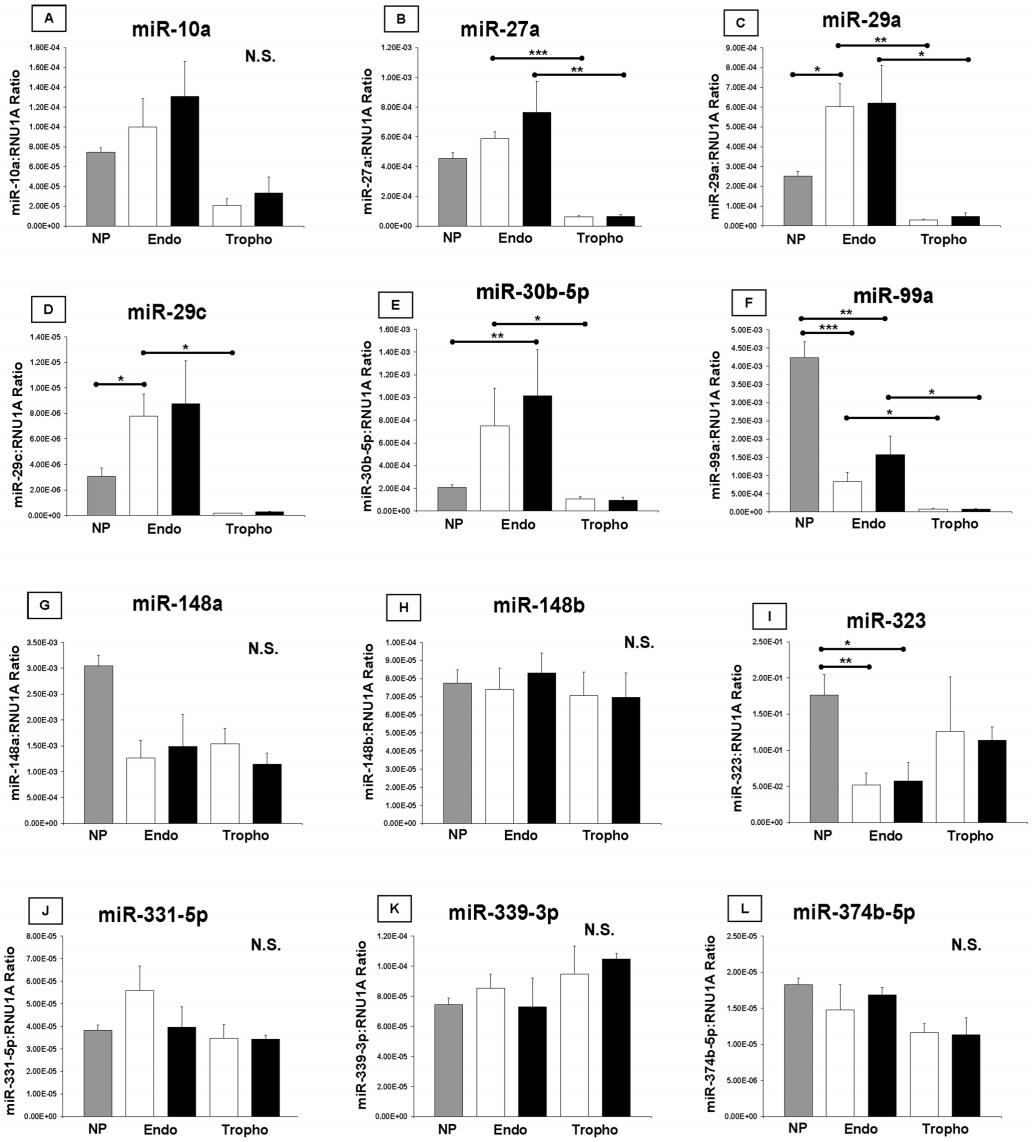
Validation of microarray results by Real-Time PCR. Twelve miRNAs were selected for Real-Time PCR validation of significant differences seen by microarray. Relative quantification against the control gene RNU1A was performed for all miRNAs. For each gene, non-pregnant endometrium (NP, n = 4) vs. endometrium associated with healthy conceptus attachment sites (HE, n = 3), NP vs. endometrium associated with arresting conceptus attachment sites (AE n = 3), HE vs. trophoblast associated with healthy conceptus attachment sites (HT, n = 3), AE vs. trophoblast associated with arresting conceptus attachment sites (AT, n = 3), HE vs. AE, and HT vs. AT were compared by t-test to mirror the statistical comparisons made with the microarray data. Bars on the graphs represent the mean plus the SEM. Grey bars indicate non-pregnant endometrium. White bars indicate tissues from healthy conceptus attachment sites. Black bars indicate tissues from arresting conceptus attachment sites. Statistical analyses revealed the power of the t-tests to be low, and this may have muted some differences between the tissues. Endo: endometrium, NP: non-pregnant endometrium, N.S.: not significant, Tropho: trophoblast.

The expression levels of 12 miRNAs (*miR-10a*, *27a*, *29a*, *29c*, *30b-5p*, *99a*, *148a*, *148b*, *323*, *331-5p*, *339-3p*, and *374b-5p*) were measured in all samples by Real-Time PCR to validate microarray results (Figure 4). Three of the 12 miRNAs (*miR-27a*, *29c*, and *148a*) could not be positively identified by sequencing. The primers were purchased commercially, gave single melting peaks of comparable temperatures to the other miRNAs tested, and were the approximate product size expected, indicating that the primer pairs were isolating the correct products.

### miRNA Transcripts by Real-Time PCR during Porcine Pregnancy

Of the 29 significant differences observed by microarray in miRNA transcripts compared between non-pregnant endometrium and healthy endometrium, 7 miRNAs (*miR-27a*, *29a*, *30b-5p*, *99a*, 1*48a*, *323*, and *374b-5p*) were assessed by Real-Time PCR. Of these, 2 (*miR-29a*, *99a*) followed the differences seen by microarray in direction and magnitude (p<0.05), 1 (*miR-323*) opposed results seen by microarray (p<0.05), and no significant differences were observed for the remaining miRNAs. Array and PCR results are compared in Figure 1A.

Of the 21 significant differences observed by microarray in miRNA transcripts compared between non-pregnant endometrium and arresting endometrium, 8 miRNAs (*miR-27a*, *29a*, *29c*, *30b-5p*, *99a*, *148a*, *331-5p*, and *339-3p*) were assessed by Real-Time PCR. Of these, 2 (*miR-30b-5p*, *99a*) were found to mirror microarray results in direction and magnitude (p<0.05). The remainder were not statistically different between these tissues. Array and PCR results are compared in Figure 1B.

### miRNA Transcripts by Real-Time PCR between Endometrium and Trophoblast

Of the 47 significant differences observed by microarray in miRNA transcripts between healthy endometrium and healthy trophoblast, 7 miRNAs (*miR-10a*, *27a*, *29a*, *29c*, *99a*, *148b*, and *323*) were assessed by Real-Time PCR. Of these, 4 (*miR-27a*, *29a*, *29c*, *99a*) were confirmed to be significantly elevated in healthy endometrium than healthy trophoblast. Microarray and PCR results are compared in Figure 2A.

Of the 38 significant differences observed by microarray in miRNA transcripts between arresting endometrium and arresting trophoblast, 5 (*miR-27a*, *29a*, *29c*, *99a*, and *323*) were assessed by Real-Time PCR. Of these 5 genes, 3 (*miR-27a*, *29a*, *99a*) followed the microarray results in direction, but were greater in magnitude (p<0.05). *miR-29c* and *323* did not differ statistically. Microarray and PCR results are compared in Figure 2B.

### miRNA Transcripts by Real-Time PCR between Healthy and Arresting Tissues

Of the 8 significant differences observed by microarray between healthy endometrium and arresting endometrium, 7 miRNAs (*miR-10a*, *27a*, *29c*, *323*, *331-5p*, *339-3p*, and *374b-5p*) were assessed by Real-Time PCR but the results could not be validated. Microarray and PCR results are compared in Figure 3A.

While no significant differences were observed by microarray between healthy and arresting trophoblast, *miR-148b* verged on significance (p<0.10), and was assessed by Real-Time PCR but the results did not significantly differ.

### Assessment of Putative miRNA Target Genes by Real-Time PCR

Several lists of putative mRNA targets for the miRNAs which differed between tissues were generated using stringent prediction parameters as described in Methods (Material S2: Putative miRNA Targets).

**Figure 5:**
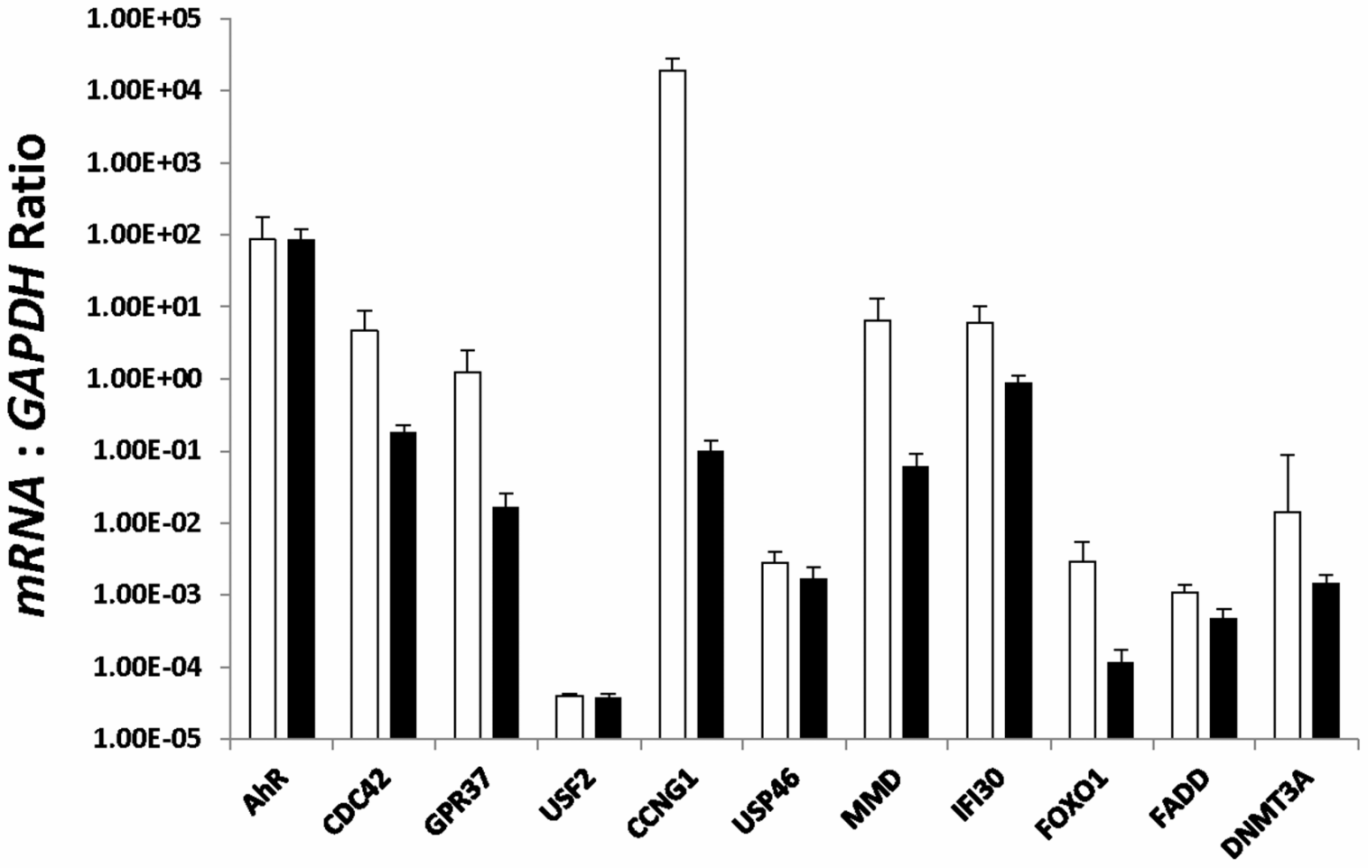
Assessment of Putative miRNA Target Genes by Real-Time PCR. Eleven mRNA targets of the miRNAs that were significantly elevated in arresting as compared to healthy endometrium in the microarray experiment were selected for quantification by Real-Time PCR. Each mRNA was relatively quantified against the control gene GAPDH. As miRNAs negatively regulate their mRNA targets, a decrease in mRNA transcripts in endometrium from arresting conceptus attachment sites was expected. Endometrium from healthy attachment sites (white bars, n = 6) was compared to endometrium from arresting sites (black bars, n = 6) by t-test. Bars on the graphs represent the mean plus the SEM. Data is presented on a logarithmic scale to display all genes on one graph. No statistically significant differences were observed, however each of the 11 mRNAs quantified was decreased in arresting endometrium. Statistical analyses revealed the power of the t-tests to be low, and this may have muted differences between the tissues.

The expression level of 11 putative target genes (5 validated, and 6 predicted; *AhR*, *CCNG1*, *CDC42*, *DNMT3A*, *FADD*, *FOXO1*, *GPR37*, *IFI30*, *MMD*, *USF2*, and *USP46*) of the miRNAs which were demonstrated to be significantly elevated in arresting endometrium compared to healthy endometrium by microarray were measured by Real-Time PCR (Figure 5). A decrease in each mRNA was expected in arresting endometrium compared to healthy endometrium as miRNAs post-transcriptionally regulate their targets by binding and degrading them. While no significant differences in transcript numbers were observed between the tissues, 9 of 11 genes assessed followed the expected trend of lowered transcript numbers in arresting endometrium.

### Functional Clustering of Putative Target mRNAs

**Figure 6:**
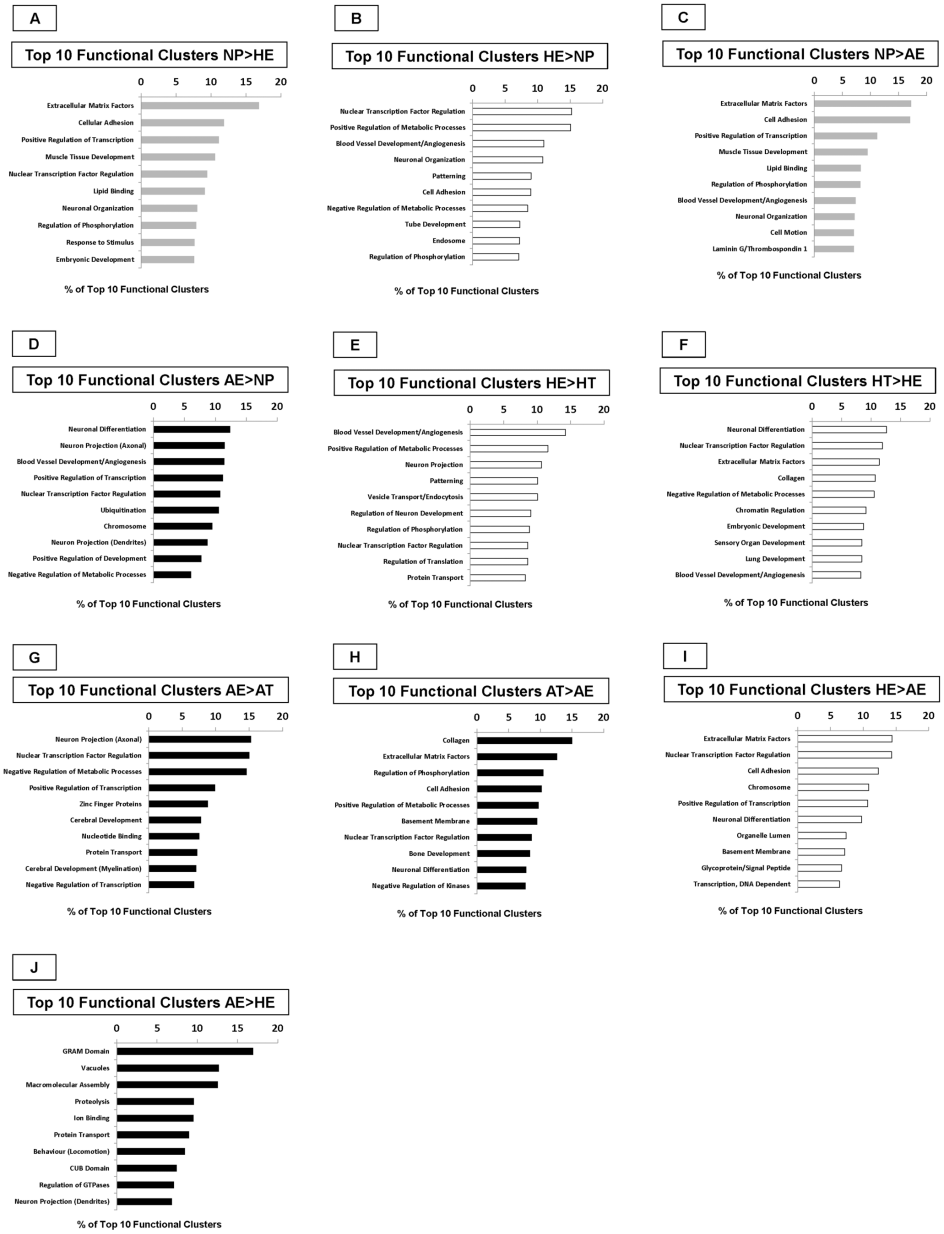
Functional Clustering of mRNA Target Genes. DAVID software was used to create groupings by gene function for the putative mRNA targets. The top ten clusters for each tissue comparison are listed as a percentage of the top ten by enrichment score. The list represents the gene families which may be increased in one tissue over the other because of the differential expression of miRNAs which was seen by microarray. A comparison of the top ten clusters in (A) non-pregnant endometrium (NP) and (B) endometrium from healthy attachment sites (HE), (C) NP and endometrium from arresting attachment (D) sites (AE), (E) HE and trophoblast (F) from healthy conceptus attachment sites (HT), (G) AE and trophoblast (H) from arresting conceptus attachment sites (AT), and (I) HE and (J) AE. There were no significant differences in miRNAs found between HT and AT, thus there are no mRNA target gene clusters for these tissues. Grey bars indicate non-pregnant endometrium. White bars indicate tissues from healthy attachment sites. Black bars indicate tissues from arresting attachment sites. AE: arresting endometrium, AT: arresting trophoblast, HE: healthy endometrium, HT: healthy trophoblast, NP: non-pregnant endometrium.

**Figure 7:**
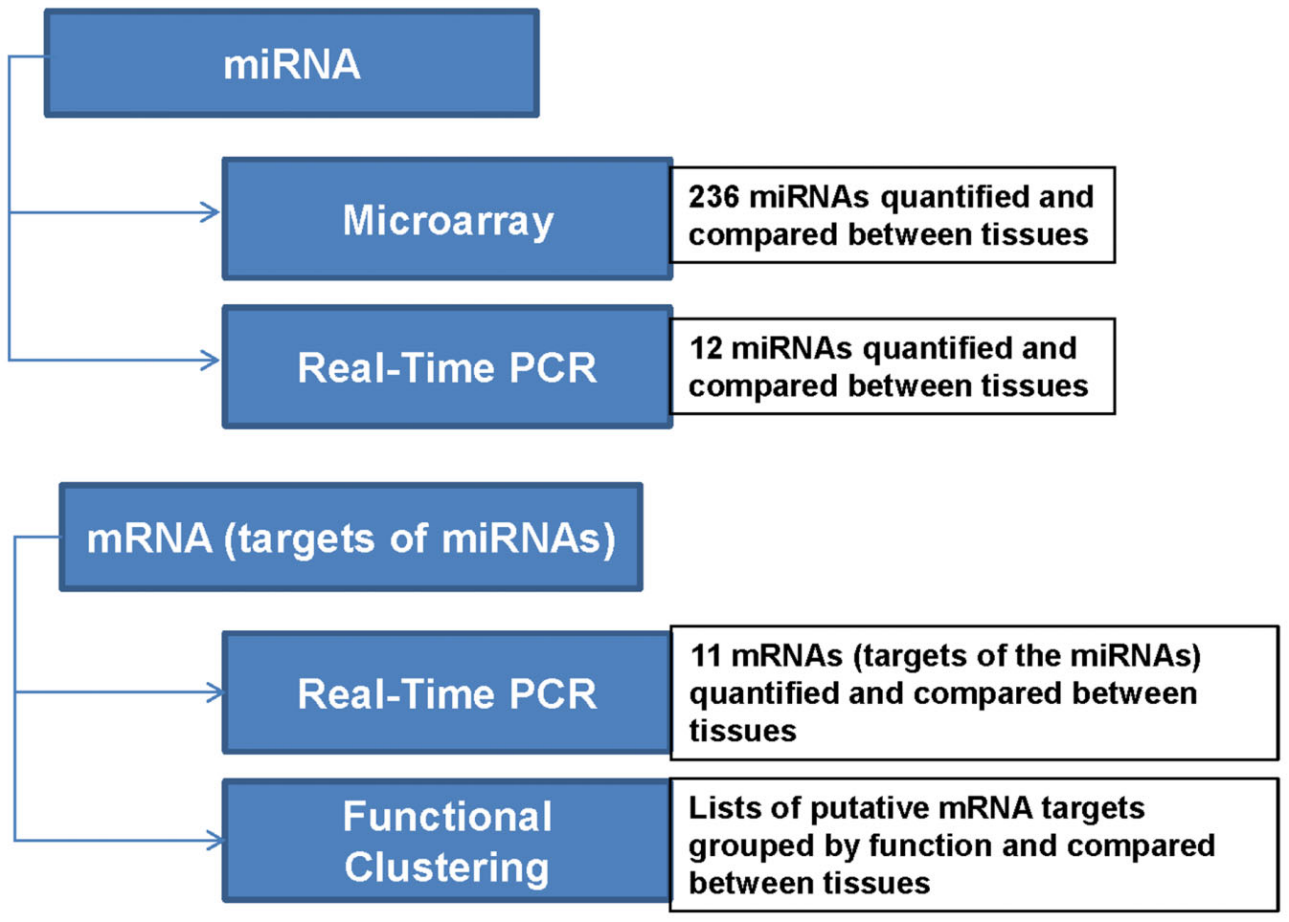
Flowchart outlining Experimental Methodology. First, 236 pig-specific miRNAs were assessed by microarray and comparisons between gestation day 20 tissues were made. Second, Real-Time PCR was used to validate several of the significant differences seen by microarray. Third, select mRNA targets of the miRNAs were assessed by Real-Time PCR. Finally, all putative targets were clustered by function to infer which cellular processes and pathways would be affected by the differential expression of miRNAs between gestational tissues. For all experiments the statistical comparisons made were non-pregnant endometrium (NP) vs. endometrium associated with healthy conceptus attachment sites (HE), NP vs. endometrium associated with arresting conceptus attachment sites (AE), HE vs. trophoblast associated with healthy conceptus attachment sites (HT), AE vs. trophoblast associated with arresting conceptus attachment sites (AT), HE vs. AE, and HT vs. AT.

Functional clustering was used to group genes within the list based on their co-associations and function. The complete list is found in Material S3: Functional Annotation Clusters of Target mRNAs. The top ten functional clusters which would be affected by the differences in miRNAs as observed by microarray are summarized in Figure 6. The bars indicate the proportion of genes in the top ten list related to that cluster. Extracellular matrix factors were at the top of the list of genes which would be increased in non-pregnant endometrium when compared to pregnant endometrium, regardless of whether the endometrium was associated with a healthy (Figure 6A) or arresting conceptus (Figure 6C). Nuclear transcription factor regulation topped the list of genes when endometrium from healthy conceptus attachment sites was compared to non-pregnant endometrium (Figure 6B), whereas neuronal differentiation was the top cluster when endometrium associated with arresting conceptuses was compared to non-pregnant endometrium (Figure 6D). In endometrium from healthy conceptus attachment sites, blood vessel development/angiogenesis was the top functional cluster when compared to trophoblast from the same attachment site (Figure 6E). Neuronal differentiation was found to be the top cluster represented in the trophoblast from healthy conceptus attachment sites when compared to the endometrium from these sites (Figure 6F). Endometrium from arresting conceptus attachment sites had neuronal projection (axonal) at the top of the list of clusters when compared to trophoblast isolated from arresting conceptus attachment sites (Figure 6G); whereas collagen was the most represented group in trophoblast associated with arresting attachment sites, as compared to endometrium from these same sites (Figure 6H). Finally, extracellular matrix factors were highly represented when endometrium from healthy conceptus attachment sites was compared to endometrium from arresting littermate attachment sites (Figure 6I). Endometrium from arresting conceptus attachment sites had a large representation of GRAM domain genes when compared to the endometrium associated with healthy littermates (Figure 6J). The methodology for this study is summarized in Figure 7.

## Discussion

Until recently, small non-coding RNAs were thought to serve no biological purpose, that they were a form of ‘waste’. It is now known that the miRNA system is a vast regulatory network of cellular processes, and that one miRNA can post-transcriptionally silence thousands of mRNA targets [1]. It should come as no surprise then that there are differences in miRNAs found at the ever changing maternal-fetal interface. Here, we have shown that: 1) miRNAs are expressed during early porcine pregnancy, 2) significant differences exist in the miRNA populations found in the various tissues at the maternal-fetal interface, and 3) the endometrium associated with healthy conceptuses does differ from the endometrium associated with arresting conceptuses.

First, non-pregnant endometrium was compared with endometrium from both healthy and arresting conceptus attachment sites in order to gain insight into which miRNAs are important during pregnancy. Several miRNAs which differed between non-pregnant endometrium and endometrium associated with healthy conceptuses also populated the list of miRNAs which differed between non-pregnant endometrium and endometrium associated with arresting conceptuses (Figure 1A, 1B). All followed the same direction of change, and they were similar in magnitude. This indicates that these miRNAs are important for pregnancy, and that the genes and pathways they regulate are involved in processes not related to fetal loss. Quantitative Real-Time PCR results confirmed the microarray findings for several of the miRNAs. Only one miRNA, *miR-323*, was found to have the opposite direction of fold change by PCR when compared to microarray data. Functional clustering was used to predict which groups of genes might be affected by the different populations of miRNAs between non-pregnant and pregnant endometrium. The top functional clusters increased in non-pregnant endometrium (Figure 6A, 6C) were very similar, regardless of whether they were compared to the endometrium associated with healthy or arresting conceptuses. The top five groups between these lists were identical whereas the groups of genes increased in endometrium associated with healthy conceptuses were similar yet different from those increased in endometrium associated with arresting conceptuses when each list was compared to the non-pregnant state. Many of the same groups of genes were found in both lists; however, their order of priority or rank was altered. Positive regulation of transcription ranked higher in endometrium from healthy sites than from arresting sites when each was compared to non-pregnant endometrium. The list of genes in the endometrium from arresting conceptus attachment sites included ubiquitination within its top ten clusters, indicating that degradation of proteins was a priority in this tissue where the attached conceptus was arrested. The similarities between the lists in Figure 6A and 6C indicate which miRNAs and target networks are important in the physiological state of pregnancy, and the miRNAs which are unique to each list merit further attention for their potential role in pregnancy success.

Second, we sought to determine the miRNA expression patterns that define endometrium and trophoblast. Each is a distinct tissue, with endometrium being entirely maternal in origin, and trophoblast being fetal in origin, and thus hemi-allogeneic to its mother. The similarities and differences in miRNA expression between these two tissues were compared to determine which cellular processes were of a higher priority in mother compared with her offspring. Again, several miRNAs were found in both lists (Figure 2A, 2B) regardless of the health status of the offspring. All of the miRNAs which overlapped between lists followed the same direction of change, and in most cases the magnitude was similar. These miRNAs represent part of the fundamental molecular characteristics of maternal and fetal tissue. When healthy endometrium and trophoblast were compared, the top ten functional gene clusters revealed blood vessel development/angiogenesis as the primary cluster in endometrium (Figure 6E) and neuronal differentiation as the primary cluster in trophoblast (Figure 6F). When functional clusters were compared between tissues associated with arresting conceptus attachment sites (Figure 6G), a shift in priority towards processes such as negative regulation of metabolic processes, zinc finger proteins, protein transport, and negative regulation of transcription, which likely contribute to fetal arrest or resorption, was observed in the endometrium. Angiogenesis was not included in the top functional clusters of the arresting endometrium, further supporting our previous work where the endometrium associated with arresting conceptuses lacked angiogenic factors when compared to endometrium associated with healthy littermates [19]–[21], [24].

Third, the miRNA differences between tissues associated with healthy and arresting conceptuses were directly compared, yielding a list of eight miRs which were significantly different between endometrium isolated from a healthy conceptus attachment site endometrium from an arresting conceptus attachment site (Figure 3A). This is the first report of the association between *miR-10a*, *27a*, *29c*, *323*, *331-5p*, *339-3p*, *374-5p*, and *935* and spontaneous fetal arrest. A major interest in miRNA research is the identification of disease biomarkers. It has been shown *in vitro* that trophoblast miRNAs are excreted via exosomes and that the *in vivo* isolation of placenta-specific miRNAs is possible from the maternal circulation [33]. Additionally, another recent report cites *miR-323-3p* as a circulating biomarker of ectopic pregnancy in humans [34]. Our results support the dysregulation of *miR-323* in abnormal conceptus attachment sites and nominate 7 other miRNAs to be further explored as biomarkers of fetal health. To further support our results, *miR-10a* has been linked to the regulation of inflammation in endothelial cells [35], litter size in pigs was negatively associated with a single nucleotide polymorphism (SNP) in *miR-27a* expression [36], and *miR-29c* suppressed migration, invasion, and metastasis in nasopharyngeal carcinoma [37], [38]. In our study, expression of *miR-29c* was elevated in arresting endometrium as compared to healthy. Perhaps trophoblast attachment to the uterine lining was negatively affected by this difference. While none of the changes in our study which were seen by microarray were replicated by PCR, the magnitude of the differences were relatively small. The literature suggests that correlations between microarray and PCR data increased as the magnitude of the fold change increased, and that degrees of change less than two-fold correlated infrequently compared to those above two-fold [39], [40]. All but three of the fold changes observed in our data were below two-fold changes, and this may explain the PCR results. Additionally, our sample number for PCR validation of the miRNA microarray was only three (HE, AE, HT, AT) or four (NP) per group, and statistical analyses indicated the power of the t-tests to be lower than desired. No additional samples where miRNA had been extracted from the tissues were available for this study.

In order to better understand the functional implications of the differences in miRNAs between healthy and arresting endometrium 11 of the putative mRNA targets for these miRNAs were quantified by Real-Time PCR. The genes were targets of miRNAs which were elevated in arresting endometrium as compared to healthy. As miRNAs post-transcriptionally silence their targets, a lower level of mRNA transcripts was expected for all 11 genes. Although no significant differences were seen, all of the genes were depressed in arresting endometrium. Again the statistical power of these tests was sub-optimal, suggesting that differences in transcript levels may be present. Not surprisingly, when the putative mRNA targets were functionally clustered it was revealed that endometrium associated with arresting attachment sites had a distinct order to its list of molecular priorities, and that it included vacuoles, proteolysis, and protein transport. No overlap between the top ten functional clusters was observed between endometrium from healthy attachment sites versus endometrium from an arresting littermate attachment site.

When miRNAs were compared between fetal trophoblast from healthy and arresting attachment sites, no significant differences in any of the 236 miRNAs probed were seen by microarray. This, in addition to our previous work where much of the molecular dysregulation observed at the maternal-fetal interface during spontaneous arrest occurred on the maternal side [19]–[21], [23], [24], and the work of others where the majority of arresting embryos were identified as genetically normal [41], strongly suggests a maternal initiation of conceptus arrest. The triggers remain unknown.

Finally, we are only just beginning to understand the complex web of translational regulation by miRNAs. Here presented is a unique perspective on the miRNA networks found at the maternal-fetal interface, in a species where the independent isolation of fetal and maternal tissues is possible, and where a direct comparison between littermate siblings can be made to determine which factors are key elements that participate in spontaneous fetal loss. We have listed miRNAs which are expressed during early porcine pregnancy, found significant differences in the miRNA populations between maternal and fetal tissue, and implicated the maternal expression of *miR-10a, 27a, 29c, 323, 331-5p, 339-3p, 374b-5p*, and *935* in spontaneous loss during early gestation.

## Supporting Information

Material S1 **Microarray Heat Maps.** Heat maps for microarray comparisons of miRNAs during pregnancy, between endometrium and trophoblast, and between healthy and arresting tissues. AE: arresting endometrium, AT: arresting trophoblast, HE: healthy endometrium, HT: healthy trophoblast, NP: non-pregnant endometrium.
(PDF)

Material S2 **Putative miRNA Targets.** A list of mRNA targets predicted by 6 of 11 target prediction algorithms. Human homologs of the porcine miRNAs which were found to differ by microarray are listed along side of their validated and predicted targets, NCBI RefSeqs, and a description of the gene. N/A indicates that the miRNA was not found in the miRecords database, whereas ‘0 validated interactions’ or ‘0 predicted interactions’ indicates that the miRNA was included in the database, but did not have any targets predicted by 6 of 11 algorithms. Targets for miR-331-5p, 339-3p, and 935 were predicted using only 4 of 11 programs. AE: arresting endometrium, AT: arresting trophoblast, HE: healthy endometrium, HT: healthy trophoblast, NP: non-pregnant endometrium.
(XLS)

Material S3 **Functional Annotation Clusters of Target mRNAs.** Lists of putative mRNA target genes clustered by function and listed by enrichment score over the human genome as a background. AE: arresting endometrium, AT: arresting trophoblast, HE: healthy endometrium, HT: healthy trophoblast, NP: non-pregnant endometrium.
(XLS)

Material S4 **Functional Classifications of Target mRNAs.** Gene lists of putative mRNA targets grouped and ranked according to the number of times functionally related genes were represented within each list. Gd: gestation day.
(DOC)

Material S5 **Functional Annotation Charts of Target mRNAs.** Charts of mRNA targets listing the magnitude of enrichment of the genes in the submitted lists compared to the human genome as a background. AE: arresting endometrium, AT: arresting trophoblast, HE: healthy endometrium, HT: healthy trophoblast, NP: non-pregnant endometrium.
(XLS)
