# Supplementary material for: The MicroRNAome of Pregnancy: Deciphering miRNA Networks at the Maternal-Fetal Interface
Source: PLoS One. 2013 Nov 22;8(11):e72264. doi: 10.1371/journal.pone.0072264 (PMC3838410; doi:10.1371/journal.pone.0072264)
Supplement: Material S1 — Microarray Heat Maps. Heat maps for microarray comparisons of miRNAs during pregnancy, between endometrium and trophoblast, and between healthy and arresting tissues. AE: arresting endometrium, AT: arresting trophoblast, HE: healthy endometrium, HT: healthy trophoblast, NP: non-pregnant endometrium. (PDF) [file pone.0072264.s001.pdf]

1. Clustering graph of all miRNAs with signal intensity > 32 (Endometrium associated with arresting conceptuses (AE) and Endometrium associated with healthy conceptuses (HE)

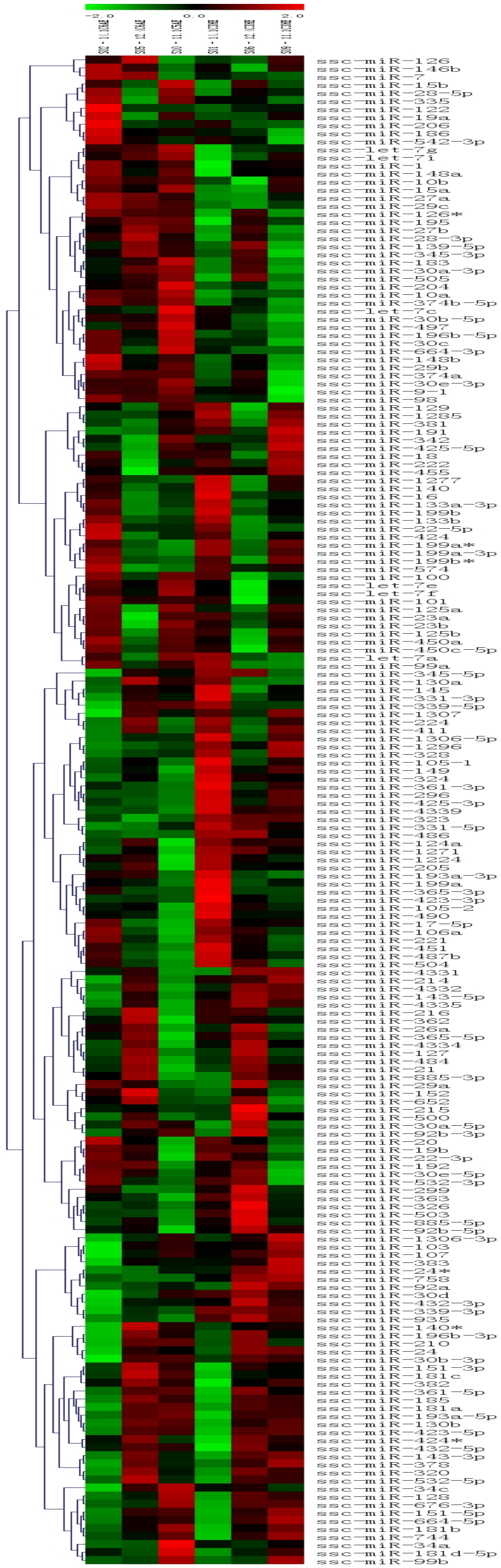

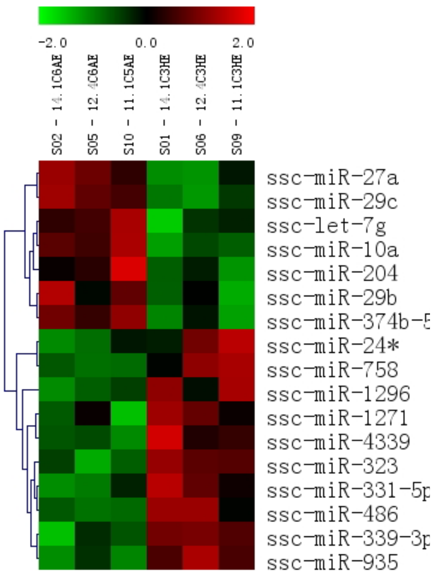

p-value < 0.10

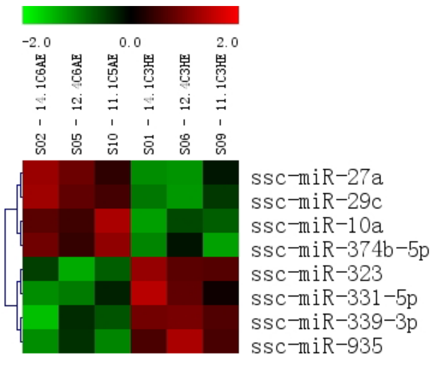

p-value < 0.05

2.Clustering graph of all miRNAs with signal intensity > 32 (Endometrium from nonpregnant animals (NP) and Endometrium associated with healthy conceptuses (HE)

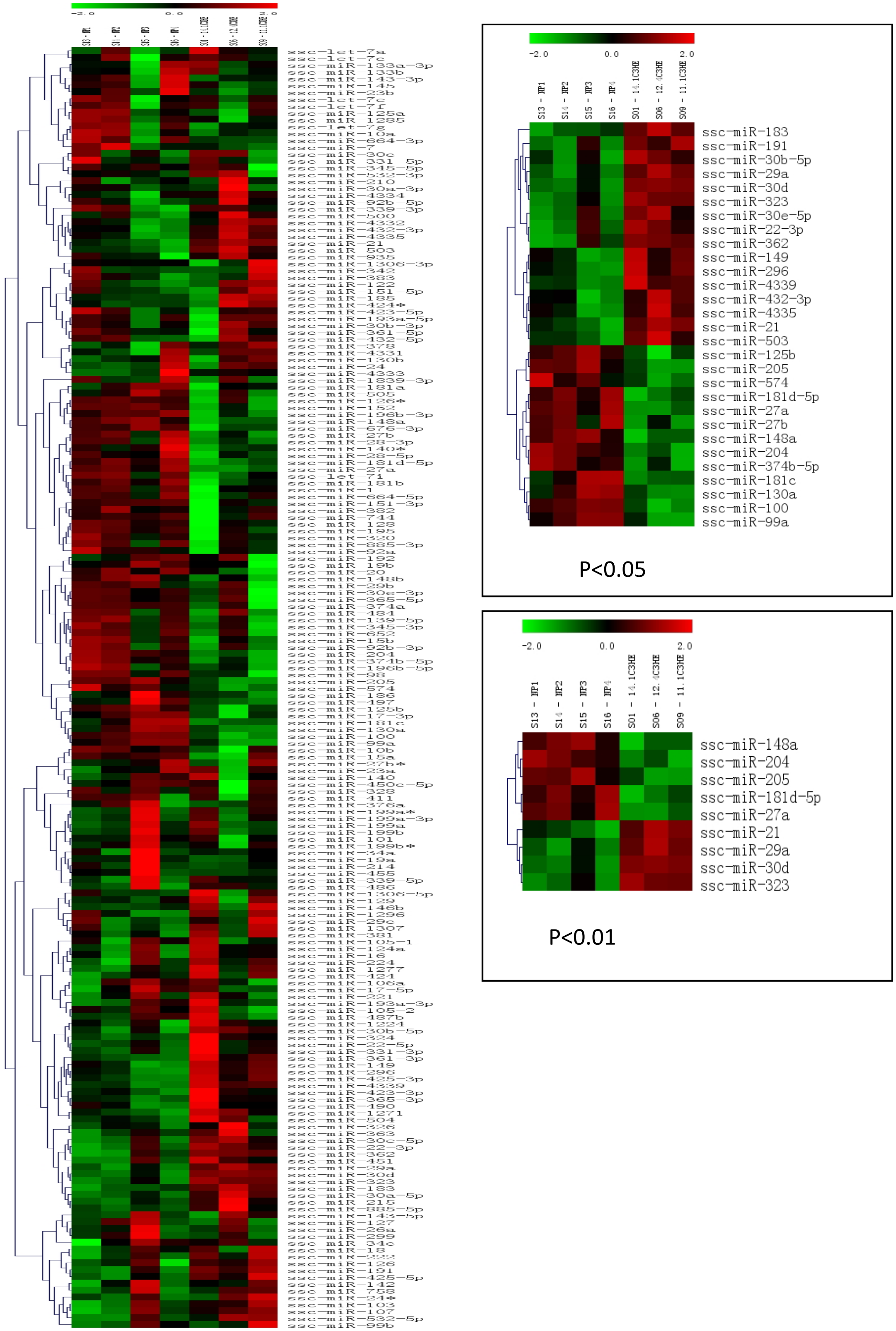

3.Clustering graph of all miRNAs with signal intensity > 32 (Trophoblast from healthy conceptuses (HT) and endometrium associated with healthy conceptuses (HE)

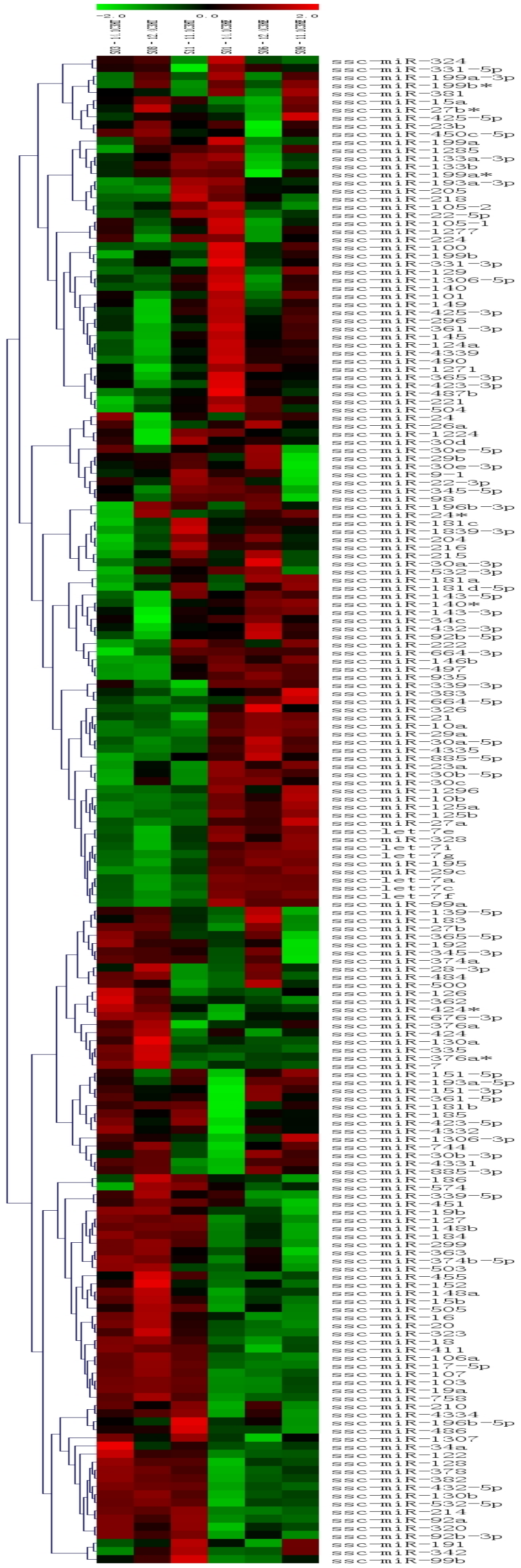

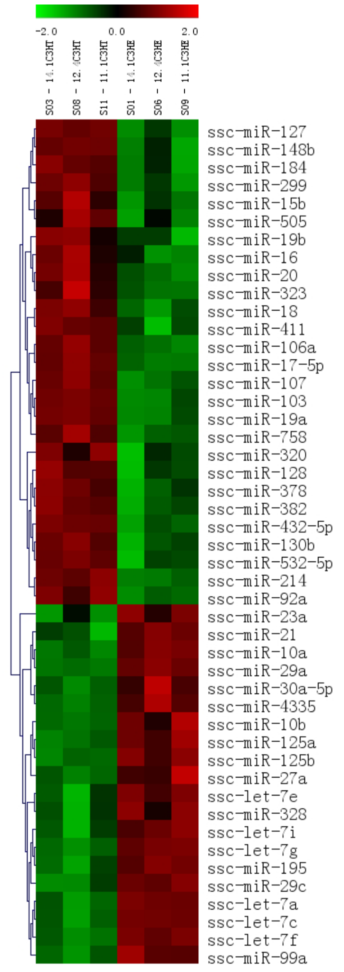

P<0.05

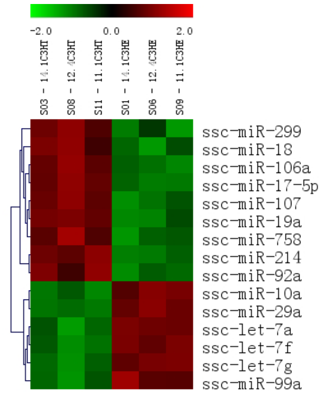

P<0.01

4.Clustering graph of all miRNAs with signal intensity > 32 (Trophoblast from arresting conceptuses (AT) and endometrium associated with arresting conceptuses (AE)

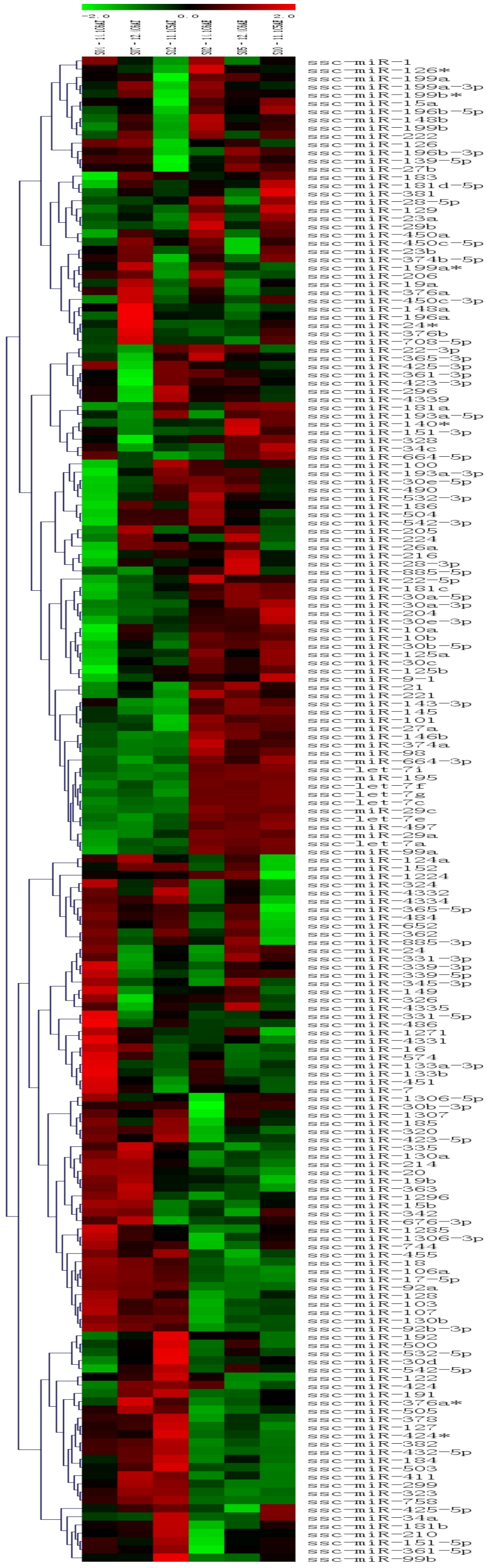

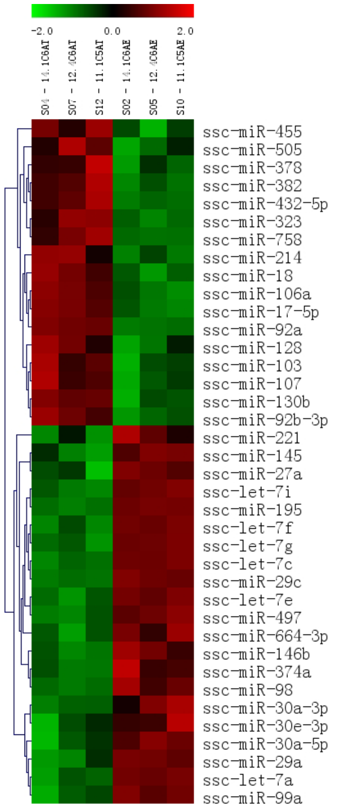

P<0.05

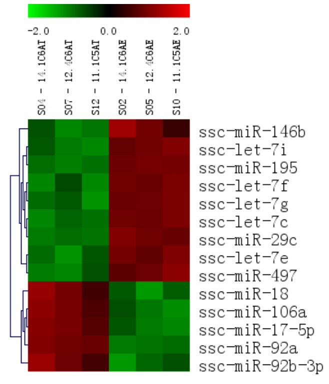

P<0.01

5.Clustering graph of all miRNAs with signal intensity > 32 (Trophoblast (T) and Endometrium (E)

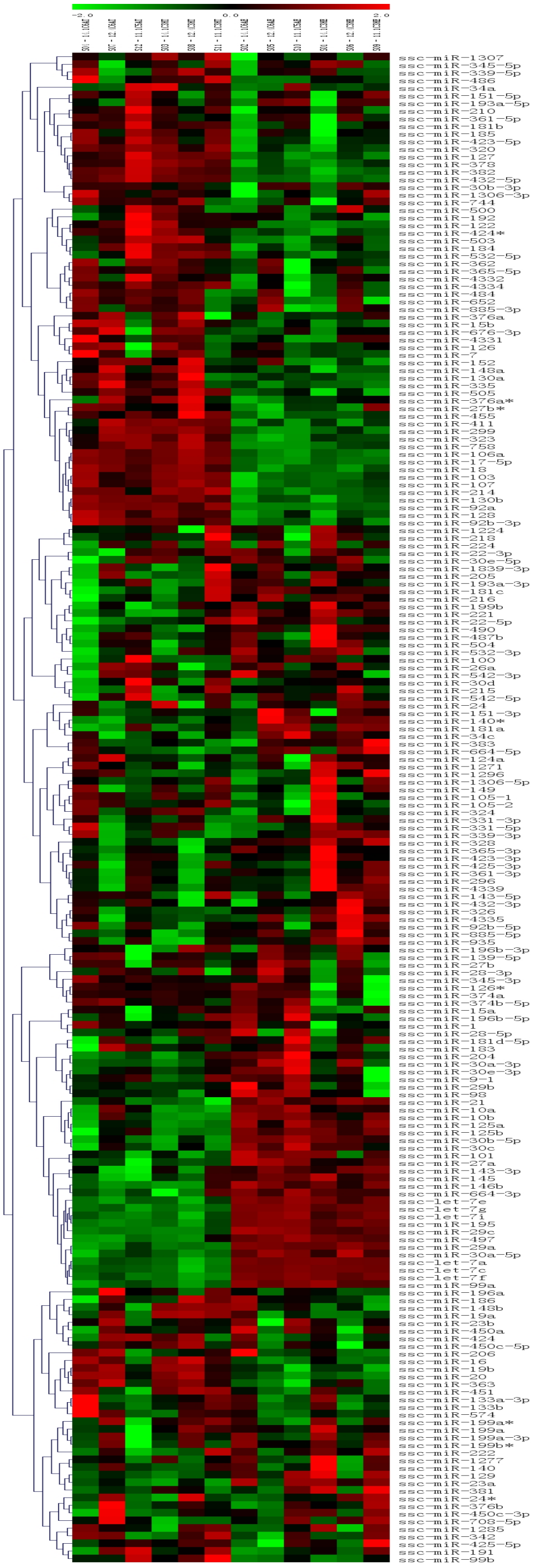

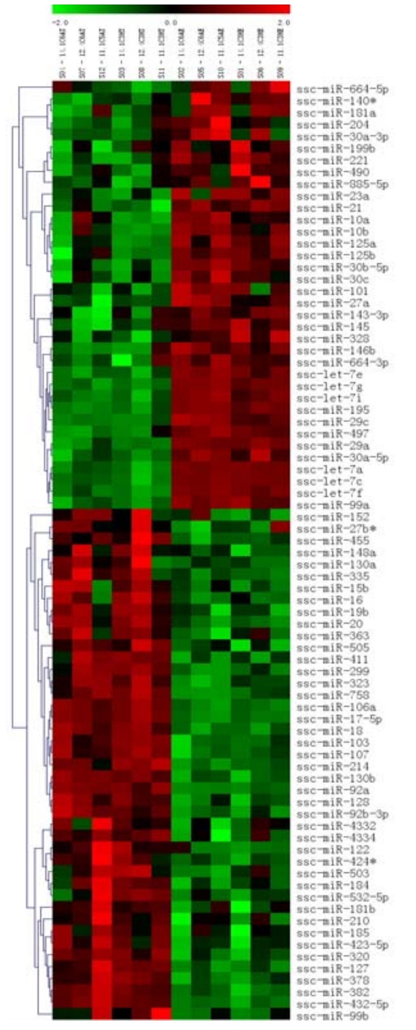

P<0.05

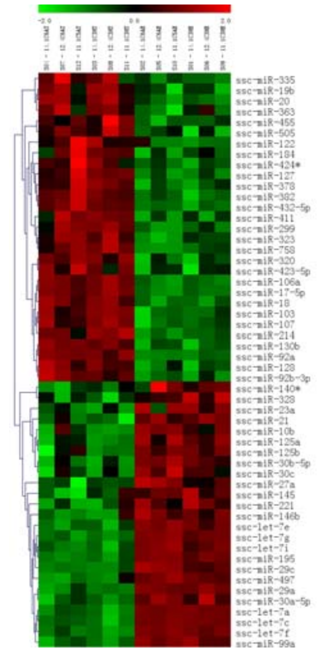

P<0.01
