# Supplementary material for: The MicroRNAome of Pregnancy: Deciphering miRNA Networks at the Maternal-Fetal Interface
Source: PLoS One. 2013 Nov 22;8(11):e72264. doi: 10.1371/journal.pone.0072264 (PMC3838410; doi:10.1371/journal.pone.0072264)
Supplement: Material S4 — Functional Classifications of Target mRNAs. Gene lists of putative mRNA targets grouped and ranked according to the number of times functionally related genes were represented within each list. Gd: gestation day. (DOC) [file pone.0072264.s004.doc]

**Non-Pregnant Endometrium > Healthy gd20 Endometrium**

**Gene Functional Classification Result**

**Gene Group 1 Enrichment Score: 10.02023800893443**

**REFSEQ_MRNA Gene Name**

NM_006022 TSC22 domain family, member 1

NM_006465 AT rich interactive domain 3B (BRIGHT-like)

NM_152622 mesoderm induction early response 1, family member 3

NM_030759 nuclear receptor binding factor 2

NM_022913 GC-rich promoter binding protein 1

NM_015534 zinc finger, ZZ-type containing 3

NM_181782 nuclear receptor coactivator 7

NM_015608 chromosome 10 open reading frame 137

**Gene Group 2 Enrichment Score: 8.690113468006867**

**REFSEQ_MRNA Gene Name**

NM_020310 MAX binding protein

NM_001427 engrailed homeobox 2

NM_004229 mediator complex subunit 14

NM_015265 SATB homeobox 2

NM_199072 MyoD family inhibitor domain containing

NM_001426 engrailed homeobox 1

NM_021961 TEA domain family member 1 (SV40 transcriptional enhancer factor)

NM_001452 forkhead box F2

NM_203394 E2F transcription factor 7

NM_004379 cAMP responsive element binding protein 1

NM_018951 homeobox A10

NM_022062 PBX/knotted 1 homeobox 2

NM_005599 nescient helix loop helix 2

NM_005924 mesenchyme homeobox 2

NM_001987 ets variant 6

NM_005316 general transcription factor IIH, polypeptide 1, 62kDa

NM_153607 chromosome 5 open reading frame 41

NM_057175 NMDA receptor regulated 1

NM_022658 homeobox C8

NM_019102 homeobox A5

NM_015995 Kruppel-like factor 13

NM_012081 elongation factor, RNA polymerase II, 2

NM_021728 orthodenticle homeobox 2

NM_012391 SAM pointed domain containing ets transcription factor

NM_005230 ELK3, ETS-domain protein (SRF accessory protein 2)

NM_006022 TSC22 domain family, member 1

NM_053002 mediator complex subunit 12-like

NM_007249 Kruppel-like factor 12

NM_022728 neurogenic differentiation 6

NM_032440 ligand dependent nuclear receptor corepressor

NM_022913 GC-rich promoter binding protein 1

NM_002126 hepatic leukemia factor

NM_021809 TGFB-induced factor homeobox 2

NM_006164 nuclear factor (erythroid-derived 2)-like 2

NM_006195 pre-B-cell leukemia homeobox 3

NM_030661 homeobox A3

NM_005461 v-maf musculoaponeurotic fibrosarcoma oncogene homolog B (avian)

NM_003205 transcription factor 12

NM_001001419 SMAD family member 5

NM_003670 basic helix-loop-helix family, member e40

**Gene Group 3 Enrichment Score: 5.886426579531085**

**REFSEQ_MRNA Gene Name**

NM_006981 nuclear receptor subfamily 4, group A, member 3

NM_003822 nuclear receptor subfamily 5, group A, member 2

NM_001001890 runt-related transcription factor 1

NM_001300 Kruppel-like factor 6

NM_012231 PR domain containing 2, with ZNF domain

NM_006951 TAF5 RNA polymerase II, TATA box binding protein (TBP)-associated factor, 100kDa

NM_005238 v-ets erythroblastosis virus E26 oncogene homolog 1 (avian)

NM_006940 SRY (sex determining region Y)-box 5

NM_003111 Sp3 transcription factor

NM_012308 lysine (K)-specific demethylase 2A

NM_015995 Kruppel-like factor 13

NM_032440 ligand dependent nuclear receptor corepressor

NM_000964 retinoic acid receptor, alpha

NM_005234 nuclear receptor subfamily 2, group F, member 6

NM_004229 mediator complex subunit 14

NM_014795 zinc finger E-box binding homeobox 2

NM_005230 ELK3, ETS-domain protein (SRF accessory protein 2)

NM_004973 jumonji, AT rich interactive domain 2

NM_176814 zinc finger protein 800

NM_003107 SRY (sex determining region Y)-box 4

NM_001806 CCAAT/enhancer binding protein (C/EBP), gamma

NM_006164 nuclear factor (erythroid-derived 2)-like 2

NM_018433 lysine (K)-specific demethylase 3A

NM_006352 zinc finger protein 238

NM_004992 methyl CpG binding protein 2 (Rett syndrome)

NM_018263 additional sex combs like 2 (Drosophila)

NM_000176 nuclear receptor subfamily 3, group C, member 1 (glucocorticoid receptor)

NM_002198 interferon regulatory factor 1

NM_003670 basic helix-loop-helix family, member e40

NM_003413 Zic family member 3 (odd-paired homolog, Drosophila)

NM_006734 human immunodeficiency virus type I enhancer binding protein 2

NM_014682 suppression of tumorigenicity 18 (breast carcinoma) (zinc finger protein)

NM_015534 zinc finger, ZZ-type containing 3

NM_000248 microphthalmia-associated transcription factor

NM_033013 nuclear receptor subfamily 1, group I, member 2

NM_001438 estrogen-related receptor gamma

NM_015481 zinc finger protein 385A

NM_006599 nuclear factor of activated T-cells 5, tonicity-responsive

NM_012234 RING1 and YY1 binding protein

NM_005461 v-maf musculoaponeurotic fibrosarcoma oncogene homolog B (avian)

NM_001452 forkhead box F2

NM_002893 retinoblastoma binding protein 7

NM_002166 inhibitor of DNA binding 2, dominant negative helix-loop-helix protein

NM_002460 interferon regulatory factor 4

NM_001001395 LIM domain only 3 (rhombotin-like 2)

NM_032682 forkhead box P1

NM_030751 zinc finger E-box binding homeobox 1

NM_138473 Sp1 transcription factor

NM_002167 inhibitor of DNA binding 3, dominant negative helix-loop-helix protein

NM_022658 homeobox C8

NM_014491 forkhead box P2

NM_005955 metal-regulatory transcription factor 1

NM_007249 Kruppel-like factor 12

NM_015094 hypermethylated in cancer 2

NM_021961 TEA domain family member 1 (SV40 transcriptional enhancer factor)

NM_015069 zinc finger protein 423

NM_152991 embryonic ectoderm development

NM_000901 nuclear receptor subfamily 3, group C, member 2

NM_006526 zinc finger protein 217

NM_014079 Kruppel-like factor 15

NM_000522 homeobox A13

NM_001489 nuclear receptor subfamily 6, group A, member 1

NM_014969 WD repeat domain 47

NM_003112 Sp4 transcription factor

NM_033224 purine-rich element binding protein B

NM_000125 estrogen receptor 1

NM_005316 general transcription factor IIH, polypeptide 1, 62kDa

NM_015265 SATB homeobox 2

NM_004241 jumonji domain containing 1C

NM_002500 neurogenic differentiation 1

NM_181353 inhibitor of DNA binding 1, dominant negative helix-loop-helix protein

NM_032329 inhibitor of growth family, member 5

NM_002655 pleiomorphic adenoma gene 1

NM_003575 zinc finger protein 282

NM_004926 zinc finger protein 36, C3H type-like 1

**Gene Group 4 Enrichment Score: 5.612915317600619**

**REFSEQ_MRNA Gene Name**

NM_003721 regulatory factor X-associated ankyrin-containing protein

NM_015215 calmodulin binding transcription activator 1

NM_022913 GC-rich promoter binding protein 1

NM_015158 KN motif and ankyrin repeat domains 1; similar to ankyrin repeat domain protein 15 isoform b

**Gene Group 5 Enrichment Score: 4.881665705100076**

**REFSEQ_MRNA Gene Name**

NM_025134 chromodomain helicase DNA binding protein 9

NM_003173 suppressor of variegation 3-9 homolog 1 (Drosophila)

NM_175709 chromobox homolog 7

NM_004824 chromodomain protein, Y-like

**Gene Group 6 Enrichment Score: 3.8112205129794807**

**REFSEQ_MRNA Gene Name**

NM_013396 ubiquitin specific peptidase 25

NM_020357 PEST proteolytic signal containing nuclear protein

NM_004656 BRCA1 associated protein-1 (ubiquitin carboxy-terminal hydrolase)

NM_015017 ubiquitin specific peptidase 33

NM_203301 F-box protein 33

NM_022832 ubiquitin specific peptidase 46

NM_014554 SUMO1/sentrin specific peptidase 1

NM_001001664 speckle-type POZ protein-like

NM_018315 F-box and WD repeat domain containing 7

NM_032236 ubiquitin specific peptidase 48

NM_004232 suppressor of cytokine signaling 6

NM_015176 F-box protein 28

NM_015246 mahogunin, ring finger 1

**Gene Group 7 Enrichment Score: 3.674723622461432**

**REFSEQ_MRNA Gene Name**

NM_015271 tripartite motif-containing 2

NM_005180 BMI1 polycomb ring finger oncogene

NM_139177 solute carrier family 39 (metal ion transporter), member 11

NM_015246 mahogunin, ring finger 1

NM_015336 zinc finger, DHHC-type containing 17

NM_144726 ring finger protein 145

NM_001001395 LIM domain only 3 (rhombotin-like 2)

NM_152737 ring finger protein 182

NM_152271 LON peptidase N-terminal domain and ring finger 1

NM_014504 RAB guanine nucleotide exchange factor (GEF) 1

NM_015153 PHD finger protein 3

NM_006526 zinc finger protein 217

NM_018375 solute carrier family 39 (zinc transporter), member 9

NM_025126 ring finger protein 34

NM_003575 zinc finger protein 282

NM_004241 jumonji domain containing 1C

NM_006458 tripartite motif-containing 3

NM_004776 UDP-Gal:betaGlcNAc beta 1,4- galactosyltransferase, polypeptide 5

NM_018263 additional sex combs like 2 (Drosophila)

NM_023076 unkempt homolog (Drosophila)-like

NM_004293 guanine deaminase

NM_001002909 G patch domain containing 8

NM_014819 praja ring finger 2

NM_015017 ubiquitin specific peptidase 33

NM_015534 zinc finger, ZZ-type containing 3

NM_015481 zinc finger protein 385A

NM_022781 ring finger protein 38

NM_152787 mitogen-activated protein kinase kinase kinase 7 interacting protein 3

NM_153812 PHD finger protein 13

NM_176814 zinc finger protein 800

NM_014615 KIAA0182

NM_007218 ring finger protein 139

**Gene Group 8 Enrichment Score: 3.5681765927866675**

**REFSEQ_MRNA Gene Name**

NM_018211 ribonucleoprotein, PTB-binding 2

NM_016090 RNA binding motif protein 7

NM_019027 RNA binding motif protein 47

NM_007007 cleavage and polyadenylation specific factor 6, 68kDa

NM_030627 cytoplasmic polyadenylation element binding protein 4

NM_001420 ELAV (embryonic lethal, abnormal vision, Drosophila)-like 3 (Hu antigen C)

**Gene Group 9 Enrichment Score: 3.500029944365567**

**REFSEQ_MRNA Gene Name**

NM_003390 WEE1 homolog (S. pombe)

NM_006622 polo-like kinase 2 (Drosophila)

NM_032430 BR serine/threonine kinase 1

NM_173354 salt-inducible kinase 1

NM_101395 dual-specificity tyrosine-(Y)-phosphorylation regulated kinase 1A

NM_014683 unc-51-like kinase 2 (C. elegans)

NM_017553 INO80 homolog (S. cerevisiae)

NM_003010 mitogen-activated protein kinase kinase 4

NM_004755 ribosomal protein S6 kinase, 90kDa, polypeptide 5

NM_005400 protein kinase C, epsilon

NM_198465 Nik related kinase

NM_014397 NIMA (never in mitosis gene a)-related kinase 6

NM_002314 LIM domain kinase 1

NM_006141 dynein, cytoplasmic 1, light intermediate chain 2

NM_014899 Rho-related BTB domain containing 3

NM_002401 mitogen-activated protein kinase kinase kinase 3

NM_006251 protein kinase, AMP-activated, alpha 1 catalytic subunit

NM_001259 cyclin-dependent kinase 6

NM_005813 protein kinase D3

NM_020778 alpha-kinase 3

NM_012424 ribosomal protein S6 kinase, 52kDa, polypeptide 1

NM_005433 v-yes-1 Yamaguchi sarcoma viral oncogene homolog 1

NM_175854 PAN3 poly(A) specific ribonuclease subunit homolog (S. cerevisiae)

NM_015076 cell division cycle 2-like 6 (CDK8-like)

NM_003607 CDC42 binding protein kinase alpha (DMPK-like)

NM_002031 fyn-related kinase

NM_001895 casein kinase 2, alpha 1 polypeptide pseudogene; casein kinase 2, alpha 1 polypeptide

NM_006852 tousled-like kinase 2

NM_013233 serine threonine kinase 39 (STE20/SPS1 homolog, yeast)

NM_018323 phosphatidylinositol 4-kinase type 2 beta

**Gene Group 10 Enrichment Score: 3.374518696306157**

**REFSEQ_MRNA Gene Name**

NM_201567 cell division cycle 25 homolog A (S. pombe)

NM_152510 HORMA domain containing 2

NM_005862 stromal antigen 1

NM_001241 cyclin T2

NM_003858 cyclin K

NM_004060 cyclin G1

**Gene Group 11 Enrichment Score: 3.333941707407138**

**REFSEQ_MRNA Gene Name**

NM_030816 ankyrin repeat domain 13C

NM_032105 protein phosphatase 1, regulatory (inhibitor) subunit 12B

NM_175873 ankyrin repeat domain 43

NM_015158 KN motif and ankyrin repeat domains 1; similar to ankyrin repeat domain protein 15 isoform b

NM_033121 ankyrin repeat domain 13A

NM_002480 protein phosphatase 1, regulatory (inhibitor) subunit 12A

**Gene Group 12 Enrichment Score: 3.3187272716912997**

**REFSEQ_MRNA Gene Name**

NM_001406 ephrin-B3

NM_175607 contactin 4

NM_003872 neuropilin 2

NM_003873 neuropilin 1

NM_020796 sema domain, transmembrane domain (TM), and cytoplasmic domain, (semaphorin) 6A

**Gene Group 13 Enrichment Score: 2.890234892847874**

**REFSEQ_MRNA Gene Name**

NM_001967 similar to eukaryotic translation initiation factor 4A2; eukaryotic translation initiation factor 4A, isoform 2

NM_025134 chromodomain helicase DNA binding protein 9

NM_003972 BTAF1 RNA polymerase II, B-TFIID transcription factor-associated, 170kDa (Mot1 homolog, S. cerevisiae)

NM_017780 chromodomain helicase DNA binding protein 7

NM_017553 INO80 homolog (S. cerevisiae)

NM_004396 DEAD (Asp-Glu-Ala-Asp) box polypeptide 5

NM_003069 SWI/SNF related, matrix associated, actin dependent regulator of chromatin, subfamily a, member 1

**Gene Group 14 Enrichment Score: 2.727810006028458**

**REFSEQ_MRNA Gene Name**

NM_004440 EPH receptor A7

NM_006206 platelet-derived growth factor receptor, alpha polypeptide

NM_004448 v-erb-b2 erythroblastic leukemia viral oncogene homolog 2, neuro/glioblastoma derived oncogene homolog (avian)

NM_001007156 neurotrophic tyrosine kinase, receptor, type 3

NM_005228 epidermal growth factor receptor (erythroblastic leukemia viral (v-erb-b) oncogene homolog, avian)

NM_001982 v-erb-b2 erythroblastic leukemia viral oncogene homolog 3 (avian)

**Gene Group 15 Enrichment Score: 2.7225819636662276**

**REFSEQ_MRNA Gene Name**

NM_002833 protein tyrosine phosphatase, non-receptor type 9

NM_004419 dual specificity phosphatase 5

NM_014369 protein tyrosine phosphatase, non-receptor type 18 (brain-derived)

NM_003463 protein tyrosine phosphatase type IVA, member 1

**Gene Group 16 Enrichment Score: 2.4334554313673786**

**REFSEQ_MRNA Gene Name**

NM_052851 StAR-related lipid transfer (START) domain containing 13

NM_020824 Rho GTPase activating protein 21

NM_016603 family with sequence similarity 13, member B

NM_018287 Rho GTPase activating protein 12

**Gene Group 17 Enrichment Score: 2.1108395893312855**

**REFSEQ_MRNA Gene Name**

NM_001616 activin A receptor, type IIA

NM_001204 bone morphogenetic protein receptor, type II (serine/threonine kinase)

NM_001105 activin A receptor, type I

NM_005813 protein kinase D3

**Gene Group 18 Enrichment Score: 2.065118157511815**

**REFSEQ_MRNA Gene Name**

NM_015263 Dmx-like 2

NM_017974 ATG16 autophagy related 16-like 1 (S. cerevisiae)

NM_006726 LPS-responsive vesicle trafficking, beach and anchor containing

NM_203301 F-box protein 33

NM_014325 coronin, actin binding protein, 1C

NM_005720 actin related protein 2/3 complex, subunit 1B, 41kDa; similar to Actin-related protein 2/3 complex subunit 1B (ARP2/3 complex 41 kDa subunit) (p41-ARC)

NM_001001664 speckle-type POZ protein-like

NM_152991 embryonic ectoderm development

NM_015626 WD repeat and SOCS box-containing 1

NM_005509 Dmx-like 1

NM_018315 F-box and WD repeat domain containing 7

NM_015176 F-box protein 28

NM_014969 WD repeat domain 47

**Gene Group 19 Enrichment Score: 1.9869960331019443**

**REFSEQ_MRNA Gene Name**

NM_021183 RAP2C, member of RAS oncogene family

NM_016131 RAB10, member RAS oncogene family

NM_016544 DnaJ (Hsp40) homolog, subfamily C, member 27

NM_004162 RAB5A, member RAS oncogene family

NM_004663 RAB11A, member RAS oncogene family

NM_014488 RAB30, member RAS oncogene family

NM_031934 RAB34, member RAS oncogene family

NM_020673 RAB22A, member RAS oncogene family

**Gene Group 20 Enrichment Score: 1.9183466806491194**

**REFSEQ_MRNA Gene Name**

NM_018211 ribonucleoprotein, PTB-binding 2

NM_006924 splicing factor, arginine/serine-rich 1

NM_007007 cleavage and polyadenylation specific factor 6, 68kDa

NM_002515 neuro-oncological ventral antigen 1

NM_007375 TAR DNA binding protein

NM_005105 RNA binding motif protein 8A

NM_004768 splicing factor, arginine/serine-rich 11

NM_004966 heterogeneous nuclear ribonucleoprotein F

**Gene Group 21 Enrichment Score: 1.6798893935795929**

**REFSEQ_MRNA Gene Name**

NM_058187 chromosome 21 open reading frame 63

NM_001781 CD69 molecule

NM_005907 mannosidase, alpha, class 1A, member 1

NM_014373 G protein-coupled receptor 160

NM_003654 carbohydrate (keratan sulfate Gal-6) sulfotransferase 1

NM_002033 fucosyltransferase 4 (alpha (1,3) fucosyltransferase, myeloid-specific)

NM_152996 ST6 (alpha-N-acetyl-neuraminyl-2,3-beta-galactosyl-1,3)-N-acetylgalactosaminide alpha-2,6-sialyltransferase 3

NM_005776 cornichon homolog (Drosophila)

NM_005668 ST8 alpha-N-acetyl-neuraminide alpha-2,8-sialyltransferase 4

NM_015879 ST8 alpha-N-acetyl-neuraminide alpha-2,8-sialyltransferase 3

NM_004776 UDP-Gal:betaGlcNAc beta 1,4- galactosyltransferase, polypeptide 5

NM_016548 golgi membrane protein 1

NM_003779 UDP-Gal:betaGlcNAc beta 1,4- galactosyltransferase, polypeptide 3

NM_017423 UDP-N-acetyl-alpha-D-galactosamine:polypeptide N-acetylgalactosaminyltransferase 7 (GalNAc-T7)

NM_014283 chromosome 1 open reading frame 9

NM_032039 integrin alpha FG-GAP repeat containing 3

NM_004616 tetraspanin 8

NM_005277 glycoprotein M6A

NM_003782 UDP-Gal:betaGlcNAc beta 1,3-galactosyltransferase, polypeptide 4

**Gene Group 22 Enrichment Score: 1.6132485587175216**

**REFSEQ_MRNA Gene Name**

NM_025187 chromosome 16 open reading frame 70

NM_001002243 aftiphilin

NM_014822 SEC24 family, member D (S. cerevisiae)

NM_001655 archain 1

NM_004859 clathrin, heavy chain (Hc)

**Gene Group 23 Enrichment Score: 1.3561982484061126**

**REFSEQ_MRNA Gene Name**

NM_003337 ubiquitin-conjugating enzyme E2B (RAD6 homolog)

NM_017582 ubiquitin-conjugating enzyme E2Q family member 1

NM_003348 ubiquitin-conjugating enzyme E2N (UBC13 homolog, yeast)

NM_194261 ubiquitin-conjugating enzyme E2I (UBC9 homolog, yeast)

NM_080678 ubiquitin-conjugating enzyme E2F (putative)

NM_003338 ubiquitin-conjugating enzyme E2D 1 (UBC4/5 homolog, yeast)

**Gene Group 24 Enrichment Score: 1.1647146239552921**

**REFSEQ_MRNA Gene Name**

NM_001677 ATPase, Na+/K+ transporting, beta 1 polypeptide

NM_001682 ATPase, Ca++ transporting, plasma membrane 1

NM_001017971 ATPase, H+ transporting, lysosomal accessory protein 1-like

NM_001693 ATPase, H+ transporting, lysosomal 56/58kDa, V1 subunit B2

NM_020453 ATPase, class V, type 10D

**Gene Group 25 Enrichment Score: 1.139603848009399**

**REFSEQ_MRNA Gene Name**

NM_002204 integrin, alpha 3 (antigen CD49C, alpha 3 subunit of VLA-3 receptor)

NM_002998 syndecan 2

NM_018910 protocadherin alpha 7

NM_018906 protocadherin alpha 3

NM_058187 chromosome 21 open reading frame 63

NM_001781 CD69 molecule

NM_014373 G protein-coupled receptor 160

NM_013281 fibronectin leucine rich transmembrane protein 3

NM_014293 neuronal pentraxin receptor

NM_002205 integrin, alpha 5 (fibronectin receptor, alpha polypeptide)

NM_006811 serine incorporator 3

NM_018930 protocadherin beta 10; protocadherin beta 9

NM_018908 protocadherin alpha 5

NM_012428 neuroplastin

NM_001004439 integrin, alpha 11

NM_018905 protocadherin alpha 2

NM_033655 contactin associated protein-like 3; contactin associated protein-like 3B

NM_018976 solute carrier family 38, member 2

NM_016548 golgi membrane protein 1

NM_018903 protocadherin alpha 12

NM_018904 protocadherin alpha 13

NM_018899 protocadherin alpha 10

NM_018898 protocadherin alpha subfamily C, 1; protocadherin alpha subfamily C, 2

NM_001795 cadherin 5, type 2 (vascular endothelium)

NM_032039 integrin alpha FG-GAP repeat containing 3

NM_001797 cadherin 11, type 2, OB-cadherin (osteoblast)

NM_002214 integrin, beta 8

NM_020403 protocadherin 9

NM_018911 protocadherin alpha 8; protocadherin alpha 6

NM_182527 calcium binding protein 7

NM_002644 polymeric immunoglobulin receptor

NM_001792 cadherin 2, type 1, N-cadherin (neuronal)

NM_003144 signal sequence receptor, alpha

NM_018900 protocadherin alpha 1

NM_018907 protocadherin alpha 4

NM_002207 integrin, alpha 9

**Gene Group 26 Enrichment Score: 1.1133046053377984**

**REFSEQ_MRNA Gene Name**

NM_001677 ATPase, Na+/K+ transporting, beta 1 polypeptide

NM_018976 solute carrier family 38, member 2

NM_020689 solute carrier family 24 (sodium/potassium/calcium exchanger), member 3

NM_022058 solute carrier family 4, sodium bicarbonate transporter, member 10

**Gene Group 27 Enrichment Score: 0.9121579606876826**

**REFSEQ_MRNA Gene Name**

NM_058187 chromosome 21 open reading frame 63

NM_018908 protocadherin alpha 5

NM_002998 syndecan 2

NM_144599 non imprinted in Prader-Willi/Angelman syndrome 1

NM_147127 Ellis van Creveld syndrome 2

NM_032039 integrin alpha FG-GAP repeat containing 3

NM_138799 membrane bound O-acyltransferase domain containing 2

NM_020403 protocadherin 9

NM_005779 lipoma HMGIC fusion partner-like 2

NM_014283 chromosome 1 open reading frame 9

NM_015008 transmembrane and coiled-coil domain family 1

NM_182511 cerebellin 2 precursor

NM_001003674 chromosome 18 open reading frame 1

NM_001781 CD69 molecule

NM_016040 transmembrane emp24 protein transport domain containing 5

NM_006134 transmembrane protein 50B

NM_152737 ring finger protein 182

NM_014373 G protein-coupled receptor 160

NM_018375 solute carrier family 39 (zinc transporter), member 9

NM_005277 glycoprotein M6A

NM_005776 cornichon homolog (Drosophila)

NM_017911 family with sequence similarity 118, member A

NM_138340 abhydrolase domain containing 3

NM_198549 family with sequence similarity 73, member A

NM_000958 prostaglandin E receptor 4 (subtype EP4)

NM_003943 starch binding domain 1

NM_018905 protocadherin alpha 2

NM_002644 polymeric immunoglobulin receptor

NM_181836 transmembrane emp24 protein transport domain containing 7; toll-like receptor adaptor molecule 2

NM_017694 major facilitator superfamily domain containing 6

NM_178454 DNA-damage regulated autophagy modulator 2

NM_153365 transmembrane anterior posterior transformation 1

NM_016548 golgi membrane protein 1

NM_020182 prostate transmembrane protein, androgen induced 1

NM_004694 solute carrier family 16, member 6 (monocarboxylic acid transporter 7); similar to solute carrier family 16, member 6

NM_016072 golgi transport 1 homolog B (S. cerevisiae)

NM_031434 transmembrane and ubiquitin-like domain containing 1

NM_017786 Golgi-localized protein

NM_019556 motile sperm domain containing 1

NM_020123 transmembrane 9 superfamily member 3

NM_006811 serine incorporator 3

NM_021978 suppression of tumorigenicity 14 (colon carcinoma)

NM_012329 monocyte to macrophage differentiation-associated

NM_018910 protocadherin alpha 7

NM_080546 solute carrier family 44, member 1

NM_022484 transmembrane protein 168

NM_020644 TMEM9 domain family, member B

NM_013281 fibronectin leucine rich transmembrane protein 3

**Gene Group 28 Enrichment Score: 0.9096380233022326**

**REFSEQ_MRNA Gene Name**

NM_005065 sel-1 suppressor of lin-12-like (C. elegans)

NM_016040 transmembrane emp24 protein transport domain containing 5

NM_020123 transmembrane 9 superfamily member 3

NM_014283 chromosome 1 open reading frame 9

NM_053039 UDP glucuronosyltransferase 2 family, polypeptide B28

NM_001077 UDP glucuronosyltransferase 2 family, polypeptide B17

NM_001076 UDP glucuronosyltransferase 2 family, polypeptide B15

**Gene Group 29 Enrichment Score: 0.5719869124727991**

**REFSEQ_MRNA Gene Name**

NM_020116 follistatin-like 5

NM_001219 calumenin

NM_182527 calcium binding protein 7

NM_022138 SPARC related modular calcium binding 2

NM_012198 grancalcin, EF-hand calcium binding protein

**Gene Group 30 Enrichment Score: 0.43314948891806987**

**REFSEQ_MRNA Gene Name**

NM_006310 hypothetical protein FLJ11822; aminopeptidase puromycin sensitive

NM_197941 ADAM metallopeptidase with thrombospondin type 1 motif, 6

NM_006988 ADAM metallopeptidase with thrombospondin type 1 motif, 1

NM_199355 ADAM metallopeptidase with thrombospondin type 1 motif, 18

**Gene Group 31 Enrichment Score: 0.36930750490700553**

**REFSEQ_MRNA Gene Name**

NM_052910 SLIT and NTRK-like family, member 1

NM_020873 leucine rich repeat neuronal 1

NM_138440 vasorin

NM_005512 leucine rich repeat containing 32

NM_015541 leucine-rich repeats and immunoglobulin-like domains 1

NM_002644 polymeric immunoglobulin receptor

NM_013281 fibronectin leucine rich transmembrane protein 3

**Gene Group 32 Enrichment Score: 0.12672212091577478**

**REFSEQ_MRNA Gene Name**

NM_153689 chromosome 2 open reading frame 69

NM_213609 family with sequence similarity 19 (chemokine (C-C motif)-like), member A1

NM_012098 angiopoietin-like 2

NM_178565 R-spondin 2 homolog (Xenopus laevis)

NM_138771 coiled-coil domain containing 126

NM_014421 dickkopf homolog 2 (Xenopus laevis)

**Healthy gd20 Endometrium > Non-Pregnant Endometrium**

**Gene Functional Classification Result**

**Gene Group 1 Enrichment Score: 4.340373898172667**

**REFSEQ_MRNA Gene Name**

NM_000090 collagen, type III, alpha 1

NM_001851 collagen, type IX, alpha 1

NM_000094 collagen, type VII, alpha 1

NM_000091 collagen, type IV, alpha 3 (Goodpasture antigen)

NM_001846 collagen, type IV, alpha 2

NM_001845 collagen, type IV, alpha 1

NM_000393 collagen, type V, alpha 2

NM_001844 collagen, type II, alpha 1

NM_001858 collagen, type XIX, alpha 1

NM_000092 collagen, type IV, alpha 4

NM_004369 collagen, type VI, alpha 3

NM_001854 collagen, type XI, alpha 1

NM_000089 collagen, type I, alpha 2

**Gene Group 2 Enrichment Score: 3.2048444134982588**

**REFSEQ_MRNA Gene Name**

NM_173560 regulatory factor X, 6

NM_006106 Yes-associated protein 1, 65kDa

NM_018416 forkhead box J2

NM_012081 elongation factor, RNA polymerase II, 2

NM_006940, NM_178010 SRY (sex determining region Y)-box 5

NM_004449 v-ets erythroblastosis virus E26 oncogene homolog (avian)

NM_012082 zinc finger protein, multitype 2

NM_001621 aryl hydrocarbon receptor

NM_003205 transcription factor 12

NM_005316 general transcription factor IIH, polypeptide 1, 62kDa

NM_003150 signal transducer and activator of transcription 3 (acute-phase response factor)

NM_012257 HMG-box transcription factor 1

NM_003222 transcription factor AP-2 gamma (activating enhancer binding protein 2 gamma)

NM_002657 pleiomorphic adenoma gene-like 2; similar to pleiomorphic adenoma gene-like 2

NM_014757 mastermind-like 1 (Drosophila)

NM_000346 SRY (sex determining region Y)-box 9

NM_002015 forkhead box O1

NM_007162 transcription factor EB

NM_002158 forkhead box N2

NM_004898 clock homolog (mouse)

NM_000965 retinoic acid receptor, beta

NM_006874 E74-like factor 2 (ets domain transcription factor)

NM_152622 mesoderm induction early response 1, family member 3

NM_005225 E2F transcription factor 1

NM_005596 nuclear factor I/B

NM_003074 SWI/SNF related, matrix associated, actin dependent regulator of chromatin, subfamily c, member 1

NM_006599 nuclear factor of activated T-cells 5, tonicity-responsive

NM_006943 SRY (sex determining region Y)-box 12

NM_001964 early growth response 1

NM_033083 ELL associated factor 1

NM_020432 putative homeodomain transcription factor 2

**Gene Group 3 Enrichment Score: 2.2057497516656768**

**REFSEQ_MRNA Gene Name**

NM_001198 PR domain containing 1, with ZNF domain

NM_003651 cold shock domain protein A; cold shock domain protein A pseudogene 1

NM_002892 AT rich interactive domain 4A (RBP1-like)

NM_002971 SATB homeobox 1

NM_012082 zinc finger protein, multitype 2

**Gene Group 4 Enrichment Score: 2.1840766125983087**

**REFSEQ_MRNA Gene Name**

NM_005153 ubiquitin specific peptidase 10

NM_182765 HECT domain containing 2

NM_022824 F-box and leucine-rich repeat protein 17

NM_004232 suppressor of cytokine signaling 6

NM_020935 ubiquitin specific peptidase 37

**Gene Group 5 Enrichment Score: 2.1234442673729745**

**REFSEQ_MRNA Gene Name**

NM_017742 zinc finger, CCHC domain containing 2

NM_001198 PR domain containing 1, with ZNF domain

NM_006940, NM_178010 SRY (sex determining region Y)-box 5

NM_012082 zinc finger protein, multitype 2

NM_002655 pleiomorphic adenoma gene 1

NM_003453 zinc finger, MYM-type 2

NM_006186 nuclear receptor subfamily 4, group A, member 2

NM_022893 B-cell CLL/lymphoma 11A (zinc finger protein)

NM_032373 polycomb group ring finger 5

NM_080764 zinc finger protein 280B

NM_001001395 LIM domain only 3 (rhombotin-like 2)

NM_016620 zinc finger protein 644

NM_005985 snail homolog 1 (Drosophila)

NM_152271 LON peptidase N-terminal domain and ring finger 1

NM_005522 homeobox A1

NM_002657 pleiomorphic adenoma gene-like 2; similar to pleiomorphic adenoma gene-like 2

NM_002202 ISL LIM homeobox 1

NM_020870 SH3 domain containing ring finger 1

NM_012421 rearranged L-myc fusion

NM_005664 makorin ring finger protein 3

NM_032268 zinc and ring finger 1

NM_015339 activity-dependent neuroprotector homeobox

NM_015435 ring finger protein 19A

NM_004926 zinc finger protein 36, C3H type-like 1

NM_032246 mex-3 homolog B (C. elegans)

NM_022898 B-cell CLL/lymphoma 11B (zinc finger protein)

NM_014615 KIAA0182

NM_203350 zinc finger, RAN-binding domain containing 2

NM_001206 Kruppel-like factor 9

NM_001964 early growth response 1

**Gene Group 6 Enrichment Score: 1.9331798784952856**

**REFSEQ_MRNA Gene Name**

NM_003794 sorting nexin 4

NM_003099 sorting nexin 1

NM_022133 sorting nexin 16

NM_014035 sorting nexin 24

**Gene Group 7 Enrichment Score: 1.266572608031177**

**REFSEQ_MRNA Gene Name**

NM_152835 PDLIM1 interacting kinase 1 like

NM_023018 NAD kinase

NM_054111 inositol hexakisphosphate kinase 3

NM_005736 ARP1 actin-related protein 1 homolog A, centractin alpha (yeast)

NM_021643 tribbles homolog 2 (Drosophila)

NM_002578 p21 protein (Cdc42/Rac)-activated kinase 3

NM_005627 serum/glucocorticoid regulated kinase 1

NM_015375 dual serine/threonine and tyrosine protein kinase

NM_080836 serine/threonine kinase 35

NM_020423 SCY1-like 3 (S. cerevisiae)

NM_001613 actin, alpha 2, smooth muscle, aorta

NM_005734 homeodomain interacting protein kinase 3

NM_001222 calcium/calmodulin-dependent protein kinase II gamma

NM_001259 cyclin-dependent kinase 6

NM_173797 PAP associated domain containing 4

NM_175854 PAN3 poly(A) specific ribonuclease subunit homolog (S. cerevisiae)

NM_170709 serum/glucocorticoid regulated kinase family, member 3

NM_005159 actin, alpha, cardiac muscle 1

NM_013233 serine threonine kinase 39 (STE20/SPS1 homolog, yeast)

**Gene Group 8 Enrichment Score: 0.9877484767068843**

**REFSEQ_MRNA Gene Name**

NM_004481 UDP-N-acetyl-alpha-D-galactosamine:polypeptide N-acetylgalactosaminyltransferase 2 (GalNAc-T2)

NM_004737 like-glycosyltransferase

NM_003654 carbohydrate (keratan sulfate Gal-6) sulfotransferase 1

NM_006699 mannosidase, alpha, class 1A, member 2

NM_004482 UDP-N-acetyl-alpha-D-galactosamine:polypeptide N-acetylgalactosaminyltransferase 3 (GalNAc-T3)

NM_014918 chondroitin sulfate synthase 1

NM_207015 N-acetylated alpha-linked acidic dipeptidase-like 2

NM_005668 ST8 alpha-N-acetyl-neuraminide alpha-2,8-sialyltransferase 4

NM_015554 glucuronic acid epimerase

NM_020438 dolichyl pyrophosphate phosphatase 1

NM_032047 UDP-GlcNAc:betaGal beta-1,3-N-acetylglucosaminyltransferase 5

**Gene Group 9 Enrichment Score: 0.9027688396739102**

**REFSEQ_MRNA Gene Name**

NM_016021 ubiquitin-conjugating enzyme E2, J1 (UBC6 homolog, yeast)

NM_182765 HECT domain containing 2

NM_003350 ubiquitin-conjugating enzyme E2 variant 2

NM_080678 ubiquitin-conjugating enzyme E2F (putative)

**Gene Group 10 Enrichment Score: 0.8712768469466113**

**REFSEQ_MRNA Gene Name**

NM_021183 RAP2C, member of RAS oncogene family

NM_004040 ras homolog gene family, member B

NM_022337 RAB38, member RAS oncogene family

NM_004165 Ras-related associated with diabetes

NM_198686 RAB15, member RAS onocogene family

NM_020673 RAB22A, member RAS oncogene family

**Gene Group 11 Enrichment Score: 0.477522741317482**

**REFSEQ_MRNA Gene Name**

NM_138390 transmembrane protein 169

NM_018907 protocadherin alpha 1; protocadherin alpha 4

NM_018908 protocadherin alpha 5

NM_017893 sema domain, immunoglobulin domain (Ig), transmembrane domain (TM) and short cytoplasmic domain, (semaphorin) 4G

NM_003043 solute carrier family 6 (neurotransmitter transporter, taurine), member 6

NM_000724 calcium channel, voltage-dependent, beta 2 subunit

NM_021945 solute carrier family 22, member 23

NM_032148 solute carrier family 41, member 2

NM_018231 solute carrier family 38, member 7

NM_004736 xenotropic and polytropic retrovirus receptor

NM_024081 proline rich Gla (G-carboxyglutamic acid) 4 (transmembrane)

NM_019849 solute carrier family 7, (neutral amino acid transporter, y+ system) member 10

NM_000232 sarcoglycan, beta (43kDa dystrophin-associated glycoprotein)

NM_152522 ADP-ribosylation-like factor 6 interacting protein 6

NM_006134 transmembrane protein 50B

NM_003692 transmembrane protein with EGF-like and two follistatin-like domains 1; chromosome 9 open reading frame 30; hypothetical LOC729538

NM_018904, NM_018898, NM_018899, NM_018901 protocadherin alpha 13; protocadherin alpha 10; protocadherin alpha subfamily C, 1; protocadherin alpha subfamily C, 2

NM_031442 transmembrane protein 47

NM_152527 solute carrier family 16, member 14 (monocarboxylic acid transporter 14)

NM_018992 potassium channel tetramerisation domain containing 5

NM_032961 protocadherin 10

NM_005302 G protein-coupled receptor 37 (endothelin receptor type B-like)

NM_003615 solute carrier family 4, sodium bicarbonate cotransporter, member 7

NM_018905 protocadherin alpha 2

NM_018976 solute carrier family 38, member 2

NM_018113 limb region 1 homolog (mouse)-like

NM_001001330 receptor accessory protein 3

NM_018593 solute carrier family 16, member 10 (aromatic amino acid transporter)

NM_207015 N-acetylated alpha-linked acidic dipeptidase-like 2

NM_003759 solute carrier family 4, sodium bicarbonate cotransporter, member 4

NM_013348 potassium inwardly-rectifying channel, subfamily J, member 14

NM_005927 microfibrillar-associated protein 3

NM_021161 potassium channel, subfamily K, member 10

NM_005329 hyaluronan synthase 3

NM_018911, NM_018909 protocadherin alpha 8; protocadherin alpha 6

NM_052886 mal, T-cell differentiation protein 2

NM_020179 hypothetical LOC728675; chromosome 11 open reading frame 75

NM_018906 protocadherin alpha 3

NM_001002762 DnaJ (Hsp40) homolog, subfamily B, member 12

NM_012329 monocyte to macrophage differentiation-associated

NM_173683 XK, Kell blood group complex subunit-related family, member 6

NM_032012 chromosome 9 open reading frame 5

NM_138391 transmembrane protein 183A; transmembrane protein 183B

**Non-Pregnant Endometrium > Arresting gd20 Endometrium**

**Gene Functional Classification Result**

**Gene Group 1 Enrichment Score: 8.300876965554966**

**REFSEQ_MRNA Gene Name**

NM_014552 grainyhead-like 1 (Drosophila)

NM_152622 mesoderm induction early response 1, family member 3

NM_016374 AT rich interactive domain 4B (RBP1-like)

NM_015608 chromosome 10 open reading frame 137

NM_181782 nuclear receptor coactivator 7

NM_030759 nuclear receptor binding factor 2

NM_032352 breast cancer metastasis-suppressor 1-like

NM_006465 AT rich interactive domain 3B (BRIGHT-like)

NM_194282 lin-54 homolog (C. elegans)

**Gene Group 2 Enrichment Score: 3.942253284065122**

**REFSEQ_MRNA Gene Name**

NM_005599 nescient helix loop helix 2

NM_005924 mesenchyme homeobox 2

NM_001002909 G patch domain containing 8

NM_003822 nuclear receptor subfamily 5, group A, member 2

NM_001001890 runt-related transcription factor 1

NM_001009960, NM_138494 zinc finger protein 655

NM_030661 homeobox A3

NM_021809 TGFB-induced factor homeobox 2

NM_014819 praja ring finger 2

NM_199072 MyoD family inhibitor domain containing

NM_002912 REV3-like, catalytic subunit of DNA polymerase zeta (yeast)

NM_021620 PR domain containing 13

NM_021728 orthodenticle homeobox 2

NM_033114 zinc finger CCHC-type and RNA binding motif 1

NM_003111 Sp3 transcription factor

NM_003430 zinc finger protein 91

NM_152437 zinc finger protein 664

NM_183238 similar to ZNF605 protein

NM_012308 lysine (K)-specific demethylase 2A

NM_014910 zinc finger protein 507

NM_003216 thyrotrophic embryonic factor

NM_015995 Kruppel-like factor 13

NM_057175 NMDA receptor regulated 1

NM_032440 ligand dependent nuclear receptor corepressor

NM_005180 BMI1 polycomb ring finger oncogene

NM_005234 nuclear receptor subfamily 2, group F, member 6

NM_004229 mediator complex subunit 14

NM_014795 zinc finger E-box binding homeobox 2

NM_203394 E2F transcription factor 7

NM_005230 ELK3, ETS-domain protein (SRF accessory protein 2)

NM_004973 jumonji, AT rich interactive domain 2

NM_022893 B-cell CLL/lymphoma 11A (zinc finger protein)

NM_022728 neurogenic differentiation 6

NM_001011666, NM_004904, NM_182898, NM_182899 cAMP responsive element binding protein 5

NM_058166, NM_001003818 TRIM6-TRIM34 readthrough transcript; tripartite motif-containing 6; tripartite motif-containing 34

NM_003107 SRY (sex determining region Y)-box 4

NM_002382 MYC associated factor X

NM_001806 CCAAT/enhancer binding protein (C/EBP), gamma

NM_025188 tripartite motif-containing 45

NM_006164 nuclear factor (erythroid-derived 2)-like 2

NM_018433 lysine (K)-specific demethylase 3A

NM_006352 zinc finger protein 238

NM_001001419 SMAD family member 5

NM_004992 methyl CpG binding protein 2 (Rett syndrome)

NM_018263 additional sex combs like 2 (Drosophila)

NM_004293 guanine deaminase

NM_005667 vacuolar protein sorting 24 homolog (S. cerevisiae); ring finger protein 103

NM_001001992, NM_006447, NM_001032410 ubiquitin specific peptidase 16

NM_144684 zinc finger protein 480

NM_000176 nuclear receptor subfamily 3, group C, member 1 (glucocorticoid receptor)

NM_017769 G2/M-phase specific E3 ubiquitin ligase

NM_002198 interferon regulatory factor 1

NM_012330 MYST histone acetyltransferase (monocytic leukemia) 4

NM_001039649 zinc finger, MYM-type 5

NM_015153 PHD finger protein 3

NM_002938 ring finger protein 4; hypothetical LOC644006

NM_014682 suppression of tumorigenicity 18 (breast carcinoma) (zinc finger protein)

NM_006734 human immunodeficiency virus type I enhancer binding protein 2

NM_015534 zinc finger, ZZ-type containing 3

NM_000248 microphthalmia-associated transcription factor

NM_144726 ring finger protein 145

NM_033013 nuclear receptor subfamily 1, group I, member 2

NM_022781 ring finger protein 38

NM_001438 estrogen-related receptor gamma

NM_015481 zinc finger protein 385A

NM_015246 mahogunin, ring finger 1

NM_004379 cAMP responsive element binding protein 1

NM_006599 nuclear factor of activated T-cells 5, tonicity-responsive

NM_012234 RING1 and YY1 binding protein

NM_006022 TSC22 domain family, member 1

NM_005461 v-maf musculoaponeurotic fibrosarcoma oncogene homolog B (avian)

NM_001452 forkhead box F2

NM_015057 MYC binding protein 2

NM_002166 inhibitor of DNA binding 2, dominant negative helix-loop-helix protein

NM_019083 coiled-coil domain containing 76

NM_002460 interferon regulatory factor 4

NM_006458 tripartite motif-containing 3

NM_030751 zinc finger E-box binding homeobox 1

NM_153607 chromosome 5 open reading frame 41

NM_138473 Sp1 transcription factor

NM_002167 inhibitor of DNA binding 3, dominant negative helix-loop-helix protein

NM_022658 homeobox C8

NM_014491 forkhead box P2

NM_014240 LIM domains containing 1

NM_018419 SRY (sex determining region Y)-box 18

NM_005955 metal-regulatory transcription factor 1

NM_007249 Kruppel-like factor 12

NM_018951 homeobox A10

NM_021961 TEA domain family member 1 (SV40 transcriptional enhancer factor)

NM_053002 mediator complex subunit 12-like

NM_015271 tripartite motif-containing 2

NM_000901 nuclear receptor subfamily 3, group C, member 2

NM_004235 Kruppel-like factor 4 (gut)

NM_006526 zinc finger protein 217

NM_013450 bromodomain adjacent to zinc finger domain, 2B

NM_019102 homeobox A5

NM_017519 AT rich interactive domain 1B (SWI1-like)

NM_003112 Sp4 transcription factor

NM_000125 estrogen receptor 1

NM_005316 general transcription factor IIH, polypeptide 1, 62kDa

NM_001013691 zinc finger protein 833

NM_003721 regulatory factor X-associated ankyrin-containing protein

NM_004241 jumonji domain containing 1C

NM_002500 neurogenic differentiation 1

NM_013316 CCR4-NOT transcription complex, subunit 4

NM_181353 inhibitor of DNA binding 1, dominant negative helix-loop-helix protein

NM_024672 THAP domain containing 9

NM_032329 inhibitor of growth family, member 5

NM_152787 mitogen-activated protein kinase kinase kinase 7 interacting protein 3

NM_017656 zinc finger protein 562

NM_024674 lin-28 homolog (C. elegans)

NM_001012756 zinc finger protein 260

NM_004234 zinc finger protein 235

NM_020310 MAX binding protein

**Gene Group 3 Enrichment Score: 3.3835892087915025**

**REFSEQ_MRNA Gene Name**

NM_019027 RNA binding motif protein 47

NM_016090 RNA binding motif protein 7

NM_018211 ribonucleoprotein, PTB-binding 2

NM_007007 cleavage and polyadenylation specific factor 6, 68kDa

NM_080832 poly(A) binding protein, cytoplasmic 5

NM_014912 cytoplasmic polyadenylation element binding protein 3

**Gene Group 4 Enrichment Score: 3.136333597737212**

**REFSEQ_MRNA Gene Name**

NM_052851, NM_178006, NM_178007 StAR-related lipid transfer (START) domain containing 13

NM_032900 Rho GTPase activating protein 19

NM_014859 Rho-type GTPase-activating protein RICH2

NM_018948 ERBB receptor feedback inhibitor 1

NM_020824 Rho GTPase activating protein 21

NM_016603 family with sequence similarity 13, member B

NM_018287 Rho GTPase activating protein 12

**Gene Group 5 Enrichment Score: 2.9426038571108863**

**REFSEQ_MRNA Gene Name**

NM_024039 MIS12, MIND kinetochore complex component, homolog (S. pombe)

NM_006265 RAD21 homolog (S. pombe)

NM_005862 stromal antigen 1

NM_015032 PDS5, regulator of cohesion maintenance, homolog B (S. cerevisiae)

NM_152510 HORMA domain containing 2

NM_004060 cyclin G1

NM_001042549, NM_015471 NSL1, MIND kinetochore complex component, homolog (S. cerevisiae)

NM_002956 CAP-GLY domain containing linker protein 1

**Gene Group 6 Enrichment Score: 2.8835640280126933**

**REFSEQ_MRNA Gene Name**

NM_145872 ankyrin repeat and SOCS box-containing 4

NM_013396 ubiquitin specific peptidase 25

NM_004656 BRCA1 associated protein-1 (ubiquitin carboxy-terminal hydrolase)

NM_015017 ubiquitin specific peptidase 33

NM_022832 ubiquitin specific peptidase 46

NM_001001664 speckle-type POZ protein-like

NM_015626 WD repeat and SOCS box-containing 1

NM_032236 ubiquitin specific peptidase 48

NM_004232 suppressor of cytokine signaling 6

NM_015176 F-box protein 28

NM_015246 mahogunin, ring finger 1

**Gene Group 7 Enrichment Score: 2.7584002492725923**

**REFSEQ_MRNA Gene Name**

NM_001627 hypothetical protein LOC100133690; activated leukocyte cell adhesion molecule

NM_175607 contactin 4

NM_003872 neuropilin 2

NM_003873 neuropilin 1

NM_020796 sema domain, transmembrane domain (TM), and cytoplasmic domain, (semaphorin) 6A

**Gene Group 8 Enrichment Score: 2.5505777708051833**

**REFSEQ_MRNA Gene Name**

NM_003390 WEE1 homolog (S. pombe)

NM_032430 BR serine/threonine kinase 1

NM_007170 testis-specific kinase 2

NM_173354 salt-inducible kinase 1

NM_004438 EPH receptor A4

NM_014683 unc-51-like kinase 2 (C. elegans)

NM_001274 CHK1 checkpoint homolog (S. pombe)

NM_003010 mitogen-activated protein kinase kinase 4

NM_006609 mitogen-activated protein kinase kinase kinase 2

NM_021133 ribonuclease L (2',5'-oligoisoadenylate synthetase-dependent)

NM_005400 protein kinase C, epsilon

NM_198465 Nik related kinase

NM_014397 NIMA (never in mitosis gene a)-related kinase 6

NM_002314 LIM domain kinase 1

NM_006251 protein kinase, AMP-activated, alpha 1 catalytic subunit

NM_001259 cyclin-dependent kinase 6

NM_005813 protein kinase D3

NM_001204 bone morphogenetic protein receptor, type II (serine/threonine kinase)

NM_020778 alpha-kinase 3

NM_017719 SNF related kinase

NM_005433 v-yes-1 Yamaguchi sarcoma viral oncogene homolog 1

NM_175854 PAN3 poly(A) specific ribonuclease subunit homolog (S. cerevisiae)

NM_015076 cell division cycle 2-like 6 (CDK8-like)

NM_002031 fyn-related kinase

NM_006852 tousled-like kinase 2

NM_001895 casein kinase 2, alpha 1 polypeptide pseudogene; casein kinase 2, alpha 1 polypeptide

NM_016231 nemo-like kinase

**Gene Group 9 Enrichment Score: 2.4420202392055614**

**REFSEQ_MRNA Gene Name**

NM_004728 DEAD (Asp-Glu-Ala-Asp) box polypeptide 21

NM_025134 chromodomain helicase DNA binding protein 9

NM_021931 DEAH (Asp-Glu-Ala-His) box polypeptide 35

NM_000489 alpha thalassemia/mental retardation syndrome X-linked (RAD54 homolog, S. cerevisiae)

NM_003972 BTAF1 RNA polymerase II, B-TFIID transcription factor-associated, 170kDa (Mot1 homolog, S. cerevisiae)

NM_017553 INO80 homolog (S. cerevisiae)

NM_004396 DEAD (Asp-Glu-Ala-Asp) box polypeptide 5

**Gene Group 10 Enrichment Score: 2.1944046467221536**

**REFSEQ_MRNA Gene Name**

NM_018211 ribonucleoprotein, PTB-binding 2

NM_006924 splicing factor, arginine/serine-rich 1

NM_001031684 splicing factor, arginine/serine-rich 7, 35kDa

NM_007007 cleavage and polyadenylation specific factor 6, 68kDa

NM_033114 zinc finger CCHC-type and RNA binding motif 1

NM_004768 splicing factor, arginine/serine-rich 11

NM_005105 RNA binding motif protein 8A

NM_145893 ataxin 2-binding protein 1

**Gene Group 11 Enrichment Score: 1.9874318323088664**

**REFSEQ_MRNA Gene Name**

NM_014819 praja ring finger 2

NM_199332 homer homolog 2 (Drosophila)

NM_001364 discs, large homolog 2 (Drosophila)

NM_004745 discs, large (Drosophila) homolog-associated protein 2

**Gene Group 12 Enrichment Score: 1.4182825949331825**

**REFSEQ_MRNA Gene Name**

NM_015263 Dmx-like 2

NM_005509 Dmx-like 1

NM_015626 WD repeat and SOCS box-containing 1

NM_014969 WD repeat domain 47

NM_020245, NM_001007466 tubby like protein 4

NM_017974 ATG16 autophagy related 16-like 1 (S. cerevisiae)

NM_018315 F-box and WD repeat domain containing 7

NM_015726 WD repeat domain 42A

**Gene Group 13 Enrichment Score: 1.3351890482796611**

**REFSEQ_MRNA Gene Name**

NM_021183 RAP2C, member of RAS oncogene family

NM_016277, NM_183227 RAB23, member RAS oncogene family

NM_016544 DnaJ (Hsp40) homolog, subfamily C, member 27

NM_004162 RAB5A, member RAS oncogene family

NM_014488 RAB30, member RAS oncogene family

NM_031934 RAB34, member RAS oncogene family

NM_019067 guanine nucleotide binding protein-like 3 (nucleolar)-like

NM_014170, NM_138485 GTP-binding protein 8 (putative)

**Gene Group 14 Enrichment Score: 1.1720804441320964**

**REFSEQ_MRNA Gene Name**

NM_004827 ATP-binding cassette, sub-family G (WHITE), member 2

NM_004299 ATP-binding cassette, sub-family B (MDR/TAP), member 7

NM_005164 ATP-binding cassette, sub-family D (ALD), member 2

NM_020453 ATPase, class V, type 10D

NM_012089 ATP-binding cassette, sub-family B (MDR/TAP), member 10

NM_001995 acyl-CoA synthetase long-chain family member 1

NM_004458 acyl-CoA synthetase long-chain family member 4

NM_033540 mitofusin 1

**Gene Group 15 Enrichment Score: 0.8570167308939589**

**REFSEQ_MRNA Gene Name**

NM_001017971 ATPase, H+ transporting, lysosomal accessory protein 1-like

NM_001693 ATPase, H+ transporting, lysosomal 56/58kDa, V1 subunit B2

NM_020453 ATPase, class V, type 10D

NM_130841 ATPase, H+ transporting, lysosomal V0 subunit a4

**Gene Group 16 Enrichment Score: 0.7774913769121722**

**REFSEQ_MRNA Gene Name**

NM_152996 ST6 (alpha-N-acetyl-neuraminyl-2,3-beta-galactosyl-1,3)-N-acetylgalactosaminide alpha-2,6-sialyltransferase 3

NM_031434 transmembrane and ubiquitin-like domain containing 1

NM_004776 UDP-Gal:betaGlcNAc beta 1,4- galactosyltransferase, polypeptide 5

NM_005810 killer cell lectin-like receptor subfamily G, member 1

NM_032256 transmembrane protein 117

NM_016388 T cell receptor associated transmembrane adaptor 1

NM_017786 Golgi-localized protein

NM_014702 KIAA0408; chromosome 6 open reading frame 174

NM_001004439 integrin, alpha 11

NM_052885 solute carrier family 2 (facilitated glucose transporter), member 13

NM_003654 carbohydrate (keratan sulfate Gal-6) sulfotransferase 1

NM_015008 transmembrane and coiled-coil domain family 1

NM_182511 cerebellin 2 precursor

NM_004616 tetraspanin 8

NM_002205 integrin, alpha 5 (fibronectin receptor, alpha polypeptide)

NM_198337 insulin induced gene 1

NM_138340 abhydrolase domain containing 3

NM_021978 suppression of tumorigenicity 14 (colon carcinoma)

NM_024913 chromosome 7 open reading frame 58

NM_080927 discoidin, CUB and LCCL domain containing 2

NM_001002257 lysocardiolipin acyltransferase 1

NM_152666 phospholipase D family, member 5

NM_019556 motile sperm domain containing 1

NM_003782 UDP-Gal:betaGlcNAc beta 1,3-galactosyltransferase, polypeptide 4

NM_001004067 NODAL modulator 3; NODAL modulator 1; NODAL modulator 2

NM_024056 transmembrane protein 106C

NM_001076 UDP glucuronosyltransferase 2 family, polypeptide B15

NM_022912 receptor accessory protein 1

NM_001079802, NM_006731 fukutin

NM_005779 lipoma HMGIC fusion partner-like 2

NM_080546 solute carrier family 44, member 1

NM_207015 N-acetylated alpha-linked acidic dipeptidase-like 2

NM_003779 UDP-Gal:betaGlcNAc beta 1,4- galactosyltransferase, polypeptide 3

NM_018375 solute carrier family 39 (zinc transporter), member 9

NM_001781 CD69 molecule

NM_018930 protocadherin beta 10; protocadherin beta 9

NM_002210 integrin, alpha V (vitronectin receptor, alpha polypeptide, antigen CD51)

NM_182527 calcium binding protein 7

NM_015497 transmembrane protein 87A

NM_022484 transmembrane protein 168

NM_014283 chromosome 1 open reading frame 9

NM_181644 major facilitator superfamily domain containing 4

NM_134431 solute carrier organic anion transporter family, member 1A2

NM_181836 transmembrane emp24 protein transport domain containing 7; toll-like receptor adaptor molecule 2

NM_006134 transmembrane protein 50B

NM_015879 ST8 alpha-N-acetyl-neuraminide alpha-2,8-sialyltransferase 3

NM_144599 non imprinted in Prader-Willi/Angelman syndrome 1

NM_024769 adipocyte-specific adhesion molecule

NM_005907 mannosidase, alpha, class 1A, member 1

NM_138728, NM_138727, NM_017744 suppression of tumorigenicity 7 like

NM_003498 stannin

NM_024334 transmembrane protein 43

NM_016548 golgi membrane protein 1

NM_020182 prostate transmembrane protein, androgen induced 1

NM_024569 myelin protein zero-like 1

NM_012329 monocyte to macrophage differentiation-associated

NM_005776 cornichon homolog (Drosophila)

NM_002207 integrin, alpha 9

NM_153261 transmembrane protein 188; similar to TMEM188 protein

NM_020123 transmembrane 9 superfamily member 3

NM_002099 glycophorin A (MNS blood group)

NM_001795 cadherin 5, type 2 (vascular endothelium)

NM_000958 prostaglandin E receptor 4 (subtype EP4)

NM_006691 lymphatic vessel endothelial hyaluronan receptor 1

NM_001080505 shisa homolog 3 (Xenopus laevis)

NM_017423 UDP-N-acetyl-alpha-D-galactosamine:polypeptide N-acetylgalactosaminyltransferase 7 (GalNAc-T7)

NM_002644 polymeric immunoglobulin receptor

NM_001003674 chromosome 18 open reading frame 1

NM_022736 major facilitator superfamily domain containing 1

NM_006581 fucosyltransferase 9 (alpha (1,3) fucosyltransferase)

NM_180989 G protein-coupled receptor 180

NM_153365 transmembrane anterior posterior transformation 1

NM_153711 family with sequence similarity 26, member E

NM_004028, NM_001650 aquaporin 4

NM_032039 integrin alpha FG-GAP repeat containing 3

NM_002033 fucosyltransferase 4 (alpha (1,3) fucosyltransferase, myeloid-specific)

NM_053039 UDP glucuronosyltransferase 2 family, polypeptide B28

NM_022373 HERPUD family member 2

NM_033428 chromosome 9 open reading frame 123

NM_002203 integrin, alpha 2 (CD49B, alpha 2 subunit of VLA-2 receptor)

NM_178454 DNA-damage regulated autophagy modulator 2

NM_005277 glycoprotein M6A

NM_020644 TMEM9 domain family, member B

NM_016072 golgi transport 1 homolog B (S. cerevisiae)

NM_013281 fibronectin leucine rich transmembrane protein 3

NM_013381 thyrotropin-releasing hormone degrading enzyme

NM_032312 Yip1 domain family, member 4

NM_014373 G protein-coupled receptor 160

NM_001077 UDP glucuronosyltransferase 2 family, polypeptide B17

NM_024312 N-acetylglucosamine-1-phosphate transferase, alpha and beta subunits

NM_025250 tweety homolog 3 (Drosophila)

NM_024795 transmembrane 4 L six family member 20

NM_032973 protocadherin 11 Y-linked

NM_002998 syndecan 2

NM_002372 mannosidase, alpha, class 2A, member 1

NM_138771 coiled-coil domain containing 126

NM_001004360 dipeptidyl-peptidase 10

NM_017911 family with sequence similarity 118, member A

NM_198549 family with sequence similarity 73, member A

**Gene Group 17 Enrichment Score: 0.7577378518544915**

**REFSEQ_MRNA Gene Name**

NM_020751 component of oligomeric golgi complex 6

NM_152628 sorting nexin 31

NM_022459 exportin 4

NM_030918 sorting nexin family member 27

**Gene Group 18 Enrichment Score: 0.5468730991930171**

**REFSEQ_MRNA Gene Name**

NM_018375 solute carrier family 39 (zinc transporter), member 9

NM_020708 solute carrier family 12 (potassium-chloride transporter), member 5

NM_025250 tweety homolog 3 (Drosophila)

NM_020689 solute carrier family 24 (sodium/potassium/calcium exchanger), member 3

NM_133478 solute carrier family 4, sodium bicarbonate cotransporter, member 5

NM_139177 solute carrier family 39 (metal ion transporter), member 11

NM_001042537, NM_006359 solute carrier family 9 (sodium/hydrogen exchanger), member 6

NM_022058 solute carrier family 4, sodium bicarbonate transporter, member 10

**Gene Group 19 Enrichment Score: 0.4228560287283282**

**REFSEQ_MRNA Gene Name**

NM_024769 adipocyte-specific adhesion molecule

NM_138440 vasorin

NM_012428 neuroplastin

NM_024569 myelin protein zero-like 1

NM_018490 leucine-rich repeat-containing G protein-coupled receptor 4

NM_052910 SLIT and NTRK-like family, member 1

NM_002644 polymeric immunoglobulin receptor

NM_002099 glycophorin A (MNS blood group)

NM_015541 leucine-rich repeats and immunoglobulin-like domains 1

NM_013281 fibronectin leucine rich transmembrane protein 3

NM_001627 hypothetical protein LOC100133690; activated leukocyte cell adhesion molecule

NM_001393 extracellular matrix protein 2, female organ and adipocyte specific

NM_002998 syndecan 2

NM_006378 sema domain, immunoglobulin domain (Ig), transmembrane domain (TM) and short cytoplasmic domain, (semaphorin) 4D

**Gene Group 20 Enrichment Score: 0.37696928072297725**

**REFSEQ_MRNA Gene Name**

NM_020116 follistatin-like 5

NM_001219 calumenin

NM_182527 calcium binding protein 7

NM_012198 grancalcin, EF-hand calcium binding protein

NM_022138 SPARC related modular calcium binding 2

**Gene Group 21 Enrichment Score: 0.32047137831349193**

**REFSEQ_MRNA Gene Name**

NM_032505 kelch repeat and BTB (POZ) domain containing 8

NM_014962 BTB (POZ) domain containing 3

NM_152433, NM_198439 kelch repeat and BTB (POZ) domain containing 3

NM_025010 kelch-like 18 (Drosophila)

**Arresting gd20 Endometrium > Non-Pregnant Endometrium**

**Gene Functional Classification Result**

**Gene Group 1 Enrichment Score: 5.855980755617018**

**REFSEQ_MRNA Gene Name**

NM_000090 collagen, type III, alpha 1

NM_000094 collagen, type VII, alpha 1

NM_001851 collagen, type IX, alpha 1

NM_015719 collagen, type V, alpha 3

NM_000091 collagen, type IV, alpha 3 (Goodpasture antigen)

NM_001846 collagen, type IV, alpha 2

NM_001845 collagen, type IV, alpha 1

NM_000088 collagen, type I, alpha 1

NM_000393 collagen, type V, alpha 2

NM_001844 collagen, type II, alpha 1

NM_001858 collagen, type XIX, alpha 1

NM_000092 collagen, type IV, alpha 4

NM_004369 collagen, type VI, alpha 3

NM_001855 collagen, type XV, alpha 1

NM_001854 collagen, type XI, alpha 1

NM_000089 collagen, type I, alpha 2

**Gene Group 2 Enrichment Score: 3.130984711596011**

**REFSEQ_MRNA Gene Name**

NM_173560 regulatory factor X, 6

NM_005595 nuclear factor I/A

NM_006943 SRY (sex determining region Y)-box 12

NM_080764 zinc finger protein 280B

NM_002657 pleiomorphic adenoma gene-like 2; similar to pleiomorphic adenoma gene-like 2

NM_003074 SWI/SNF related, matrix associated, actin dependent regulator of chromatin, subfamily c, member 1

NM_012082 zinc finger protein, multitype 2

NM_015435 ring finger protein 19A

NM_001621 aryl hydrocarbon receptor

NM_015339 activity-dependent neuroprotector homeobox

NM_020870 SH3 domain containing ring finger 1

NM_003575 zinc finger protein 282

NM_022462 hypoxia inducible factor 3, alpha subunit

NM_032373 polycomb group ring finger 5

NM_000965 retinoic acid receptor, beta

NM_022817 period homolog 2 (Drosophila)

NM_012421 rearranged L-myc fusion

NM_002158 forkhead box N2

NM_003150 signal transducer and activator of transcription 3 (acute-phase response factor)

NM_020432 putative homeodomain transcription factor 2

NM_022898 B-cell CLL/lymphoma 11B (zinc finger protein)

NM_001964 early growth response 1

NM_032246 mex-3 homolog B (C. elegans)

NM_016620 zinc finger protein 644

NM_152622 mesoderm induction early response 1, family member 3

NM_033083 ELL associated factor 1

NM_005985 snail homolog 1 (Drosophila)

NM_004926 zinc finger protein 36, C3H type-like 1

NM_002015 forkhead box O1

NM_030625 tet oncogene 1

NM_006599 nuclear factor of activated T-cells 5, tonicity-responsive

NM_000346 SRY (sex determining region Y)-box 9

NM_003222 transcription factor AP-2 gamma (activating enhancer binding protein 2 gamma)

NM_005664 makorin ring finger protein 3

NM_005596 nuclear factor I/B

NM_014615 KIAA0182

NM_004898 clock homolog (mouse)

NM_014757 mastermind-like 1 (Drosophila)

NM_005316 general transcription factor IIH, polypeptide 1, 62kDa

NM_001198 PR domain containing 1, with ZNF domain

NM_005225 E2F transcription factor 1

NM_002655 pleiomorphic adenoma gene 1

NM_012081 elongation factor, RNA polymerase II, 2

NM_032268 zinc and ring finger 1

NM_006940, NM_178010 SRY (sex determining region Y)-box 5

NM_053002 mediator complex subunit 12-like

NM_203350 zinc finger, RAN-binding domain containing 2

NM_006874 E74-like factor 2 (ets domain transcription factor)

NM_004449 v-ets erythroblastosis virus E26 oncogene homolog (avian)

NM_018416 forkhead box J2

NM_003453 zinc finger, MYM-type 2

NM_017742 zinc finger, CCHC domain containing 2

NM_012257 HMG-box transcription factor 1

NM_152493 zinc finger protein 362

NM_022893 B-cell CLL/lymphoma 11A (zinc finger protein)

NM_015457 zinc finger, DHHC-type containing 5

NM_001001395 LIM domain only 3 (rhombotin-like 2)

NM_007162 transcription factor EB

NM_003205 transcription factor 12

NM_006106 Yes-associated protein 1, 65kDa

NM_001206 Kruppel-like factor 9

**Gene Group 3 Enrichment Score: 2.598349013091264**

**REFSEQ_MRNA Gene Name**

NM_002657 pleiomorphic adenoma gene-like 2; similar to pleiomorphic adenoma gene-like 2

NM_005522 homeobox A1

NM_002202 ISL LIM homeobox 1

NM_006186 nuclear receptor subfamily 4, group A, member 2

NM_022898 B-cell CLL/lymphoma 11B (zinc finger protein)

**Gene Group 4 Enrichment Score: 2.5950534161494807**

**REFSEQ_MRNA Gene Name**

NM_001198 PR domain containing 1, with ZNF domain

NM_002892 AT rich interactive domain 4A (RBP1-like)

NM_003651 cold shock domain protein A; cold shock domain protein A pseudogene 1

NM_002971 SATB homeobox 1

NM_012082 zinc finger protein, multitype 2

**Gene Group 5 Enrichment Score: 1.8306248839831323**

**REFSEQ_MRNA Gene Name**

NM_182765 HECT domain containing 2

NM_022824 F-box and leucine-rich repeat protein 17

NM_004232 suppressor of cytokine signaling 6

NM_020935 ubiquitin specific peptidase 37

**Gene Group 6 Enrichment Score: 1.8107143900968026**

**REFSEQ_MRNA Gene Name**

NM_003794 sorting nexin 4

NM_003099 sorting nexin 1

NM_022133 sorting nexin 16

NM_014035 sorting nexin 24

**Gene Group 7 Enrichment Score: 0.9615110779022467**

**REFSEQ_MRNA Gene Name**

NM_152835 PDLIM1 interacting kinase 1 like

NM_023018 NAD kinase

NM_054111 inositol hexakisphosphate kinase 3

NM_005736 ARP1 actin-related protein 1 homolog A, centractin alpha (yeast)

NM_021643 tribbles homolog 2 (Drosophila)

NM_005627 serum/glucocorticoid regulated kinase 1

NM_015375 dual serine/threonine and tyrosine protein kinase

NM_080836 serine/threonine kinase 35

NM_020423 SCY1-like 3 (S. cerevisiae)

NM_001613 actin, alpha 2, smooth muscle, aorta

NM_005734 homeodomain interacting protein kinase 3

NM_001259 cyclin-dependent kinase 6

NM_001222 calcium/calmodulin-dependent protein kinase II gamma

NM_173797 PAP associated domain containing 4

NM_175854 PAN3 poly(A) specific ribonuclease subunit homolog (S. cerevisiae)

NM_170709 serum/glucocorticoid regulated kinase family, member 3

NM_005159 actin, alpha, cardiac muscle 1

NM_013233 serine threonine kinase 39 (STE20/SPS1 homolog, yeast)

**Gene Group 8 Enrichment Score: 0.8868013748578355**

**REFSEQ_MRNA Gene Name**

NM_004481 UDP-N-acetyl-alpha-D-galactosamine:polypeptide N-acetylgalactosaminyltransferase 2 (GalNAc-T2)

NM_004737 like-glycosyltransferase

NM_003654 carbohydrate (keratan sulfate Gal-6) sulfotransferase 1

NM_006699 mannosidase, alpha, class 1A, member 2

NM_004482 UDP-N-acetyl-alpha-D-galactosamine:polypeptide N-acetylgalactosaminyltransferase 3 (GalNAc-T3)

NM_014918 chondroitin sulfate synthase 1

NM_207015 N-acetylated alpha-linked acidic dipeptidase-like 2

NM_015554 glucuronic acid epimerase

NM_005668 ST8 alpha-N-acetyl-neuraminide alpha-2,8-sialyltransferase 4

NM_020438 dolichyl pyrophosphate phosphatase 1

NM_032047 UDP-GlcNAc:betaGal beta-1,3-N-acetylglucosaminyltransferase 5

**Gene Group 9 Enrichment Score: 0.7298123686647737**

**REFSEQ_MRNA Gene Name**

NM_021183 RAP2C, member of RAS oncogene family

NM_004040 ras homolog gene family, member B

NM_022337 RAB38, member RAS oncogene family

NM_004165 Ras-related associated with diabetes

NM_198686 RAB15, member RAS onocogene family

NM_020673 RAB22A, member RAS oncogene family

**Gene Group 10 Enrichment Score: 0.597501019586585**

**REFSEQ_MRNA Gene Name**

NM_016021 ubiquitin-conjugating enzyme E2, J1 (UBC6 homolog, yeast)

NM_182765 HECT domain containing 2

NM_003350 ubiquitin-conjugating enzyme E2 variant 2

NM_080678 ubiquitin-conjugating enzyme E2F (putative)

**Gene Group 11 Enrichment Score: 0.4233498956665482**

**REFSEQ_MRNA Gene Name**

NM_138390 transmembrane protein 169

NM_018908 protocadherin alpha 5

NM_018907, NM_018900 protocadherin alpha 1; protocadherin alpha 4

NM_003043 solute carrier family 6 (neurotransmitter transporter, taurine), member 6

NM_000724 calcium channel, voltage-dependent, beta 2 subunit

NM_021945 solute carrier family 22, member 23

NM_004796 neurexin 3

NM_032148 solute carrier family 41, member 2

NM_018231 solute carrier family 38, member 7

NM_004736 xenotropic and polytropic retrovirus receptor

NM_024081 proline rich Gla (G-carboxyglutamic acid) 4 (transmembrane)

NM_019849 solute carrier family 7, (neutral amino acid transporter, y+ system) member 10

NM_000232 sarcoglycan, beta (43kDa dystrophin-associated glycoprotein)

NM_152522 ADP-ribosylation-like factor 6 interacting protein 6

NM_003692 transmembrane protein with EGF-like and two follistatin-like domains 1; chromosome 9 open reading frame 30; hypothetical LOC729538

NM_018904, NM_018899, NM_018898, NM_018901 protocadherin alpha 13; protocadherin alpha 10; protocadherin alpha subfamily C, 1; protocadherin alpha subfamily C, 2

NM_031442 transmembrane protein 47

NM_152527 solute carrier family 16, member 14 (monocarboxylic acid transporter 14)

NM_018992 potassium channel tetramerisation domain containing 5

NM_032961 protocadherin 10

NM_005302 G protein-coupled receptor 37 (endothelin receptor type B-like)

NM_018903 protocadherin alpha 12

NM_003615 solute carrier family 4, sodium bicarbonate cotransporter, member 7

NM_018976 solute carrier family 38, member 2

NM_018905 protocadherin alpha 2

NM_018113 limb region 1 homolog (mouse)-like

NM_001001330 receptor accessory protein 3

NM_018593 solute carrier family 16, member 10 (aromatic amino acid transporter)

NM_207015 N-acetylated alpha-linked acidic dipeptidase-like 2

NM_005927 microfibrillar-associated protein 3

NM_013348 potassium inwardly-rectifying channel, subfamily J, member 14

NM_021161 potassium channel, subfamily K, member 10

NM_018909, NM_018911 protocadherin alpha 8; protocadherin alpha 6

NM_005329 hyaluronan synthase 3

NM_052886 mal, T-cell differentiation protein 2

NM_152400 chromosome 4 open reading frame 32

NM_020179 hypothetical LOC728675; chromosome 11 open reading frame 75

NM_018906 protocadherin alpha 3

NM_012329 monocyte to macrophage differentiation-associated

NM_018910 protocadherin alpha 7

NM_173683 XK, Kell blood group complex subunit-related family, member 6

NM_138391 transmembrane protein 183A; transmembrane protein 183B

**Healthy gd20 Endometrium > Healthy gd20 Trophoblast**

**Gene Functional Classification Result**

**Gene Group 1 Enrichment Score: 6.5330010122435045**

**REFSEQ_MRNA Gene Name**

NM_005595 nuclear factor I/A

NM_172238 transcription factor AP-2 delta (activating enhancer binding protein 2 delta)

NM_005524 hairy and enhancer of split 1, (Drosophila)

NM_015265 SATB homeobox 2

NM_021813 BTB and CNC homology 1, basic leucine zipper transcription factor 2

NM_002202 ISL LIM homeobox 1

NM_021961 TEA domain family member 1 (SV40 transcriptional enhancer factor)

NM_022462 hypoxia inducible factor 3, alpha subunit

NM_018951 homeobox A10

NM_152622 mesoderm induction early response 1, family member 3

NM_020856 teashirt zinc finger homeobox 3

NM_003721 regulatory factor X-associated ankyrin-containing protein

NM_024501 homeobox D1

NM_003222 transcription factor AP-2 gamma (activating enhancer binding protein 2 gamma)

NM_002148 homeobox D10

NM_003244 TGFB-induced factor homeobox 1

NM_005596 nuclear factor I/B

NM_147192 diencephalon/mesencephalon homeobox 1

NM_153607 chromosome 5 open reading frame 41

NM_021145 cyclin D binding myb-like transcription factor 1

NM_021728 orthodenticle homeobox 2

NM_178010, NM_006940 SRY (sex determining region Y)-box 5

NM_004575 POU class 4 homeobox 2

NM_153620 homeobox A1

NM_053002 mediator complex subunit 12-like

NM_006874 E74-like factor 2 (ets domain transcription factor)

NM_022728 neurogenic differentiation 6

NM_006465 AT rich interactive domain 3B (BRIGHT-like)

NM_032440 ligand dependent nuclear receptor corepressor

NM_021809 TGFB-induced factor homeobox 2

NM_001186 BTB and CNC homology 1, basic leucine zipper transcription factor 1

NM_018416 forkhead box J2

NM_030751 zinc finger E-box binding homeobox 1

NM_006164 nuclear factor (erythroid-derived 2)-like 2

NM_006195 pre-B-cell leukemia homeobox 3

NM_030661 homeobox A3

NM_007162 transcription factor EB

NM_003112 Sp4 transcription factor

NM_002382 MYC associated factor X

**Gene Group 2 Enrichment Score: 6.133276011755014**

**REFSEQ_MRNA Gene Name**

NM_172238 transcription factor AP-2 delta (activating enhancer binding protein 2 delta)

NM_006951 TAF5 RNA polymerase II, TATA box binding protein (TBP)-associated factor, 100kDa

NM_003074 SWI/SNF related, matrix associated, actin dependent regulator of chromatin, subfamily c, member 1

NM_004229 mediator complex subunit 14

NM_015265 SATB homeobox 2

NM_001621 aryl hydrocarbon receptor

NM_021961 TEA domain family member 1 (SV40 transcriptional enhancer factor)

NM_004379 cAMP responsive element binding protein 1

NM_014034 ASF1 anti-silencing function 1 homolog A (S. cerevisiae)

NM_032329 inhibitor of growth family, member 5

NM_015138 Rtf1, Paf1/RNA polymerase II complex component, homolog (S. cerevisiae)

NM_002148 homeobox D10

NM_001001890 runt-related transcription factor 1

NM_004821 heart and neural crest derivatives expressed 1

NM_005596 nuclear factor I/B

NM_147192 diencephalon/mesencephalon homeobox 1

NM_006791 mortality factor 4; mortality factor 4 like 1

NM_003822 nuclear receptor subfamily 5, group A, member 2

NM_005316 general transcription factor IIH, polypeptide 1, 62kDa

NM_001949 E2F transcription factor 3

NM_057175 NMDA receptor regulated 1

NM_005225 E2F transcription factor 1

NM_015995 Kruppel-like factor 13

NM_012081 elongation factor, RNA polymerase II, 2

NM_022659 early B-cell factor 2

NM_178010, NM_006940 SRY (sex determining region Y)-box 5

NM_014071 nuclear receptor coactivator 6

NM_207291 upstream transcription factor 2, c-fos interacting

NM_053002 mediator complex subunit 12-like

NM_006874 E74-like factor 2 (ets domain transcription factor)

NM_001186 BTB and CNC homology 1, basic leucine zipper transcription factor 1

NM_018416 forkhead box J2

NM_030751 zinc finger E-box binding homeobox 1

NM_006164 nuclear factor (erythroid-derived 2)-like 2

NM_000192 T-box 5

NM_007162 transcription factor EB

NM_003112 Sp4 transcription factor

NM_002382 MYC associated factor X

NM_006106 Yes-associated protein 1, 65kDa

**Gene Group 3 Enrichment Score: 5.461126960428628**

**REFSEQ_MRNA Gene Name**

NM_000393 collagen, type V, alpha 2

NM_001844 collagen, type II, alpha 1

NM_000094 collagen, type VII, alpha 1

NM_001854 collagen, type XI, alpha 1

NM_004370 collagen, type XII, alpha 1

NM_152890 collagen, type XXIV, alpha 1

NM_001858 collagen, type XIX, alpha 1

NM_015719 collagen, type V, alpha 3

NM_000091 collagen, type IV, alpha 3 (Goodpasture antigen)

NM_001855 collagen, type XV, alpha 1

NM_000088 collagen, type I, alpha 1

NM_000089 collagen, type I, alpha 2

NM_004369 collagen, type VI, alpha 3

NM_001845 collagen, type IV, alpha 1

NM_000092 collagen, type IV, alpha 4

NM_001846 collagen, type IV, alpha 2

NM_001851 collagen, type IX, alpha 1

NM_000090 collagen, type III, alpha 1

**Gene Group 4 Enrichment Score: 4.154413036435907**

**REFSEQ_MRNA Gene Name**

NM_016090 RNA binding motif protein 7

NM_018211 ribonucleoprotein, PTB-binding 2

NM_007007 cleavage and polyadenylation specific factor 6, 68kDa

NM_005777 RNA binding motif protein 6

NM_030627 cytoplasmic polyadenylation element binding protein 4

NM_006547 insulin-like growth factor 2 mRNA binding protein 3

NM_004432 ELAV (embryonic lethal, abnormal vision, Drosophila)-like 2 (Hu antigen B)

NM_182485 cytoplasmic polyadenylation element binding protein 2

**Gene Group 5 Enrichment Score: 4.117395368438662**

**REFSEQ_MRNA Gene Name**

NM_020382 SET domain containing (lysine methyltransferase) 8

NM_005862 stromal antigen 1

NM_015032 PDS5, regulator of cohesion maintenance, homolog B (S. cerevisiae)

NM_152510 HORMA domain containing 2

NM_201567, NM_001789 cell division cycle 25 homolog A (S. pombe)

NM_024808 chromosome 13 open reading frame 34

NM_004060 cyclin G1

NM_003483 high mobility group AT-hook 2

**Gene Group 6 Enrichment Score: 4.0544493029823725**

**REFSEQ_MRNA Gene Name**

NM_144653 NACC family member 2, BEN and BTB (POZ) domain containing

NM_172238 transcription factor AP-2 delta (activating enhancer binding protein 2 delta)

NM_002167 inhibitor of DNA binding 3, dominant negative helix-loop-helix protein

NM_018433 lysine (K)-specific demethylase 3A

NM_006624 zinc finger, MYND domain containing 11

NM_002971 SATB homeobox 1

NM_015534 zinc finger, ZZ-type containing 3

NM_015265 SATB homeobox 2

NM_012234 RING1 and YY1 binding protein

NM_015339 activity-dependent neuroprotector homeobox

NM_004349 runt-related transcription factor 1; translocated to, 1 (cyclin D-related)

NM_021961 TEA domain family member 1 (SV40 transcriptional enhancer factor)

NM_003575 zinc finger protein 282

NM_017544 NFKB repressing factor

NM_003111 Sp3 transcription factor

NM_012421 rearranged L-myc fusion

NM_003150 signal transducer and activator of transcription 3 (acute-phase response factor)

NM_005234 nuclear receptor subfamily 2, group F, member 6

NM_022898 B-cell CLL/lymphoma 11B (zinc finger protein)

NM_015094 hypermethylated in cancer 2

NM_004973 jumonji, AT rich interactive domain 2

NM_020228 PR domain containing 10

NM_020856 teashirt zinc finger homeobox 3

NM_181353 inhibitor of DNA binding 1, dominant negative helix-loop-helix protein

NM_006352 zinc finger protein 238

NM_004926 zinc finger protein 36, C3H type-like 1

NM_030625 tet oncogene 1

NM_032329 inhibitor of growth family, member 5

NM_003244 TGFB-induced factor homeobox 1

NM_004821 heart and neural crest derivatives expressed 1

NM_005180 BMI1 polycomb ring finger oncogene

NM_005596 nuclear factor I/B

NM_013450 bromodomain adjacent to zinc finger domain, 2B

NM_147192 diencephalon/mesencephalon homeobox 1

NM_014491 forkhead box P2

NM_018133 male-specific lethal 2 homolog (Drosophila)

NM_003822 nuclear receptor subfamily 5, group A, member 2

NM_182907 PR domain containing 1, with ZNF domain

NM_005225 E2F transcription factor 1

NM_002655 pleiomorphic adenoma gene 1

NM_003036 v-ski sarcoma viral oncogene homolog (avian)

NM_015995 Kruppel-like factor 13

NM_022659 early B-cell factor 2

NM_015481 zinc finger protein 385A

NM_006874 E74-like factor 2 (ets domain transcription factor)

NM_002166 inhibitor of DNA binding 2, dominant negative helix-loop-helix protein

NM_032440 ligand dependent nuclear receptor corepressor

NM_001186 BTB and CNC homology 1, basic leucine zipper transcription factor 1

NM_022781 ring finger protein 38

NM_003597 Kruppel-like factor 11

NM_017742 zinc finger, CCHC domain containing 2

NM_152493 zinc finger protein 362

NM_003453 zinc finger, MYM-type 2

NM_030751 zinc finger E-box binding homeobox 1

NM_022552 DNA (cytosine-5-)-methyltransferase 3 alpha

NM_022893 B-cell CLL/lymphoma 11A (zinc finger protein)

NM_003412 Zic family member 1 (odd-paired homolog, Drosophila)

NM_007162 transcription factor EB

NM_004992 methyl CpG binding protein 2 (Rett syndrome)

NM_003112 Sp4 transcription factor

NM_138473 Sp1 transcription factor

NM_004241 jumonji domain containing 1C

NM_152478 zinc finger protein 583

**Gene Group 7 Enrichment Score: 3.1584852919952087**

**REFSEQ_MRNA Gene Name**

NM_152835 PDLIM1 interacting kinase 1 like

NM_003390 WEE1 homolog (S. pombe)

NM_032430 BR serine/threonine kinase 1

NM_030952 NUAK family, SNF1-like kinase, 2

NM_007170 testis-specific kinase 2

NM_018571 STE20-related kinase adaptor beta

NM_003010 mitogen-activated protein kinase kinase 4

NM_022894 poly(A) polymerase gamma

NM_021643 tribbles homolog 2 (Drosophila)

NM_198465 Nik related kinase

NM_005627 serum/glucocorticoid regulated kinase 1

NM_004734 doublecortin-like kinase 1

NM_014397 NIMA (never in mitosis gene a)-related kinase 6

NM_012290 tousled-like kinase 1

NM_002314 LIM domain kinase 1

NM_032017 serine/threonine kinase 40

NM_003618 mitogen-activated protein kinase kinase kinase kinase 3

NM_002595 PCTAIRE protein kinase 2

NM_005734 homeodomain interacting protein kinase 3

NM_001222 calcium/calmodulin-dependent protein kinase II gamma

NM_001259 cyclin-dependent kinase 6

NM_145259 activin A receptor, type IC

NM_017719 SNF related kinase

NM_020778 alpha-kinase 3

NM_175854 PAN3 poly(A) specific ribonuclease subunit homolog (S. cerevisiae)

NM_170709 serum/glucocorticoid regulated kinase family, member 3

NM_001895 casein kinase 2, alpha 1 polypeptide pseudogene; casein kinase 2, alpha 1 polypeptide

NM_006852 tousled-like kinase 2

NM_032435 mixed lineage kinase 4

NM_014572 LATS, large tumor suppressor, homolog 2 (Drosophila)

NM_020921 ninein (GSK3B interacting protein)

**Gene Group 8 Enrichment Score: 2.818550765117314**

**REFSEQ_MRNA Gene Name**

NM_004612 transforming growth factor, beta receptor 1

NM_001616 activin A receptor, type IIA

NM_001204 bone morphogenetic protein receptor, type II (serine/threonine kinase)

NM_145259 activin A receptor, type IC

NM_007170 testis-specific kinase 2

NM_003242 transforming growth factor, beta receptor II (70/80kDa)

**Gene Group 9 Enrichment Score: 2.81062035660957**

**REFSEQ_MRNA Gene Name**

NM_014397 NIMA (never in mitosis gene a)-related kinase 6

NM_004856 kinesin family member 23

NM_015032 PDS5, regulator of cohesion maintenance, homolog B (S. cerevisiae)

NM_006306 structural maintenance of chromosomes 1A

NM_194261 ubiquitin-conjugating enzyme E2I (UBC9 homolog, yeast)

**Gene Group 10 Enrichment Score: 2.798762142456355**

**REFSEQ_MRNA Gene Name**

NM_006924 splicing factor, arginine/serine-rich 1

NM_018211 ribonucleoprotein, PTB-binding 2

NM_007007 cleavage and polyadenylation specific factor 6, 68kDa

NM_054016 FUS interacting protein (serine/arginine-rich) 1; similar to FUS interacting protein (serine-arginine rich) 1

NM_005105 RNA binding motif protein 8A

NM_018061 PRP38 pre-mRNA processing factor 38 (yeast) domain containing B

NM_016312 WW domain binding protein 11

NM_145893 ataxin 2-binding protein 1

**Gene Group 11 Enrichment Score: 2.7168935694010674**

**REFSEQ_MRNA Gene Name**

NM_020382 SET domain containing (lysine methyltransferase) 8

NM_003173 suppressor of variegation 3-9 homolog 1 (Drosophila)

NM_017635 suppressor of variegation 4-20 homolog 1 (Drosophila)

NM_175709 chromobox homolog 7

NM_001991 enhancer of zeste homolog 1 (Drosophila)

NM_032701 suppressor of variegation 4-20 homolog 2 (Drosophila)

**Gene Group 12 Enrichment Score: 2.622419031581591**

**REFSEQ_MRNA Gene Name**

NM_002833 protein tyrosine phosphatase, non-receptor type 9

NM_030640 dual specificity phosphatase 16

NM_033389 slingshot homolog 2 (Drosophila)

NM_014369 protein tyrosine phosphatase, non-receptor type 18 (brain-derived)

NM_003463 protein tyrosine phosphatase type IVA, member 1

**Gene Group 13 Enrichment Score: 2.424035814179844**

**REFSEQ_MRNA Gene Name**

NM_016271 ring finger protein 138

NM_017742 zinc finger, CCHC domain containing 2

NM_005180 BMI1 polycomb ring finger oncogene

NM_152493 zinc finger protein 362

NM_030576 LIM domain containing 2

NM_017610 ring finger protein 111

NM_152896 ubiquitin-like with PHD and ring finger domains 2

NM_015457 zinc finger, DHHC-type containing 5

NM_001039111 tripartite motif-containing 71

NM_033089 zinc finger, CCHC domain containing 3

NM_172070 ubiquitin protein ligase E3 component n-recognin 3 (putative)

NM_001002909 G patch domain containing 8

NM_032588 tripartite motif-containing 63

NM_015534 zinc finger, ZZ-type containing 3

NM_015435 ring finger protein 19A

NM_015346 zinc finger, FYVE domain containing 26

NM_022781 ring finger protein 38

NM_152787 mitogen-activated protein kinase kinase kinase 7 interacting protein 3

NM_018133 male-specific lethal 2 homolog (Drosophila)

**Gene Group 14 Enrichment Score: 2.3965811050537877**

**REFSEQ_MRNA Gene Name**

NM_003794 sorting nexin 4

NM_030918 sorting nexin family member 27

NM_022133 sorting nexin 16

NM_014035 sorting nexin 24

**Gene Group 15 Enrichment Score: 2.331567314645012**

**REFSEQ_MRNA Gene Name**

NM_014494 trinucleotide repeat containing 6A

NM_006546 insulin-like growth factor 2 mRNA binding protein 1

NM_017629 eukaryotic translation initiation factor 2C, 4

NM_006547 insulin-like growth factor 2 mRNA binding protein 3

**Gene Group 16 Enrichment Score: 1.792367648903954**

**REFSEQ_MRNA Gene Name**

NM_003939 beta-transducin repeat containing

NM_019063 echinoderm microtubule associated protein like 4

NM_017641 kinesin family member 21A

NM_153252 bromodomain and WD repeat domain containing 3

NM_015891 cell division cycle 40 homolog (S. cerevisiae)

NM_004411 dynein, cytoplasmic 1, intermediate chain 1

NM_015626 WD repeat and SOCS box-containing 1

NM_017749 autophagy/beclin-1 regulator 1

NM_018315 F-box and WD repeat domain containing 7

**Gene Group 17 Enrichment Score: 1.5856794854754213**

**REFSEQ_MRNA Gene Name**

NM_001358 DEAH (Asp-Glu-Ala-His) box polypeptide 15

NM_020159 SWI/SNF-related, matrix-associated actin-dependent regulator of chromatin, subfamily a, containing DEAD/H box 1

NM_198963 DEAH (Asp-Glu-Ala-Asp/His) box polypeptide 57

NM_018332 DEAD (Asp-Glu-Ala-As) box polypeptide 19A

**Gene Group 18 Enrichment Score: 1.5020253483131072**

**REFSEQ_MRNA Gene Name**

NM_004359 cell division cycle 34 homolog (S. cerevisiae)

NM_017582 ubiquitin-conjugating enzyme E2Q family member 1

NM_003338 ubiquitin-conjugating enzyme E2D 1 (UBC4/5 homolog, yeast)

NM_003348 ubiquitin-conjugating enzyme E2N (UBC13 homolog, yeast)

**Gene Group 19 Enrichment Score: 1.4798390994153934**

**REFSEQ_MRNA Gene Name**

NM_021183 RAP2C, member of RAS oncogene family

NM_016131 RAB10, member RAS oncogene family

NM_012250 related RAS viral (r-ras) oncogene homolog 2; similar to related RAS viral (r-ras) oncogene homolog 2

NM_016544 DnaJ (Hsp40) homolog, subfamily C, member 27

NM_002524 neuroblastoma RAS viral (v-ras) oncogene homolog

NM_021033 RAP2A, member of RAS oncogene family

**Gene Group 20 Enrichment Score: 1.4352562793647052**

**REFSEQ_MRNA Gene Name**

NM_004856 kinesin family member 23

NM_017641 kinesin family member 21A

NM_020921 ninein (GSK3B interacting protein)

NM_015074 kinesin family member 1B

**Gene Group 21 Enrichment Score: 1.3947570596936267**

**REFSEQ_MRNA Gene Name**

NM_153810 chromosome 10 open reading frame 46

NM_013396 ubiquitin specific peptidase 25

NM_080867 suppressor of cytokine signaling 4

NM_004656 BRCA1 associated protein-1 (ubiquitin carboxy-terminal hydrolase)

NM_006313 ubiquitin specific peptidase 15

NM_014871 PAN2 poly(A) specific ribonuclease subunit homolog (S. cerevisiae)

NM_022832 ubiquitin specific peptidase 46

NM_001001664 speckle-type POZ protein-like

NM_015626 WD repeat and SOCS box-containing 1

NM_032557 ubiquitin specific peptidase 38

NM_080862 splA/ryanodine receptor domain and SOCS box containing 4

NM_004232 suppressor of cytokine signaling 6

**Gene Group 22 Enrichment Score: 1.3060732585456851**

**REFSEQ_MRNA Gene Name**

NM_033542 SYS1 Golgi-localized integral membrane protein homolog (S. cerevisiae)

NM_016072 golgi transport 1 homolog B (S. cerevisiae)

NM_001001433 syntaxin 16

NM_177424 syntaxin 12

**Gene Group 23 Enrichment Score: 1.283496946768907**

**REFSEQ_MRNA Gene Name**

NM_002033 fucosyltransferase 4 (alpha (1,3) fucosyltransferase, myeloid-specific)

NM_004616 tetraspanin 8

NM_152996 ST6 (alpha-N-acetyl-neuraminyl-2,3-beta-galactosyl-1,3)-N-acetylgalactosaminide alpha-2,6-sialyltransferase 3

NM_014918 chondroitin sulfate synthase 1

NM_020474 UDP-N-acetyl-alpha-D-galactosamine:polypeptide N-acetylgalactosaminyltransferase 13 (GalNAc-T13); UDP-N-acetyl-alpha-D-galactosamine:polypeptide N-acetylgalactosaminyltransferase 1 (GalNAc-T1)

NM_016548 golgi membrane protein 1

NM_006876 UDP-GlcNAc:betaGal beta-1,3-N-acetylglucosaminyltransferase 1; UDP-GlcNAc:betaGal beta-1,3-N-acetylglucosaminyltransferase 2

NM_017423 UDP-N-acetyl-alpha-D-galactosamine:polypeptide N-acetylgalactosaminyltransferase 7 (GalNAc-T7)

NM_003779 UDP-Gal:betaGlcNAc beta 1,4- galactosyltransferase, polypeptide 3

NM_003782 UDP-Gal:betaGlcNAc beta 1,3-galactosyltransferase, polypeptide 4

NM_016591 glucosaminyl (N-acetyl) transferase 4, core 2 (beta-1,6-N-acetylglucosaminyltransferase)

NM_003783 UDP-Gal:betaGlcNAc beta 1,3-galactosyltransferase, polypeptide 2

NM_015554 glucuronic acid epimerase

NM_005329 hyaluronan synthase 3

NM_020438 dolichyl pyrophosphate phosphatase 1

NM_032859 abhydrolase domain containing 13

NM_005907 mannosidase, alpha, class 1A, member 1

**Gene Group 24 Enrichment Score: 0.8367098618789921**

**REFSEQ_MRNA Gene Name**

NM_014283 chromosome 1 open reading frame 9

NM_053039 UDP glucuronosyltransferase 2 family, polypeptide B28

NM_001077 UDP glucuronosyltransferase 2 family, polypeptide B17

NM_001076 UDP glucuronosyltransferase 2 family, polypeptide B15

**Gene Group 25 Enrichment Score: 0.7905598390052044**

**REFSEQ_MRNA Gene Name**

NM_182920 ADAM metallopeptidase with thrombospondin type 1 motif, 9

NM_007038 ADAM metallopeptidase with thrombospondin type 1 motif, 5

NM_006988 ADAM metallopeptidase with thrombospondin type 1 motif, 1

NM_002581 PAPPA antisense RNA (non-protein coding); pregnancy-associated plasma protein A, pappalysin 1

**Gene Group 26 Enrichment Score: 0.5063879485702223**

**REFSEQ_MRNA Gene Name**

NM_002250 potassium intermediate/small conductance calcium-activated channel, subfamily N, member 4

NM_002998 syndecan 2

NM_031418 anoctamin 3

NM_005302 G protein-coupled receptor 37 (endothelin receptor type B-like)

NM_139177 solute carrier family 39 (metal ion transporter), member 11

NM_001046 solute carrier family 12 (sodium/potassium/chloride transporters), member 2

NM_003759 solute carrier family 4, sodium bicarbonate cotransporter, member 4

NM_000891 potassium inwardly-rectifying channel, subfamily J, member 2

NM_018375 solute carrier family 39 (zinc transporter), member 9

NM_018976 solute carrier family 38, member 2

NM_016548 golgi membrane protein 1

NM_022058 solute carrier family 4, sodium bicarbonate transporter, member 10

NM_002246 potassium channel, subfamily K, member 3

NM_025250 tweety homolog 3 (Drosophila)

NM_018992 potassium channel tetramerisation domain containing 5

NM_007231 solute carrier family 6 (amino acid transporter), member 14

NM_005415 solute carrier family 20 (phosphate transporter), member 1

NM_152527 solute carrier family 16, member 14 (monocarboxylic acid transporter 14)

NM_078483 solute carrier family 36 (proton/amino acid symporter), member 1

NM_005329 hyaluronan synthase 3

NM_018593 solute carrier family 16, member 10 (aromatic amino acid transporter)

**Gene Group 27 Enrichment Score: 0.46948488417659984**

**REFSEQ_MRNA Gene Name**

NM_213609 family with sequence similarity 19 (chemokine (C-C motif)-like), member A1

NM_153689 chromosome 2 open reading frame 69

NM_152745 neurexophilin 1

NM_014888 family with sequence similarity 3, member C

NM_178565 R-spondin 2 homolog (Xenopus laevis)

NM_014421 dickkopf homolog 2 (Xenopus laevis)

**Gene Group 28 Enrichment Score: 0.4359789568113238**

**REFSEQ_MRNA Gene Name**

NM_014813 leucine-rich repeats and immunoglobulin-like domains 2

NM_018908 protocadherin alpha 5

NM_006016 CD164 molecule, sialomucin

NM_018907, NM_018900 protocadherin alpha 1; protocadherin alpha 4

NM_002998 syndecan 2

NM_018930 protocadherin beta 10; protocadherin beta 9

NM_052910 SLIT and NTRK-like family, member 1

NM_000870 5-hydroxytryptamine (serotonin) receptor 4

NM_006754 synaptophysin-like 1

NM_020403 protocadherin 9

NM_004801 neurexin 1

NM_024081 proline rich Gla (G-carboxyglutamic acid) 4 (transmembrane)

NM_012302 latrophilin 2

NM_005561 lysosomal-associated membrane protein 1

NM_006378 sema domain, immunoglobulin domain (Ig), transmembrane domain (TM) and short cytoplasmic domain, (semaphorin) 4D

NM_138973 beta-site APP-cleaving enzyme 1

NM_018899, NM_018898, NM_018901, NM_018904 protocadherin alpha 13; protocadherin alpha 10; protocadherin alpha subfamily C, 1; protocadherin alpha subfamily C, 2

NM_002207 integrin, alpha 9

NM_022143 leucine rich repeat containing 4

NM_152527 solute carrier family 16, member 14 (monocarboxylic acid transporter 14)

NM_014373 G protein-coupled receptor 160

NM_017680 asporin

NM_006464 trans-golgi network protein 2

NM_153619 sema domain, transmembrane domain (TM), and cytoplasmic domain, (semaphorin) 6D

NM_000958 prostaglandin E receptor 4 (subtype EP4)

NM_001795 cadherin 5, type 2 (vascular endothelium)

NM_005302 G protein-coupled receptor 37 (endothelin receptor type B-like)

NM_018903 protocadherin alpha 12

NM_002644 polymeric immunoglobulin receptor

NM_018976 solute carrier family 38, member 2

NM_018905 protocadherin alpha 2

NM_018490 leucine-rich repeat-containing G protein-coupled receptor 4

NM_016548 golgi membrane protein 1

NM_031418 anoctamin 3

NM_012129 claudin 12

NM_005927 microfibrillar-associated protein 3

NM_003759 solute carrier family 4, sodium bicarbonate cotransporter, member 4

NM_005329 hyaluronan synthase 3

NM_018909, NM_018911 protocadherin alpha 8; protocadherin alpha 6

NM_025250 tweety homolog 3 (Drosophila)

NM_018906 protocadherin alpha 3

NM_012329 monocyte to macrophage differentiation-associated

NM_018910 protocadherin alpha 7

NM_153377 leucine-rich repeats and immunoglobulin-like domains 3

NM_022484 transmembrane protein 168

NM_013281 fibronectin leucine rich transmembrane protein 3

**Gene Group 29 Enrichment Score: 0.1755408045198475**

**REFSEQ_MRNA Gene Name**

NM_018126 transmembrane protein 33

NM_138391 transmembrane protein 183A; transmembrane protein 183B

NM_153365 transmembrane anterior posterior transformation 1

NM_014373 G protein-coupled receptor 160

NM_152400 chromosome 4 open reading frame 32

NM_152996 ST6 (alpha-N-acetyl-neuraminyl-2,3-beta-galactosyl-1,3)-N-acetylgalactosaminide alpha-2,6-sialyltransferase 3

NM_006698 bladder cancer associated protein

NM_032859 abhydrolase domain containing 13

NM_013390 transmembrane protein 2

NM_031434 transmembrane and ubiquitin-like domain containing 1

NM_022484 transmembrane protein 168

NM_014056 similar to HIG1 domain family, member 1A; HIG1 hypoxia inducible domain family, member 1A; HIG1 hypoxia inducible domain family, member 1D

NM_032947 MSTP150

NM_138340 abhydrolase domain containing 3

NM_138390 transmembrane protein 169

NM_016548 golgi membrane protein 1

NM_178454 DNA-damage regulated autophagy modulator 2

NM_014283 chromosome 1 open reading frame 9

NM_017938 family with sequence similarity 70, member A

NM_182511 cerebellin 2 precursor

NM_017911 family with sequence similarity 118, member A

NM_015008 transmembrane and coiled-coil domain family 1

NM_016591 glucosaminyl (N-acetyl) transferase 4, core 2 (beta-1,6-N-acetylglucosaminyltransferase)

NM_004616 tetraspanin 8

NM_003782 UDP-Gal:betaGlcNAc beta 1,3-galactosyltransferase, polypeptide 4

NM_007001 solute carrier family 35, member D2

NM_152261 chromosome 12 open reading frame 23

NM_018710 transmembrane protein 55A

NM_153226 transmembrane protein 20

NM_152527 solute carrier family 16, member 14 (monocarboxylic acid transporter 14)

NM_005329 hyaluronan synthase 3

NM_005927 microfibrillar-associated protein 3

NM_018593 solute carrier family 16, member 10 (aromatic amino acid transporter)

NM_173683 XK, Kell blood group complex subunit-related family, member 6

**Healthy gd20 Trophoblast > Healthy gd20 Endometrium**

**Gene Functional Classification Result**

**Gene Group 1 Enrichment Score: 5.582631706718539**

**REFSEQ_MRNA Gene Name**

NM_005955 metal-regulatory transcription factor 1

NM_003074 SWI/SNF related, matrix associated, actin dependent regulator of chromatin, subfamily c, member 1

NM_199072 MyoD family inhibitor domain containing

NM_012082 zinc finger protein, multitype 2

NM_012234 RING1 and YY1 binding protein

NM_004459 bromodomain PHD finger transcription factor

NM_021961 TEA domain family member 1 (SV40 transcriptional enhancer factor)

NM_015035 zinc fingers and homeoboxes 3

NM_005604 POU class 3 homeobox 2

NM_001452 forkhead box F2

NM_001438 estrogen-related receptor gamma

NM_003107 SRY (sex determining region Y)-box 4

NM_001001928 peroxisome proliferator-activated receptor alpha

NM_004235 Kruppel-like factor 4 (gut)

NM_006352 zinc finger protein 238

NM_013354 CCR4-NOT transcription complex, subunit 7

NM_006599 nuclear factor of activated T-cells 5, tonicity-responsive

NM_005935 AF4/FMR2 family, member 1

NM_014682 suppression of tumorigenicity 18 (breast carcinoma) (zinc finger protein)

NM_002938 ring finger protein 4; hypothetical LOC644006

NM_001754 runt-related transcription factor 1

NM_004898 clock homolog (mouse)

NM_005316 general transcription factor IIH, polypeptide 1, 62kDa

NM_057175 NMDA receptor regulated 1

NM_015995 Kruppel-like factor 13

NM_012340 nuclear factor of activated T-cells, cytoplasmic, calcineurin-dependent 2

NM_002518 neuronal PAS domain protein 2

NM_000248 microphthalmia-associated transcription factor

NM_053002 mediator complex subunit 12-like

NM_014945 actin binding LIM protein family, member 3

NM_024426 Wilms tumor 1

NM_002500 neurogenic differentiation 1

NM_003070 SWI/SNF related, matrix associated, actin dependent regulator of chromatin, subfamily a, member 2

NM_017519 AT rich interactive domain 1B (SWI1-like)

NM_005461 v-maf musculoaponeurotic fibrosarcoma oncogene homolog B (avian)

NM_145905 hypothetical LOC100130009; high mobility group AT-hook 1

NM_004571 PBX/knotted 1 homeobox 1

**Gene Group 2 Enrichment Score: 5.483205955997709**

**REFSEQ_MRNA Gene Name**

NM_020310 MAX binding protein

NM_020307 cyclin L1

NM_016169 suppressor of fused homolog (Drosophila)

NM_032458 PHD finger protein 6

NM_006951 TAF5 RNA polymerase II, TATA box binding protein (TBP)-associated factor, 100kDa

NM_030762 basic helix-loop-helix family, member e41

NM_006813 proline-rich nuclear receptor coactivator 1

NM_021961 TEA domain family member 1 (SV40 transcriptional enhancer factor)

NM_024680 E2F transcription factor 8

NM_001452 forkhead box F2

NM_020432 putative homeodomain transcription factor 2

NM_203394 E2F transcription factor 7

NM_018951 homeobox A10

NM_020856 teashirt zinc finger homeobox 3

NM_023929 zinc finger and BTB domain containing 10

NM_182574 MEF2 activating motif and SAP domain containing transcriptional regulator

NM_013354 CCR4-NOT transcription complex, subunit 7

NM_003222 transcription factor AP-2 gamma (activating enhancer binding protein 2 gamma)

NM_015138 Rtf1, Paf1/RNA polymerase II complex component, homolog (S. cerevisiae)

NM_005599 nescient helix loop helix 2

NM_005924 mesenchyme homeobox 2

NM_014552 grainyhead-like 1 (Drosophila)

NM_003216 thyrotrophic embryonic factor

NM_005316 general transcription factor IIH, polypeptide 1, 62kDa

NM_153607 chromosome 5 open reading frame 41

NM_022658 homeobox C8

NM_153450 mediator complex subunit 19

NM_002687 pinin, desmosome associated protein

NM_021145 cyclin D binding myb-like transcription factor 1

NM_007375 TAR DNA binding protein

NM_012081 elongation factor, RNA polymerase II, 2

NM_021728 orthodenticle homeobox 2

NM_005230 ELK3, ETS-domain protein (SRF accessory protein 2)

NM_053002 mediator complex subunit 12-like

NM_003185 TAF4 RNA polymerase II, TATA box binding protein (TBP)-associated factor, 135kDa

NM_032440 ligand dependent nuclear receptor corepressor

NM_021809 TGFB-induced factor homeobox 2

NM_012257 HMG-box transcription factor 1

NM_001241 cyclin T2

NM_017519 AT rich interactive domain 1B (SWI1-like)

NM_001310 cAMP responsive element binding protein-like 2

NM_017778 Wolf-Hirschhorn syndrome candidate 1-like 1

NM_006195 pre-B-cell leukemia homeobox 3

NM_030661 homeobox A3

NM_003077 SWI/SNF related, matrix associated, actin dependent regulator of chromatin, subfamily d, member 2

NM_018200 high-mobility group 20A

NM_005461 v-maf musculoaponeurotic fibrosarcoma oncogene homolog B (avian)

NM_001991 enhancer of zeste homolog 1 (Drosophila)

**Gene Group 3 Enrichment Score: 5.142784650823119**

**REFSEQ_MRNA Gene Name**

NM_007259 vacuolar protein sorting 45 homolog (S. cerevisiae)

NM_030918 sorting nexin family member 27

NM_022459 exportin 4

NM_014426 sorting nexin 5

NM_022133 sorting nexin 16

NM_014904 RAB11 family interacting protein 2 (class I)

NM_152415 vacuolar protein sorting 37 homolog A (S. cerevisiae)

NM_014748 sorting nexin 17

**Gene Group 4 Enrichment Score: 5.02069576261253**

**REFSEQ_MRNA Gene Name**

NM_014372 ring finger protein 11

NM_178450 membrane-associated ring finger (C3HC4) 3

NM_032458 PHD finger protein 6

NM_004719 splicing factor, arginine/serine-rich 2, interacting protein

NM_016271 ring finger protein 138

NM_018263 additional sex combs like 2 (Drosophila)

NM_012234 RING1 and YY1 binding protein

NM_006048 ubiquitination factor E4B (UFD2 homolog, yeast)

NM_015153 PHD finger protein 3

NM_153812 PHD finger protein 13

NM_172070 ubiquitin protein ligase E3 component n-recognin 3 (putative)

NM_144726 ring finger protein 145

NM_025133 F-box protein 11

NM_020771 HECT domain and ankyrin repeat containing, E3 ubiquitin protein ligase 1

NM_015017 ubiquitin specific peptidase 33

NM_147128 zinc and ring finger 2

NM_020856 teashirt zinc finger homeobox 3

NM_015176 F-box protein 28

NM_023929 zinc finger and BTB domain containing 10

NM_152787 mitogen-activated protein kinase kinase kinase 7 interacting protein 3

NM_012124 cysteine and histidine-rich domain (CHORD)-containing 1; cysteine and histidine-rich domain (CHORD)-containing 1 pseudogene

NM_033414 zinc finger protein 622

NM_182757 ring finger protein 144B

NM_152553 ring finger protein 217

NM_004293 guanine deaminase

NM_017610 ring finger protein 111

NM_017582 ubiquitin-conjugating enzyme E2Q family member 1

NM_005180 BMI1 polycomb ring finger oncogene

NM_019083 coiled-coil domain containing 76

NM_144778 muscleblind-like 2 (Drosophila)

NM_003339 ubiquitin-conjugating enzyme E2D 2 (UBC4/5 homolog, yeast)

NM_022353 O-sialoglycoprotein endopeptidase-like 1

NM_015057 MYC binding protein 2

NM_006458 tripartite motif-containing 3

NM_006526 zinc finger protein 217

NM_021620 PR domain containing 13

NM_001002909 G patch domain containing 8

NM_007013 WW domain containing E3 ubiquitin protein ligase 1

NM_021943 zinc finger, AN1-type domain 3

NM_033089 zinc finger, CCHC domain containing 3

NM_016436 PHD finger protein 20

NM_001005415 membrane-associated ring finger (C3HC4) 2

NM_022781 ring finger protein 38

NM_015271 tripartite motif-containing 2

NM_017912 hect domain and RLD 6

NM_003453 zinc finger, MYM-type 2

NM_152493 zinc finger protein 362

NM_017742 zinc finger, CCHC domain containing 2

NM_152271 LON peptidase N-terminal domain and ring finger 1

NM_203301 F-box protein 33

NM_033645 F-box and WD repeat domain containing 11

NM_001001484 phosphotriesterase related

NM_006910 retinoblastoma binding protein 6

**Gene Group 5 Enrichment Score: 4.515435592664029**

**REFSEQ_MRNA Gene Name**

NM_016231 nemo-like kinase

NM_152835 PDLIM1 interacting kinase 1 like

NM_015076 cell division cycle 2-like 6 (CDK8-like)

NM_005627 serum/glucocorticoid regulated kinase 1

NM_006141 dynein, cytoplasmic 1, light intermediate chain 2

NM_003390 WEE1 homolog (S. pombe)

NM_018571 STE20-related kinase adaptor beta

NM_003010 mitogen-activated protein kinase kinase 4

NM_175854 PAN3 poly(A) specific ribonuclease subunit homolog (S. cerevisiae)

NM_005465 v-akt murine thymoma viral oncogene homolog 3 (protein kinase B, gamma)

NM_001259, NM_001145306 cyclin-dependent kinase 6

NM_020975 ret proto-oncogene

NM_017719 SNF related kinase

NM_014572 LATS, large tumor suppressor, homolog 2 (Drosophila)

NM_014683 unc-51-like kinase 2 (C. elegans)

NM_001004106 G protein-coupled receptor kinase 6

NM_001105 activin A receptor, type I

NM_015216 histidine acid phosphatase domain containing 1

NM_016308 cytidine monophosphate (UMP-CMP) kinase 1, cytosolic

NM_005813 protein kinase D3

NM_005736 ARP1 actin-related protein 1 homolog A, centractin alpha (yeast)

NM_001204 bone morphogenetic protein receptor, type II (serine/threonine kinase)

NM_004734 doublecortin-like kinase 1

NM_001616 activin A receptor, type IIA

NM_021133 ribonuclease L (2',5'-oligoisoadenylate synthetase-dependent)

NM_004755 ribosomal protein S6 kinase, 90kDa, polypeptide 5

NM_030906 serine/threonine kinase 33

NM_004443 EPH receptor B3

NM_020168 p21 protein (Cdc42/Rac)-activated kinase 6

NM_002880 v-raf-1 murine leukemia viral oncogene homolog 1

NM_012290 tousled-like kinase 1

NM_001274 CHK1 checkpoint homolog (S. pombe)

NM_020135 Werner helicase interacting protein 1

NM_139014 mitogen-activated protein kinase 14

NM_173354 salt-inducible kinase 1

NM_014278 heat shock 70kDa protein 4-like

NM_012395 PFTAIRE protein kinase 1

NM_080836 serine/threonine kinase 35

NM_002612 pyruvate dehydrogenase kinase, isozyme 4

NM_004440 EPH receptor A7

NM_017553 INO80 homolog (S. cerevisiae)

NM_002648 pim-1 oncogene

NM_006251 protein kinase, AMP-activated, alpha 1 catalytic subunit

NM_002595 PCTAIRE protein kinase 2

**Gene Group 6 Enrichment Score: 4.462739516722259**

**REFSEQ_MRNA Gene Name**

NM_032458 PHD finger protein 6

NM_020382 SET domain containing (lysine methyltransferase) 8

NM_006624 zinc finger, MYND domain containing 11

NM_014795 zinc finger E-box binding homeobox 2

NM_018263 additional sex combs like 2 (Drosophila)

NM_005955 metal-regulatory transcription factor 1

NM_012082 zinc finger protein, multitype 2

NM_012234 RING1 and YY1 binding protein

NM_015339 activity-dependent neuroprotector homeobox

NM_001379 DNA (cytosine-5-)-methyltransferase 1

NM_012406 PR domain containing 4

NM_004349 runt-related transcription factor 1; translocated to, 1 (cyclin D-related)

NM_004459 bromodomain PHD finger transcription factor

NM_021961 TEA domain family member 1 (SV40 transcriptional enhancer factor)

NM_015035 zinc fingers and homeoboxes 3

NM_199160 LIM homeobox 6

NM_024680 E2F transcription factor 8

NM_001452 forkhead box F2

NM_001438 estrogen-related receptor gamma

NM_203394 E2F transcription factor 7

NM_015094 hypermethylated in cancer 2

NM_004235 Kruppel-like factor 4 (gut)

NM_020856 teashirt zinc finger homeobox 3

NM_002968 sal-like 1 (Drosophila)

NM_023929 zinc finger and BTB domain containing 10

NM_006352 zinc finger protein 238

NM_013354 CCR4-NOT transcription complex, subunit 7

NM_033414 zinc finger protein 622

NM_014682 suppression of tumorigenicity 18 (breast carcinoma) (zinc finger protein)

NM_002938 ring finger protein 4; hypothetical LOC644006

NM_003244 TGFB-induced factor homeobox 1

NM_005180 BMI1 polycomb ring finger oncogene

NM_013450 bromodomain adjacent to zinc finger domain, 2B

NM_032497 zinc finger protein 559

NM_003216 thyrotrophic embryonic factor

NM_004535 myelin transcription factor 1

NM_006526 zinc finger protein 217

NM_021620 PR domain containing 13

NM_022658 homeobox C8

NM_175850 DNA (cytosine-5-)-methyltransferase 3 beta

NM_002655 pleiomorphic adenoma gene 1

NM_015995 Kruppel-like factor 13

NM_006734 human immunodeficiency virus type I enhancer binding protein 2

NM_005230 ELK3, ETS-domain protein (SRF accessory protein 2)

NM_016436 PHD finger protein 20

NM_013449 bromodomain adjacent to zinc finger domain, 2A

NM_032440 ligand dependent nuclear receptor corepressor

NM_024426 Wilms tumor 1

NM_022781 ring finger protein 38

NM_003453 zinc finger, MYM-type 2

NM_152493 zinc finger protein 362

NM_022893 B-cell CLL/lymphoma 11A (zinc finger protein)

NM_017778 Wolf-Hirschhorn syndrome candidate 1-like 1

NM_152735 zinc finger and BTB domain containing 9

NM_001005366 lysine (K)-specific demethylase 2B

NM_018200 high-mobility group 20A

NM_033224 purine-rich element binding protein B

NM_004992 methyl CpG binding protein 2 (Rett syndrome)

NM_001991 enhancer of zeste homolog 1 (Drosophila)

**Gene Group 7 Enrichment Score: 4.419031543758744**

**REFSEQ_MRNA Gene Name**

NM_153020 RNA binding motif protein 24

NM_014912 cytoplasmic polyadenylation element binding protein 3

NM_002897 RNA binding motif, single stranded interacting protein 1

NM_030627 cytoplasmic polyadenylation element binding protein 4

NM_182485 cytoplasmic polyadenylation element binding protein 2

**Gene Group 8 Enrichment Score: 3.777047286477188**

**REFSEQ_MRNA Gene Name**

NM_024692 CAP-GLY domain containing linker protein family, member 4

NM_020771 HECT domain and ankyrin repeat containing, E3 ubiquitin protein ligase 1

NM_015577 retinoic acid induced 14

NM_002480 protein phosphatase 1, regulatory (inhibitor) subunit 12A

**Gene Group 9 Enrichment Score: 3.618770623940577**

**REFSEQ_MRNA Gene Name**

NM_016277 RAB23, member RAS oncogene family

NM_016530 RAB8B, member RAS oncogene family

NM_016131 RAB10, member RAS oncogene family

NM_016322 RAB14, member RAS oncogene family

NM_031934 RAB34, member RAS oncogene family

NM_021183 RAP2C, member of RAS oncogene family

NM_004162 RAB5A, member RAS oncogene family

NM_014488 RAB30, member RAS oncogene family

NM_004040 ras homolog gene family, member B

NM_021252 RAB18, member RAS oncogene family

NM_014999 RAB21, member RAS oncogene family

NM_016370 RAB9B, member RAS oncogene family

NM_198686 RAB15, member RAS onocogene family

**Gene Group 10 Enrichment Score: 3.373297019722361**

**REFSEQ_MRNA Gene Name**

NM_025187 chromosome 16 open reading frame 70

NM_001002243 aftiphilin

NM_004922 SEC24 family, member C (S. cerevisiae)

NM_014203 adaptor-related protein complex 2, alpha 1 subunit

NM_004859 clathrin, heavy chain (Hc)

**Gene Group 11 Enrichment Score: 3.3189836160846107**

**REFSEQ_MRNA Gene Name**

NM_153810 chromosome 10 open reading frame 46

NM_013396 ubiquitin specific peptidase 25

NM_004505 ubiquitin specific peptidase 6 (Tre-2 oncogene)

NM_006313 ubiquitin specific peptidase 15

NM_015017 ubiquitin specific peptidase 33

NM_203301 F-box protein 33

NM_032582 similar to TBC1 domain family, member 3; ubiquitin specific peptidase 32

NM_014372 ring finger protein 11

NM_032236 ubiquitin specific peptidase 48

NM_004232 suppressor of cytokine signaling 6

NM_015176 F-box protein 28

**Gene Group 12 Enrichment Score: 3.102806141449564**

**REFSEQ_MRNA Gene Name**

NM_017641 kinesin family member 21A

NM_012223 myosin IB

NM_015074 kinesin family member 1B

NM_005736 ARP1 actin-related protein 1 homolog A, centractin alpha (yeast)

NM_004984 kinesin family member 5A

NM_004856 kinesin family member 23

NM_006141 dynein, cytoplasmic 1, light intermediate chain 2

NM_004521 kinesin family member 5B

NM_014278 heat shock 70kDa protein 4-like

NM_007054 kinesin family member 3A

**Gene Group 13 Enrichment Score: 3.0624449010296844**

**REFSEQ_MRNA Gene Name**

NM_025134 chromodomain helicase DNA binding protein 9

NM_003070 SWI/SNF related, matrix associated, actin dependent regulator of chromatin, subfamily a, member 2

NM_003972 BTAF1 RNA polymerase II, B-TFIID transcription factor-associated, 170kDa (Mot1 homolog, S. cerevisiae)

NM_000489 alpha thalassemia/mental retardation syndrome X-linked (RAD54 homolog, S. cerevisiae)

NM_017553 INO80 homolog (S. cerevisiae)

**Gene Group 14 Enrichment Score: 2.9959172879976452**

**REFSEQ_MRNA Gene Name**

NM_025180 centrosomal protein 63kDa

NM_018451 centromere protein J

NM_006997 transforming, acidic coiled-coil containing protein 2

NM_014810 centrosomal protein 350kDa

**Gene Group 15 Enrichment Score: 2.8662406271160514**

**REFSEQ_MRNA Gene Name**

NM_002035 3-ketodihydrosphingosine reductase

NM_006459 ER lipid raft associated 1

NM_021136 reticulon 1

NM_020438 dolichyl pyrophosphate phosphatase 1

NM_015161 ADP-ribosylation factor-like 6 interacting protein 1

NM_001004067 NODAL modulator 3; NODAL modulator 1; NODAL modulator 2

NM_006702 patatin-like phospholipase domain containing 6

NM_003144 signal sequence receptor, alpha

**Gene Group 16 Enrichment Score: 2.81542043764187**

**REFSEQ_MRNA Gene Name**

NM_001136126 cyclin D3

NM_001759 cyclin D2

NM_053056 cyclin D1

NM_001238, NM_057182 cyclin E1

**Gene Group 17 Enrichment Score: 2.4457171157270365**

**REFSEQ_MRNA Gene Name**

NM_033645 F-box and WD repeat domain containing 11

NM_017641 kinesin family member 21A

NM_017974 ATG16 autophagy related 16-like 1 (S. cerevisiae)

NM_004411 dynein, cytoplasmic 1, intermediate chain 1

NM_006141 dynein, cytoplasmic 1, light intermediate chain 2

NM_005509 Dmx-like 1

NM_014991 WD repeat and FYVE domain containing 3

NM_181291 WD repeat domain 20

NM_005112 WD repeat domain 1

**Gene Group 18 Enrichment Score: 1.949002252702314**

**REFSEQ_MRNA Gene Name**

NM_020860 stromal interaction molecule 2

NM_000388 calcium-sensing receptor

NM_020689 solute carrier family 24 (sodium/potassium/calcium exchanger), member 3

NM_002222 inositol 1,4,5-triphosphate receptor, type 1

NM_014849 synaptic vesicle glycoprotein 2A

NM_020655 junctophilin 3

**Gene Group 19 Enrichment Score: 1.7817666903746916**

**REFSEQ_MRNA Gene Name**

NM_005639 synaptotagmin I

NM_014849 synaptic vesicle glycoprotein 2A

NM_205848 synaptotagmin VI

NM_006754 synaptophysin-like 1

**Gene Group 20 Enrichment Score: 1.0637593110566945**

**REFSEQ_MRNA Gene Name**

NM_001004439 integrin, alpha 11

NM_002210 integrin, alpha V (vitronectin receptor, alpha polypeptide, antigen CD51)

NM_002205 integrin, alpha 5 (fibronectin receptor, alpha polypeptide)

NM_002203 integrin, alpha 2 (CD49B, alpha 2 subunit of VLA-2 receptor)

**Gene Group 21 Enrichment Score: 0.7420057786384043**

**REFSEQ_MRNA Gene Name**

NM_003654 carbohydrate (keratan sulfate Gal-6) sulfotransferase 1

NM_015879 ST8 alpha-N-acetyl-neuraminide alpha-2,8-sialyltransferase 3

NM_003896 ST3 beta-galactoside alpha-2,3-sialyltransferase 5

NM_001781 CD69 molecule

NM_017423 UDP-N-acetyl-alpha-D-galactosamine:polypeptide N-acetylgalactosaminyltransferase 7 (GalNAc-T7)

NM_020156 core 1 synthase, glycoprotein-N-acetylgalactosamine 3-beta-galactosyltransferase, 1

NM_004776 UDP-Gal:betaGlcNAc beta 1,4- galactosyltransferase, polypeptide 5

NM_032039 integrin alpha FG-GAP repeat containing 3

NM_002372 mannosidase, alpha, class 2A, member 1

**Gene Group 22 Enrichment Score: 0.5922611340389572**

**REFSEQ_MRNA Gene Name**

NM_032505 kelch repeat and BTB (POZ) domain containing 8

NM_006469 influenza virus NS1A binding protein

NM_014458 kelch-like 20 (Drosophila)

NM_015483 kelch repeat and BTB (POZ) domain containing 2

**Gene Group 23 Enrichment Score: 0.4985291161468318**

**REFSEQ_MRNA Gene Name**

NM_001304 carboxypeptidase D

NM_014243 ADAM metallopeptidase with thrombospondin type 1 motif, 3

NM_007038 ADAM metallopeptidase with thrombospondin type 1 motif, 5

NM_145243 OMA1 homolog, zinc metallopeptidase (S. cerevisiae)

NM_002581 PAPPA antisense RNA (non-protein coding); pregnancy-associated plasma protein A, pappalysin 1

NM_022353 O-sialoglycoprotein endopeptidase-like 1

NM_199355 ADAM metallopeptidase with thrombospondin type 1 motif, 18

**Gene Group 24 Enrichment Score: 0.48593103333936327**

**REFSEQ_MRNA Gene Name**

NM_001408 cadherin, EGF LAG seven-pass G-type receptor 2 (flamingo homolog, Drosophila)

NM_006016 CD164 molecule, sialomucin

NM_018907, NM_018900 protocadherin alpha 1; protocadherin alpha 4

NM_018908 protocadherin alpha 5

NM_015541 leucine-rich repeats and immunoglobulin-like domains 1

NM_003272 G protein-coupled receptor 137B

NM_000870 5-hydroxytryptamine (serotonin) receptor 4

NM_032039 integrin alpha FG-GAP repeat containing 3

NM_020403 protocadherin 9

NM_015236 latrophilin 3

NM_012302 latrophilin 2

NM_012428 neuroplastin

NM_001003674 chromosome 18 open reading frame 1

NM_003144 signal sequence receptor, alpha

NM_153685 chromosome 12 open reading frame 53

NM_012092 inducible T-cell co-stimulator

NM_001781 CD69 molecule

NM_052880 phosphoinositide-3-kinase interacting protein 1

NM_020156 core 1 synthase, glycoprotein-N-acetylgalactosamine 3-beta-galactosyltransferase, 1

NM_012104 beta-site APP-cleaving enzyme 1

NM_018901, NM_018904, NM_018898, NM_018899 protocadherin alpha 13; protocadherin alpha 10; protocadherin alpha subfamily C, 1; protocadherin alpha subfamily C, 2

NM_001004439 integrin, alpha 11

NM_000722 calcium channel, voltage-dependent, alpha 2/delta subunit 1

NM_000891 potassium inwardly-rectifying channel, subfamily J, member 2

NM_153619 sema domain, transmembrane domain (TM), and cytoplasmic domain, (semaphorin) 6D

NM_002233 potassium voltage-gated channel, shaker-related subfamily, member 4

NM_012342 hypothetical LOC729590; BMP and activin membrane-bound inhibitor homolog (Xenopus laevis)

NM_001046 solute carrier family 12 (sodium/potassium/chloride transporters), member 2

NM_000958 prostaglandin E receptor 4 (subtype EP4)

NM_032961 protocadherin 10

NM_014271 interleukin 1 receptor accessory protein-like 1

NM_032973 protocadherin 11 Y-linked

NM_018905 protocadherin alpha 2

NM_031418 anoctamin 3

NM_012129 claudin 12

NM_003759 solute carrier family 4, sodium bicarbonate cotransporter, member 4

NM_006749 solute carrier family 20 (phosphate transporter), member 2

NM_018909 protocadherin alpha 8; protocadherin alpha 6

NM_015234 G protein-coupled receptor 116

NM_020689 solute carrier family 24 (sodium/potassium/calcium exchanger), member 3

NM_020708 solute carrier family 12 (potassium-chloride transporter), member 5

NM_021572 ectonucleotide pyrophosphatase/phosphodiesterase 5 (putative function)

NM_018043 anoctamin 1, calcium activated chloride channel

NM_018906 protocadherin alpha 3

NM_004776 UDP-Gal:betaGlcNAc beta 1,4- galactosyltransferase, polypeptide 5

NM_001004067 NODAL modulator 3; NODAL modulator 1; NODAL modulator 2

NM_018910 protocadherin alpha 7

NM_182527 calcium binding protein 7

**Gene Group 25 Enrichment Score: 0.39124110817869695**

**REFSEQ_MRNA Gene Name**

NM_021814 ELOVL family member 5, elongation of long chain fatty acids (FEN1/Elo2, SUR4/Elo3-like, yeast)

NM_003272 G protein-coupled receptor 137B

NM_144599 non imprinted in Prader-Willi/Angelman syndrome 1

NM_024293 family with sequence similarity 134, member A

NM_032039 integrin alpha FG-GAP repeat containing 3

NM_005779 lipoma HMGIC fusion partner-like 2

NM_014283 chromosome 1 open reading frame 9

NM_017851 poly (ADP-ribose) polymerase family, member 16

NM_138459 nuclear undecaprenyl pyrophosphate synthase 1 pseudogene; nuclear undecaprenyl pyrophosphate synthase 1 homolog (S. cerevisiae)

NM_182511 cerebellin 2 precursor

NM_001003674 chromosome 18 open reading frame 1

NM_018710 transmembrane protein 55A

NM_001006605 family with sequence similarity 69, member A

NM_153261 transmembrane protein 188; similar to TMEM188 protein

NM_153685 chromosome 12 open reading frame 53

NM_012092 inducible T-cell co-stimulator

NM_052880 phosphoinositide-3-kinase interacting protein 1

NM_021136 reticulon 1

NM_180989 G protein-coupled receptor 180

NM_138771 coiled-coil domain containing 126

NM_006134 transmembrane protein 50B

NM_015497 transmembrane protein 87A

NM_017938 family with sequence similarity 70, member A

NM_024759 NIPA-like domain containing 2

NM_005277 glycoprotein M6A

NM_198549 family with sequence similarity 73, member A

NM_012342 hypothetical LOC729590; BMP and activin membrane-bound inhibitor homolog (Xenopus laevis)

NM_018126 transmembrane protein 33

NM_001001330 receptor accessory protein 3

NM_032181 family with sequence similarity 176, member A

NM_020182 prostate transmembrane protein, androgen induced 1

NM_016072 golgi transport 1 homolog B (S. cerevisiae)

NM_017786 Golgi-localized protein

NM_015676 chromosome 14 open reading frame 109

NM_016097 immediate early response 3 interacting protein 1

NM_015948 solute carrier family 35, member B3

NM_153226 transmembrane protein 20

NM_025179 plexin A2

NM_032160 dermatan sulfate epimerase-like

NM_022373 HERPUD family member 2

NM_019556 motile sperm domain containing 1

NM_021572 ectonucleotide pyrophosphatase/phosphodiesterase 5 (putative function)

NM_017753 plasticity related gene 3

NM_032312 Yip1 domain family, member 4

NM_001004360 dipeptidyl-peptidase 10

NM_017941 chromosome 17 open reading frame 80

NM_001004067 NODAL modulator 3; NODAL modulator 1; NODAL modulator 2

NM_017994 chromosome 7 open reading frame 42

NM_014056 similar to HIG1 domain family, member 1A; HIG1 hypoxia inducible domain family, member 1A; HIG1 hypoxia inducible domain family, member 1D

NM_080546 solute carrier family 44, member 1

NM_032859 abhydrolase domain containing 13

NM_003498 stannin

NM_020644 TMEM9 domain family, member B

**Gene Group 26 Enrichment Score: 0.30487989773962193**

**REFSEQ_MRNA Gene Name**

NM_020116 follistatin-like 5

NM_182527 calcium binding protein 7

NM_022138 SPARC related modular calcium binding 2

NM_207517 ADAMTS-like 3

**Arresting gd20 Endometrium > Arresting gd20 Trophoblast**

**Gene Functional Classification Result**

**Gene Group 1 Enrichment Score: 6.243917853529751**

**REFSEQ_MRNA Gene Name**

NM_021145 cyclin D binding myb-like transcription factor 1

NM_152622 mesoderm induction early response 1, family member 3

NM_014667 vestigial like 4 (Drosophila)

NM_015608 chromosome 10 open reading frame 137

NM_006874 E74-like factor 2 (ets domain transcription factor)

**Gene Group 2 Enrichment Score: 6.118815825370912**

**REFSEQ_MRNA Gene Name**

NM_000393 collagen, type V, alpha 2

NM_001844 collagen, type II, alpha 1

NM_000094 collagen, type VII, alpha 1

NM_001854 collagen, type XI, alpha 1

NM_004370 collagen, type XII, alpha 1

NM_152890 collagen, type XXIV, alpha 1

NM_001858 collagen, type XIX, alpha 1

NM_015719 collagen, type V, alpha 3

NM_000091 collagen, type IV, alpha 3 (Goodpasture antigen)

NM_001855 collagen, type XV, alpha 1

NM_000088 collagen, type I, alpha 1

NM_000089 collagen, type I, alpha 2

NM_004369 collagen, type VI, alpha 3

NM_001845 collagen, type IV, alpha 1

NM_000092 collagen, type IV, alpha 4

NM_001846 collagen, type IV, alpha 2

NM_001851 collagen, type IX, alpha 1

NM_000090 collagen, type III, alpha 1

**Gene Group 3 Enrichment Score: 4.490497117232026**

**REFSEQ_MRNA Gene Name**

NM_006079 Cbp/p300-interacting transactivator, with Glu/Asp-rich carboxy-terminal domain, 2

NM_001755 core-binding factor, beta subunit

NM_005595 nuclear factor I/A

NM_172238 transcription factor AP-2 delta (activating enhancer binding protein 2 delta)

NM_006951 TAF5 RNA polymerase II, TATA box binding protein (TBP)-associated factor, 100kDa

NM_006015 AT rich interactive domain 1A (SWI-like)

NM_003074 SWI/SNF related, matrix associated, actin dependent regulator of chromatin, subfamily c, member 1

NM_004229 mediator complex subunit 14

NM_199072 MyoD family inhibitor domain containing

NM_012082 zinc finger protein, multitype 2

NM_001621 aryl hydrocarbon receptor

NM_012234 RING1 and YY1 binding protein

NM_021813 BTB and CNC homology 1, basic leucine zipper transcription factor 2

NM_021961 TEA domain family member 1 (SV40 transcriptional enhancer factor)

NM_022462 hypoxia inducible factor 3, alpha subunit

NM_004379 cAMP responsive element binding protein 1

NM_018951 homeobox A10

NM_000346 SRY (sex determining region Y)-box 9

NM_032329 inhibitor of growth family, member 5

NM_003222 transcription factor AP-2 gamma (activating enhancer binding protein 2 gamma)

NM_024501 homeobox D1

NM_015138 Rtf1, Paf1/RNA polymerase II complex component, homolog (S. cerevisiae)

NM_001001890 runt-related transcription factor 1

NM_004821 heart and neural crest derivatives expressed 1

NM_006791 mortality factor 4; mortality factor 4 like 1

NM_003822 nuclear receptor subfamily 5, group A, member 2

NM_001949 E2F transcription factor 3

NM_153607 chromosome 5 open reading frame 41

NM_057175 NMDA receptor regulated 1

NM_021145 cyclin D binding myb-like transcription factor 1

NM_012081 elongation factor, RNA polymerase II, 2

NM_053002 mediator complex subunit 12-like

NM_004449 v-ets erythroblastosis virus E26 oncogene homolog (avian)

NM_006874 E74-like factor 2 (ets domain transcription factor)

NM_022728 neurogenic differentiation 6

NM_032440 ligand dependent nuclear receptor corepressor

NM_021809 TGFB-induced factor homeobox 2

NM_001186 BTB and CNC homology 1, basic leucine zipper transcription factor 1

NM_018416 forkhead box J2

NM_006164 nuclear factor (erythroid-derived 2)-like 2

NM_006195 pre-B-cell leukemia homeobox 3

NM_000192 T-box 5

NM_021240 doublesex and mab-3 related transcription factor 3

NM_007162 transcription factor EB

NM_003112 Sp4 transcription factor

NM_003205 transcription factor 12

**Gene Group 4 Enrichment Score: 3.133956166222136**

**REFSEQ_MRNA Gene Name**

NM_018211 ribonucleoprotein, PTB-binding 2

NM_153020 RNA binding motif protein 24

NM_006547 insulin-like growth factor 2 mRNA binding protein 3

NM_182485 cytoplasmic polyadenylation element binding protein 2

**Gene Group 5 Enrichment Score: 3.0876403289519176**

**REFSEQ_MRNA Gene Name**

NM_172238 transcription factor AP-2 delta (activating enhancer binding protein 2 delta)

NM_014795 zinc finger E-box binding homeobox 2

NM_015534 zinc finger, ZZ-type containing 3

NM_012082 zinc finger protein, multitype 2

NM_012234 RING1 and YY1 binding protein

NM_004349 runt-related transcription factor 1; translocated to, 1 (cyclin D-related)

NM_003575 zinc finger protein 282

NM_017544 NFKB repressing factor

NM_003111 Sp3 transcription factor

NM_012421 rearranged L-myc fusion

NM_005234 nuclear receptor subfamily 2, group F, member 6

NM_015094 hypermethylated in cancer 2

NM_023929 zinc finger and BTB domain containing 10

NM_004926 zinc finger protein 36, C3H type-like 1

NM_015138 Rtf1, Paf1/RNA polymerase II complex component, homolog (S. cerevisiae)

NM_005180 BMI1 polycomb ring finger oncogene

NM_004821 heart and neural crest derivatives expressed 1

NM_014491 forkhead box P2

NM_003822 nuclear receptor subfamily 5, group A, member 2

NM_182907 PR domain containing 1, with ZNF domain

NM_002655 pleiomorphic adenoma gene 1

NM_015481 zinc finger protein 385A

NM_005392 PHD finger protein 2

NM_032440 ligand dependent nuclear receptor corepressor

NM_022781 ring finger protein 38

NM_001186 BTB and CNC homology 1, basic leucine zipper transcription factor 1

NM_152493 zinc finger protein 362

NM_003453 zinc finger, MYM-type 2

NM_017742 zinc finger, CCHC domain containing 2

NM_022552 DNA (cytosine-5-)-methyltransferase 3 alpha

NM_022893 B-cell CLL/lymphoma 11A (zinc finger protein)

NM_021240 doublesex and mab-3 related transcription factor 3

NM_007162 transcription factor EB

NM_003112 Sp4 transcription factor

NM_138473 Sp1 transcription factor

NM_004241 jumonji domain containing 1C

NM_001991 enhancer of zeste homolog 1 (Drosophila)

NM_152478 zinc finger protein 583

**Gene Group 6 Enrichment Score: 2.651576647099595**

**REFSEQ_MRNA Gene Name**

NM_015271 tripartite motif-containing 2

NM_016271 ring finger protein 138

NM_017742 zinc finger, CCHC domain containing 2

NM_005180 BMI1 polycomb ring finger oncogene

NM_152493 zinc finger protein 362

NM_023929 zinc finger and BTB domain containing 10

NM_030576 LIM domain containing 2

NM_017610 ring finger protein 111

NM_152896 ubiquitin-like with PHD and ring finger domains 2

NM_005392 PHD finger protein 2

NM_015457 zinc finger, DHHC-type containing 5

NM_001039111 tripartite motif-containing 71

NM_033089 zinc finger, CCHC domain containing 3

NM_003575 zinc finger protein 282

NM_004241 jumonji domain containing 1C

NM_172070 ubiquitin protein ligase E3 component n-recognin 3 (putative)

NM_030625 tet oncogene 1

NM_001002909 G patch domain containing 8

NM_182765 HECT domain containing 2

NM_015534 zinc finger, ZZ-type containing 3

NM_015435 ring finger protein 19A

NM_015481 zinc finger protein 385A

NM_021240 doublesex and mab-3 related transcription factor 3

NM_152787 mitogen-activated protein kinase kinase kinase 7 interacting protein 3

NM_022781 ring finger protein 38

NM_015346 zinc finger, FYVE domain containing 26

NM_013262 myosin regulatory light chain interacting protein

**Gene Group 7 Enrichment Score: 2.3903654605275655**

**REFSEQ_MRNA Gene Name**

NM_003794 sorting nexin 4

NM_030918 sorting nexin family member 27

NM_022133 sorting nexin 16

NM_014035 sorting nexin 24

**Gene Group 8 Enrichment Score: 2.357096549900224**

**REFSEQ_MRNA Gene Name**

NM_006048 ubiquitination factor E4B (UFD2 homolog, yeast)

NM_004359 cell division cycle 34 homolog (S. cerevisiae)

NM_182765 HECT domain containing 2

NM_017582 ubiquitin-conjugating enzyme E2Q family member 1

NM_007013 WW domain containing E3 ubiquitin protein ligase 1

**Gene Group 9 Enrichment Score: 2.2575086629082204**

**REFSEQ_MRNA Gene Name**

NM_003390 WEE1 homolog (S. pombe)

NM_152835 PDLIM1 interacting kinase 1 like

NM_032430 BR serine/threonine kinase 1

NM_018571 STE20-related kinase adaptor beta

NM_017553 INO80 homolog (S. cerevisiae)

NM_003010 mitogen-activated protein kinase kinase 4

NM_021643 tribbles homolog 2 (Drosophila)

NM_198465 Nik related kinase

NM_014840 NUAK family, SNF1-like kinase, 1

NM_005627 serum/glucocorticoid regulated kinase 1

NM_004734 doublecortin-like kinase 1

NM_014397 NIMA (never in mitosis gene a)-related kinase 6

NM_003831 RIO kinase 3 (yeast)

NM_002314 LIM domain kinase 1

NM_032017 serine/threonine kinase 40

NM_003618 mitogen-activated protein kinase kinase kinase kinase 3

NM_002595 PCTAIRE protein kinase 2

NM_001259 cyclin-dependent kinase 6

NM_001222 calcium/calmodulin-dependent protein kinase II gamma

NM_145259 activin A receptor, type IC

NM_173797 PAP associated domain containing 4

NM_005433 v-yes-1 Yamaguchi sarcoma viral oncogene homolog 1

NM_006852 tousled-like kinase 2

NM_014572 LATS, large tumor suppressor, homolog 2 (Drosophila)

**Gene Group 10 Enrichment Score: 2.2030209327665333**

**REFSEQ_MRNA Gene Name**

NM_153810 chromosome 10 open reading frame 46

NM_020640 DCN1, defective in cullin neddylation 1, domain containing 1 (S. cerevisiae)

NM_080867 suppressor of cytokine signaling 4

NM_013396 ubiquitin specific peptidase 25

NM_014871 PAN2 poly(A) specific ribonuclease subunit homolog (S. cerevisiae)

NM_006313 ubiquitin specific peptidase 15

NM_198243 ankyrin repeat and SOCS box-containing 7

NM_022832 ubiquitin specific peptidase 46

NM_015626 WD repeat and SOCS box-containing 1

NM_182765 HECT domain containing 2

NM_032557 ubiquitin specific peptidase 38

NM_080862 splA/ryanodine receptor domain and SOCS box containing 4

NM_018315 F-box and WD repeat domain containing 7

NM_004232 suppressor of cytokine signaling 6

**Gene Group 11 Enrichment Score: 1.9544140146760025**

**REFSEQ_MRNA Gene Name**

NM_014397 NIMA (never in mitosis gene a)-related kinase 6

NM_004856 kinesin family member 23

NM_015032 PDS5, regulator of cohesion maintenance, homolog B (S. cerevisiae)

NM_006306 structural maintenance of chromosomes 1A

**Gene Group 12 Enrichment Score: 1.54575671738661**

**REFSEQ_MRNA Gene Name**

NM_198935 synovial sarcoma translocation gene on chromosome 18-like 1

NM_017635 suppressor of variegation 4-20 homolog 1 (Drosophila)

NM_001991 enhancer of zeste homolog 1 (Drosophila)

NM_032701 suppressor of variegation 4-20 homolog 2 (Drosophila)

**Gene Group 13 Enrichment Score: 1.5290754366530497**

**REFSEQ_MRNA Gene Name**

NM_004411 dynein, cytoplasmic 1, intermediate chain 1

NM_015626 WD repeat and SOCS box-containing 1

NM_017641 kinesin family member 21A

NM_153252 bromodomain and WD repeat domain containing 3

NM_019063 echinoderm microtubule associated protein like 4

NM_018315 F-box and WD repeat domain containing 7

NM_003939 beta-transducin repeat containing

**Gene Group 14 Enrichment Score: 1.525440648107985**

**REFSEQ_MRNA Gene Name**

NM_006924 splicing factor, arginine/serine-rich 1

NM_054016 FUS interacting protein (serine/arginine-rich) 1; similar to FUS interacting protein (serine-arginine rich) 1

NM_018061 PRP38 pre-mRNA processing factor 38 (yeast) domain containing B

NM_016312 WW domain binding protein 11

**Gene Group 15 Enrichment Score: 1.432515792699438**

**REFSEQ_MRNA Gene Name**

NM_020159 SWI/SNF-related, matrix-associated actin-dependent regulator of chromatin, subfamily a, containing DEAD/H box 1

NM_198963 DEAH (Asp-Glu-Ala-Asp/His) box polypeptide 57

NM_018332 DEAD (Asp-Glu-Ala-As) box polypeptide 19A

NM_017553 INO80 homolog (S. cerevisiae)

**Gene Group 16 Enrichment Score: 0.8545713939165032**

**REFSEQ_MRNA Gene Name**

NM_001660 ADP-ribosylation factor 4

NM_016322 RAB14, member RAS oncogene family

NM_021183 RAP2C, member of RAS oncogene family

NM_016131 RAB10, member RAS oncogene family

NM_016544 DnaJ (Hsp40) homolog, subfamily C, member 27

**Gene Group 17 Enrichment Score: 0.7499760001965879**

**REFSEQ_MRNA Gene Name**

NM_006310 hypothetical protein FLJ11822; aminopeptidase puromycin sensitive

NM_182920 ADAM metallopeptidase with thrombospondin type 1 motif, 9

NM_007038 ADAM metallopeptidase with thrombospondin type 1 motif, 5

NM_002581 PAPPA antisense RNA (non-protein coding); pregnancy-associated plasma protein A, pappalysin 1

**Gene Group 18 Enrichment Score: 0.2987349674222878**

**REFSEQ_MRNA Gene Name**

NM_153377 leucine-rich repeats and immunoglobulin-like domains 3

NM_052910 SLIT and NTRK-like family, member 1

NM_002998 syndecan 2

NM_031418 anoctamin 3

NM_005302 G protein-coupled receptor 37 (endothelin receptor type B-like)

NM_139177 solute carrier family 39 (metal ion transporter), member 11

NM_018910 protocadherin alpha 7

NM_012129 claudin 12

NM_018906 protocadherin alpha 3

NM_014813 leucine-rich repeats and immunoglobulin-like domains 2

NM_013281 fibronectin leucine rich transmembrane protein 3

NM_012302 latrophilin 2

NM_003759 solute carrier family 4, sodium bicarbonate cotransporter, member 4

NM_018908 protocadherin alpha 5

NM_018905 protocadherin alpha 2

NM_018490 leucine-rich repeat-containing G protein-coupled receptor 4

NM_138440 vasorin

NM_016548 golgi membrane protein 1

NM_018898, NM_018901, NM_018904, NM_018899 protocadherin alpha 13; protocadherin alpha 10; protocadherin alpha subfamily C, 1; protocadherin alpha subfamily C, 2

NM_032229 SLIT and NTRK-like family, member 6

NM_000958 prostaglandin E receptor 4 (subtype EP4)

NM_018903 protocadherin alpha 12

NM_005797 myelin protein zero-like 2

NM_001795 cadherin 5, type 2 (vascular endothelium)

NM_000870 5-hydroxytryptamine (serotonin) receptor 4

NM_152390 transmembrane protein 178

NM_020925 cache domain containing 1

NM_020403 protocadherin 9

NM_018909, NM_018911 protocadherin alpha 8; protocadherin alpha 6

NM_012329 monocyte to macrophage differentiation-associated

NM_153619 sema domain, transmembrane domain (TM), and cytoplasmic domain, (semaphorin) 6D

NM_005927 microfibrillar-associated protein 3

NM_018907, NM_018900 protocadherin alpha 1; protocadherin alpha 4

NM_005329 hyaluronan synthase 3

NM_006016 CD164 molecule, sialomucin

**Gene Group 19 Enrichment Score: 0.04164847992338618**

**REFSEQ_MRNA Gene Name**

NM_017786 Golgi-localized protein

NM_002998 syndecan 2

NM_138391 transmembrane protein 183A; transmembrane protein 183B

NM_018126 transmembrane protein 33

NM_153365 transmembrane anterior posterior transformation 1

NM_014918 chondroitin sulfate synthase 1

NM_152400 chromosome 4 open reading frame 32

NM_006754 synaptophysin-like 1

NM_152996 ST6 (alpha-N-acetyl-neuraminyl-2,3-beta-galactosyl-1,3)-N-acetylgalactosaminide alpha-2,6-sialyltransferase 3

NM_003759 solute carrier family 4, sodium bicarbonate cotransporter, member 4

NM_032859 abhydrolase domain containing 13

NM_031434 transmembrane and ubiquitin-like domain containing 1

NM_013390 transmembrane protein 2

NM_025076 UDP-glucuronate decarboxylase 1

NM_014056 similar to HIG1 domain family, member 1A; HIG1 hypoxia inducible domain family, member 1A; HIG1 hypoxia inducible domain family, member 1D

NM_032947 MSTP150

NM_016548 golgi membrane protein 1

NM_138390 transmembrane protein 169

NM_017423 UDP-N-acetyl-alpha-D-galactosamine:polypeptide N-acetylgalactosaminyltransferase 7 (GalNAc-T7)

NM_003779 UDP-Gal:betaGlcNAc beta 1,4- galactosyltransferase, polypeptide 3

NM_017938 family with sequence similarity 70, member A

NM_014283 chromosome 1 open reading frame 9

NM_152390 transmembrane protein 178

NM_016591 glucosaminyl (N-acetyl) transferase 4, core 2 (beta-1,6-N-acetylglucosaminyltransferase)

NM_015008 transmembrane and coiled-coil domain family 1

NM_007001 solute carrier family 35, member D2

NM_018710 transmembrane protein 55A

NM_153226 transmembrane protein 20

NM_005415 solute carrier family 20 (phosphate transporter), member 1

NM_152527 solute carrier family 16, member 14 (monocarboxylic acid transporter 14)

NM_005927 microfibrillar-associated protein 3

NM_005329 hyaluronan synthase 3

NM_173683 XK, Kell blood group complex subunit-related family, member 6

**Arresting gd20 Trophoblast > Arresting gd20 Endometrium**

**Gene Functional Classification Result**

**Gene Group 1 Enrichment Score: 3.9724556670317686**

**REFSEQ_MRNA Gene Name**

NM_016169 suppressor of fused homolog (Drosophila)

NM_032458 PHD finger protein 6

NM_020382 SET domain containing (lysine methyltransferase) 8

NM_014795 zinc finger E-box binding homeobox 2

NM_030762 basic helix-loop-helix family, member e41

NM_018263 additional sex combs like 2 (Drosophila)

NM_003074 SWI/SNF related, matrix associated, actin dependent regulator of chromatin, subfamily c, member 1

NM_012082 zinc finger protein, multitype 2

NM_199072 MyoD family inhibitor domain containing

NM_012234 RING1 and YY1 binding protein

NM_004349 runt-related transcription factor 1; translocated to, 1 (cyclin D-related)

NM_021961 TEA domain family member 1 (SV40 transcriptional enhancer factor)

NM_015035 zinc fingers and homeoboxes 3

NM_001452 forkhead box F2

NM_203394 E2F transcription factor 7

NM_003107 SRY (sex determining region Y)-box 4

NM_015094 hypermethylated in cancer 2

NM_004235 Kruppel-like factor 4 (gut)

NM_002968 sal-like 1 (Drosophila)

NM_006352 zinc finger protein 238

NM_004293 guanine deaminase

NM_014682 suppression of tumorigenicity 18 (breast carcinoma) (zinc finger protein)

NM_003222 transcription factor AP-2 gamma (activating enhancer binding protein 2 gamma)

NM_002938 ring finger protein 4; hypothetical LOC644006

NM_013450 bromodomain adjacent to zinc finger domain, 2B

NM_014552 grainyhead-like 1 (Drosophila)

NM_003216 thyrotrophic embryonic factor

NM_004535 myelin transcription factor 1

NM_057175 NMDA receptor regulated 1

NM_021620 PR domain containing 13

NM_001002909 G patch domain containing 8

NM_153450 mediator complex subunit 19

NM_002687 pinin, desmosome associated protein

NM_002655 pleiomorphic adenoma gene 1

NM_007375 TAR DNA binding protein

NM_021943 zinc finger, AN1-type domain 3

NM_006734 human immunodeficiency virus type I enhancer binding protein 2

NM_005230 ELK3, ETS-domain protein (SRF accessory protein 2)

NM_016436 PHD finger protein 20

NM_013449 bromodomain adjacent to zinc finger domain, 2A

NM_053002 mediator complex subunit 12-like

NM_014945 actin binding LIM protein family, member 3

NM_032440 ligand dependent nuclear receptor corepressor

NM_022781 ring finger protein 38

NM_003453 zinc finger, MYM-type 2

NM_017519 AT rich interactive domain 1B (SWI1-like)

NM_022893 B-cell CLL/lymphoma 11A (zinc finger protein)

NM_017778 Wolf-Hirschhorn syndrome candidate 1-like 1

NM_018200 high-mobility group 20A

NM_033224 purine-rich element binding protein B

NM_005461 v-maf musculoaponeurotic fibrosarcoma oncogene homolog B (avian)

NM_004992 methyl CpG binding protein 2 (Rett syndrome)

NM_001001484 phosphotriesterase related

**Gene Group 2 Enrichment Score: 2.8932783221293494**

**REFSEQ_MRNA Gene Name**

NM_003390 WEE1 homolog (S. pombe)

NM_173354 salt-inducible kinase 1

NM_014683 unc-51-like kinase 2 (C. elegans)

NM_017553 INO80 homolog (S. cerevisiae)

NM_003010 mitogen-activated protein kinase kinase 4

NM_015216 histidine acid phosphatase domain containing 1

NM_012395 PFTAIRE protein kinase 1

NM_004440 EPH receptor A7

NM_002648 pim-1 oncogene

NM_006141 dynein, cytoplasmic 1, light intermediate chain 2

NM_005813 protein kinase D3

NM_001259 cyclin-dependent kinase 6

NM_017719 SNF related kinase

NM_175854 PAN3 poly(A) specific ribonuclease subunit homolog (S. cerevisiae)

NM_015076 cell division cycle 2-like 6 (CDK8-like)

NM_016308 cytidine monophosphate (UMP-CMP) kinase 1, cytosolic

NM_016231 nemo-like kinase

NM_014572 LATS, large tumor suppressor, homolog 2 (Drosophila)

**Gene Group 3 Enrichment Score: 2.7184273862773933**

**REFSEQ_MRNA Gene Name**

NM_004856 kinesin family member 23

NM_004984 kinesin family member 5A

NM_017641 kinesin family member 21A

NM_012223 myosin IB

NM_006141 dynein, cytoplasmic 1, light intermediate chain 2

NM_004521 kinesin family member 5B

**Gene Group 4 Enrichment Score: 2.3331095170554126**

**REFSEQ_MRNA Gene Name**

NM_025134 chromodomain helicase DNA binding protein 9

NM_000489 alpha thalassemia/mental retardation syndrome X-linked (RAD54 homolog, S. cerevisiae)

NM_003972 BTAF1 RNA polymerase II, B-TFIID transcription factor-associated, 170kDa (Mot1 homolog, S. cerevisiae)

NM_017553 INO80 homolog (S. cerevisiae)

**Gene Group 5 Enrichment Score: 2.2358155748569457**

**REFSEQ_MRNA Gene Name**

NM_016322 RAB14, member RAS oncogene family

NM_016277 RAB23, member RAS oncogene family

NM_016131 RAB10, member RAS oncogene family

NM_004162 RAB5A, member RAS oncogene family

NM_014488 RAB30, member RAS oncogene family

NM_031934 RAB34, member RAS oncogene family

NM_198686 RAB15, member RAS onocogene family

**Gene Group 6 Enrichment Score: 1.4165350802318455**

**REFSEQ_MRNA Gene Name**

NM_005509 Dmx-like 1

NM_017641 kinesin family member 21A

NM_033645 F-box and WD repeat domain containing 11

NM_181291 WD repeat domain 20

**Gene Group 7 Enrichment Score: 1.3334202798741515**

**REFSEQ_MRNA Gene Name**

NM_020975 ret proto-oncogene

NM_004440 EPH receptor A7

NM_001204 bone morphogenetic protein receptor, type II (serine/threonine kinase)

NM_001105 activin A receptor, type I

**Gene Group 8 Enrichment Score: 0.6166892335473969**

**REFSEQ_MRNA Gene Name**

NM_004776 UDP-Gal:betaGlcNAc beta 1,4- galactosyltransferase, polypeptide 5

NM_017423 UDP-N-acetyl-alpha-D-galactosamine:polypeptide N-acetylgalactosaminyltransferase 7 (GalNAc-T7)

NM_015879 ST8 alpha-N-acetyl-neuraminide alpha-2,8-sialyltransferase 3

NM_002372 mannosidase, alpha, class 2A, member 1

**Gene Group 9 Enrichment Score: 0.062289024997503606**

**REFSEQ_MRNA Gene Name**

NM_017994 chromosome 7 open reading frame 42

NM_080546 solute carrier family 44, member 1

NM_001781 CD69 molecule

NM_001004067 NODAL modulator 3; NODAL modulator 1; NODAL modulator 2

NM_021136 reticulon 1

NM_005779 lipoma HMGIC fusion partner-like 2

NM_015236 latrophilin 3

NM_024293 family with sequence similarity 134, member A

NM_015497 transmembrane protein 87A

NM_003498 stannin

NM_001003674 chromosome 18 open reading frame 1

NM_000958 prostaglandin E receptor 4 (subtype EP4)

NM_016097 immediate early response 3 interacting protein 1

NM_153261 transmembrane protein 188; similar to TMEM188 protein

NM_006134 transmembrane protein 50B

NM_032312 Yip1 domain family, member 4

NM_198549 family with sequence similarity 73, member A

NM_032039 integrin alpha FG-GAP repeat containing 3

NM_180989 G protein-coupled receptor 180

NM_032181 family with sequence similarity 176, member A

NM_052880 phosphoinositide-3-kinase interacting protein 1

NM_032973 protocadherin 11 Y-linked

NM_153226 transmembrane protein 20

NM_022373 HERPUD family member 2

NM_017851 poly (ADP-ribose) polymerase family, member 16

NM_012092 inducible T-cell co-stimulator

NM_001004360 dipeptidyl-peptidase 10

**Healthy gd20 Endometrium > Arresting gd20 Endometrium**

**Gene Functional Classification Result**

**Gene Group 1 Enrichment Score: 1.6352336901617288**

**REFSEQ_MRNA Gene Name**

NM_145872 ankyrin repeat and SOCS box-containing 4

NM_001002255 SMT3 suppressor of mif two 3 homolog 4 (S. cerevisiae)

NM_174899 F-box protein 36

NM_013396 ubiquitin specific peptidase 25

NM_001005849 SMT3 suppressor of mif two 3 homolog 2 (S. cerevisiae) pseudogene; SMT3 suppressor of mif two 3 homolog 2 (S. cerevisiae); SMT3 suppressor of mif two 3 homolog 3 (S. cerevisiae)

NM_015246 mahogunin, ring finger 1

NM_183415, NM_130466 ubiquitin protein ligase E3B

NM_018227 ubiquitin-like modifier activating enzyme 6

**Gene Group 2 Enrichment Score: 1.4190670548234738**

**REFSEQ_MRNA Gene Name**

NM_001031623 zinc finger protein 451

NM_001039649 zinc finger, MYM-type 5

NM_173570 zinc finger, DHHC-type containing 23

NM_024672 THAP domain containing 9

NM_203406 metallo-beta-lactamase domain containing 2

NM_015246 mahogunin, ring finger 1

NM_001001894, NM_003316 tetratricopeptide repeat domain 3; tetratricopeptide repeat domain 3-like

NM_013316 CCR4-NOT transcription complex, subunit 4

NM_153371 ligand of numb-protein X 2

NM_181846 zinc finger and SCAN domain containing 22

NM_025188 tripartite motif-containing 45

NM_021951 doublesex and mab-3 related transcription factor 1

NM_001024593 zinc finger, MYND-type containing 17

NM_001003818, NM_058166 TRIM6-TRIM34 readthrough transcript; tripartite motif-containing 6; tripartite motif-containing 34

NM_003430 zinc finger protein 91

NM_198581 zinc finger CCCH-type containing 6

NM_152437 zinc finger protein 664

NM_014240 LIM domains containing 1

NM_183238 similar to ZNF605 protein

NM_005667 vacuolar protein sorting 24 homolog (S. cerevisiae); ring finger protein 103

NM_014910 zinc finger protein 507

NM_001077195 zinc finger protein 436

NM_017656 zinc finger protein 562

NM_004234 zinc finger protein 235

NM_145796 pogo transposable element with ZNF domain

NM_001013691 zinc finger protein 833

NM_173084 tripartite motif-containing 59

NM_057178 ring finger and FYVE-like domain containing 1

NM_032018 chromosome 1 open reading frame 124

NM_144684 zinc finger protein 480

NM_001012756 zinc finger protein 260

NM_182898, NM_182899, NM_001011666, NM_004904 cAMP responsive element binding protein 5

NM_019083 coiled-coil domain containing 76

NM_019006 zinc finger, AN1-type domain 6

NM_138494, NM_001009960 zinc finger protein 655

**Gene Group 3 Enrichment Score: 1.3219379582525412**

**REFSEQ_MRNA Gene Name**

NM_178007, NM_052851, NM_178006 StAR-related lipid transfer (START) domain containing 13

NM_032900 Rho GTPase activating protein 19

NM_001033117, NM_014850 SLIT-ROBO Rho GTPase activating protein 3

NM_014859 Rho-type GTPase-activating protein RICH2

NM_018948 ERBB receptor feedback inhibitor 1

NM_016603 family with sequence similarity 13, member B

**Gene Group 4 Enrichment Score: 0.9568280026105129**

**REFSEQ_MRNA Gene Name**

NM_013382 protein-O-mannosyltransferase 2

NM_006581 fucosyltransferase 9 (alpha (1,3) fucosyltransferase)

NM_015879 ST8 alpha-N-acetyl-neuraminide alpha-2,8-sialyltransferase 3

NM_012214 mannosyl (alpha-1,3-)-glycoprotein beta-1,4-N-acetylglucosaminyltransferase, isozyme A

NM_001079802, NM_006731 fukutin

NM_052917 UDP-N-acetyl-alpha-D-galactosamine:polypeptide N-acetylgalactosaminyltransferase 13 (GalNAc-T13); UDP-N-acetyl-alpha-D-galactosamine:polypeptide N-acetylgalactosaminyltransferase 1 (GalNAc-T1)

**Gene Group 5 Enrichment Score: 0.6428060941970324**

**REFSEQ_MRNA Gene Name**

NM_001046 solute carrier family 12 (sodium/potassium/chloride transporters), member 2

NM_133478 solute carrier family 4, sodium bicarbonate cotransporter, member 5

NM_006359, NM_001042537 solute carrier family 9 (sodium/hydrogen exchanger), member 6

NM_006933 solute carrier family 5 (sodium/myo-inositol cotransporter), member 3

**Gene Group 6 Enrichment Score: 0.44400929279072837**

**REFSEQ_MRNA Gene Name**

NM_001274 CHK1 checkpoint homolog (S. pombe)

NM_006609 mitogen-activated protein kinase kinase kinase 2

NM_014920 intestinal cell (MAK-like) kinase

NM_004384, NM_001031812, NM_001044723 casein kinase 1, gamma 3

**Gene Group 7 Enrichment Score: 0.4430177226352198**

**REFSEQ_MRNA Gene Name**

NM_183004, NM_001969 eukaryotic translation initiation factor 5

NM_021183 RAP2C, member of RAS oncogene family

NM_019067 guanine nucleotide binding protein-like 3 (nucleolar)-like

NM_014170, NM_138485 GTP-binding protein 8 (putative)

**Gene Group 8 Enrichment Score: 0.44263200247174056**

**REFSEQ_MRNA Gene Name**

NM_021183 RAP2C, member of RAS oncogene family

NM_016277, NM_183227 RAB23, member RAS oncogene family

NM_021033 RAP2A, member of RAS oncogene family

NM_002884, NM_001010935 RAP1A, member of RAS oncogene family

**Gene Group 9 Enrichment Score: 0.3860957880559414**

**REFSEQ_MRNA Gene Name**

NM_013381 thyrotropin-releasing hormone degrading enzyme

NM_023075 metallophosphoesterase 1

NM_032558 hippocampus abundant transcript-like 1

NM_024056 transmembrane protein 106C

NM_002182 interleukin 1 receptor accessory protein

NM_004028, NM_001650 aquaporin 4

NM_001007527 LMBR1 domain containing 2

NM_014607 UBX domain protein 4

NM_024769 adipocyte-specific adhesion molecule

NM_138727, NM_017744, NM_138728 suppression of tumorigenicity 7 like

NM_002099 glycophorin A (MNS blood group)

NM_181644 major facilitator superfamily domain containing 4

NM_019026 transmembrane and coiled-coil domains 1

NM_006691 lymphatic vessel endothelial hyaluronan receptor 1

NM_173570 zinc finger, DHHC-type containing 23

NM_152666 phospholipase D family, member 5

NM_022736 major facilitator superfamily domain containing 1

NM_139321 attractin

NM_016388 T cell receptor associated transmembrane adaptor 1

NM_014702 KIAA0408; chromosome 6 open reading frame 174

NM_007072 HERV-H LTR-associating 2

NM_153711 family with sequence similarity 26, member E

NM_080927 discoidin, CUB and LCCL domain containing 2

NM_001627 hypothetical protein LOC100133690; activated leukocyte cell adhesion molecule

NM_000950 proline rich Gla (G-carboxyglutamic acid) 1

NM_001080505 shisa homolog 3 (Xenopus laevis)

NM_032256 transmembrane protein 117

NM_138999 neuropilin (NRP) and tolloid (TLL)-like 1

NM_201613 IKK interacting protein

NM_024334 transmembrane protein 43

NM_007175 ER lipid raft associated 2

NM_001795 cadherin 5, type 2 (vascular endothelium)

NM_024569 myelin protein zero-like 1

NM_152391 PQ loop repeat containing 3

NM_001777, NM_198793, NM_001025079 CD47 molecule

NM_030791 sphingosine-1-phosphate phosphatase 1

NM_207015 N-acetylated alpha-linked acidic dipeptidase-like 2

NM_024795 transmembrane 4 L six family member 20

NM_006566 CD226 molecule

NM_138820 HIG1 hypoxia inducible domain family, member 2A

NM_001079802, NM_006731 fukutin

NM_134431 solute carrier organic anion transporter family, member 1A2

NM_033428 chromosome 9 open reading frame 123

NM_001012642 GRAM domain containing 2

NM_005810 killer cell lectin-like receptor subfamily G, member 1

NM_016127 transmembrane protein 66

NM_052885 solute carrier family 2 (facilitated glucose transporter), member 13

NM_212558 transmembrane protein 215

NM_000878 interleukin 2 receptor, beta

NM_152261 chromosome 12 open reading frame 23

**Gene Group 10 Enrichment Score: 0.3131374839087871**

**REFSEQ_MRNA Gene Name**

NM_004814 small nuclear ribonucleoprotein 40kDa (U5)

NM_001007466, NM_020245 tubby like protein 4

NM_007178 serine/threonine kinase receptor associated protein

NM_001008707, NM_004434 echinoderm microtubule associated protein like 1

NM_020779, NM_001006657 WD repeat domain 35

NM_015726 WD repeat domain 42A

**Gene Group 11 Enrichment Score: 0.26531613297760653**

**REFSEQ_MRNA Gene Name**

NM_014571 hairy/enhancer-of-split related with YRPW motif-like

NM_012257 HMG-box transcription factor 1

NM_018419 SRY (sex determining region Y)-box 18

NM_021951 doublesex and mab-3 related transcription factor 1

**Arresting gd20 Endometrium > Healthy gd20 Endometrium**

**Gene Functional Classification Result**

**Gene Group 1 Enrichment Score: 6.038251262143185**

**REFSEQ_MRNA Gene Name**

NM_000090 collagen, type III, alpha 1

NM_015719 collagen, type V, alpha 3

NM_001845 collagen, type IV, alpha 1

NM_001846 collagen, type IV, alpha 2

NM_000088 collagen, type I, alpha 1

NM_000393 collagen, type V, alpha 2

NM_001844 collagen, type II, alpha 1

NM_001858 collagen, type XIX, alpha 1

NM_000092 collagen, type IV, alpha 4

NM_001855 collagen, type XV, alpha 1

NM_001854 collagen, type XI, alpha 1

NM_000089 collagen, type I, alpha 2

**Gene Group 2 Enrichment Score: 5.565701600910276**

**REFSEQ_MRNA Gene Name**

NM_153607 chromosome 5 open reading frame 41

NM_022728 neurogenic differentiation 6

NM_022462 hypoxia inducible factor 3, alpha subunit

NM_032440 ligand dependent nuclear receptor corepressor

NM_005595 nuclear factor I/A

NM_003222 transcription factor AP-2 gamma (activating enhancer binding protein 2 gamma)

NM_053002 mediator complex subunit 12-like

NM_006874 E74-like factor 2 (ets domain transcription factor)

NM_021961 TEA domain family member 1 (SV40 transcriptional enhancer factor)

NM_018416 forkhead box J2

NM_021813 BTB and CNC homology 1, basic leucine zipper transcription factor 2

NM_003112 Sp4 transcription factor

NM_006164 nuclear factor (erythroid-derived 2)-like 2

**Gene Group 3 Enrichment Score: 4.354901871995706**

**REFSEQ_MRNA Gene Name**

NM_004379 cAMP responsive element binding protein 1

NM_018416 forkhead box J2

NM_012081 elongation factor, RNA polymerase II, 2

NM_032329 inhibitor of growth family, member 5

NM_001621 aryl hydrocarbon receptor

NM_021961 TEA domain family member 1 (SV40 transcriptional enhancer factor)

NM_005316 general transcription factor IIH, polypeptide 1, 62kDa

NM_003822 nuclear receptor subfamily 5, group A, member 2

NM_153620 homeobox A1

NM_014071 nuclear receptor coactivator 6

NM_018951 homeobox A10

NM_007162 transcription factor EB

NM_006164 nuclear factor (erythroid-derived 2)-like 2

NM_053002 mediator complex subunit 12-like

NM_004229 mediator complex subunit 14

NM_000192 T-box 5

NM_207291 upstream transcription factor 2, c-fos interacting

NM_003074 SWI/SNF related, matrix associated, actin dependent regulator of chromatin, subfamily c, member 1

NM_057175 NMDA receptor regulated 1

**Gene Group 4 Enrichment Score: 3.309175637887512**

**REFSEQ_MRNA Gene Name**

NM_005180 BMI1 polycomb ring finger oncogene

NM_152493 zinc finger protein 362

NM_032440 ligand dependent nuclear receptor corepressor

NM_032329 inhibitor of growth family, member 5

NM_022893 B-cell CLL/lymphoma 11A (zinc finger protein)

NM_022552 DNA (cytosine-5-)-methyltransferase 3 alpha

NM_017544 NFKB repressing factor

NM_003822 nuclear receptor subfamily 5, group A, member 2

NM_015457 zinc finger, DHHC-type containing 5

NM_003575 zinc finger protein 282

NM_004241 jumonji domain containing 1C

NM_003597 Kruppel-like factor 11

NM_003111 Sp3 transcription factor

NM_030625 tet oncogene 1

NM_013450 bromodomain adjacent to zinc finger domain, 2B

NM_015534 zinc finger, ZZ-type containing 3

NM_015481 zinc finger protein 385A

NM_022781 ring finger protein 38

NM_152787 mitogen-activated protein kinase kinase kinase 7 interacting protein 3

NM_006624 zinc finger, MYND domain containing 11

NM_003112 Sp4 transcription factor

NM_014491 forkhead box P2

NM_005234 nuclear receptor subfamily 2, group F, member 6

NM_012234 RING1 and YY1 binding protein

**Gene Group 5 Enrichment Score: 1.0335908746767928**

**REFSEQ_MRNA Gene Name**

NM_002314 LIM domain kinase 1

NM_152835 PDLIM1 interacting kinase 1 like

NM_005627 serum/glucocorticoid regulated kinase 1

NM_014397 NIMA (never in mitosis gene a)-related kinase 6

NM_006852 tousled-like kinase 2

NM_032430 BR serine/threonine kinase 1

NM_003010 mitogen-activated protein kinase kinase 4

NM_021643 tribbles homolog 2 (Drosophila)

NM_001222 calcium/calmodulin-dependent protein kinase II gamma

NM_198465 Nik related kinase

**Gene Group 6 Enrichment Score: 0.6173478677132618**

**REFSEQ_MRNA Gene Name**

NM_018906 protocadherin alpha 3

NM_012329 monocyte to macrophage differentiation-associated

NM_001795 cadherin 5, type 2 (vascular endothelium)

NM_016548 golgi membrane protein 1

NM_018898, NM_018901, NM_018899 protocadherin alpha 13; protocadherin alpha 10; protocadherin alpha subfamily C, 1; protocadherin alpha subfamily C, 2

NM_013281 fibronectin leucine rich transmembrane protein 3

NM_018903 protocadherin alpha 12

NM_018910 protocadherin alpha 7

NM_005329 hyaluronan synthase 3

NM_002998 syndecan 2

NM_018900 protocadherin alpha 1; protocadherin alpha 4

NM_018909, NM_018911 protocadherin alpha 8; protocadherin alpha 6

NM_005927 microfibrillar-associated protein 3

NM_018905 protocadherin alpha 2

NM_018976 solute carrier family 38, member 2

NM_005302 G protein-coupled receptor 37 (endothelin receptor type B-like)

NM_152527 solute carrier family 16, member 14 (monocarboxylic acid transporter 14)

**Gene Group 7 Enrichment Score: 0.3853981926861714**

**REFSEQ_MRNA Gene Name**

NM_017423 UDP-N-acetyl-alpha-D-galactosamine:polypeptide N-acetylgalactosaminyltransferase 7 (GalNAc-T7)

NM_005329 hyaluronan synthase 3

NM_016548 golgi membrane protein 1

NM_014918 chondroitin sulfate synthase 1

NM_152996 ST6 (alpha-N-acetyl-neuraminyl-2,3-beta-galactosyl-1,3)-N-acetylgalactosaminide alpha-2,6-sialyltransferase 3

NM_003779 UDP-Gal:betaGlcNAc beta 1,4- galactosyltransferase, polypeptide 3

**Gene Group 8 Enrichment Score: 0.03539527101621054**

**REFSEQ_MRNA Gene Name**

NM_153365 transmembrane anterior posterior transformation 1

NM_152400 chromosome 4 open reading frame 32

NM_138391 transmembrane protein 183A; transmembrane protein 183B

NM_015008 transmembrane and coiled-coil domain family 1

NM_173683 XK, Kell blood group complex subunit-related family, member 6

NM_016548 golgi membrane protein 1

NM_138390 transmembrane protein 169

NM_005329 hyaluronan synthase 3

NM_002998 syndecan 2

NM_005927 microfibrillar-associated protein 3

NM_014283 chromosome 1 open reading frame 9

NM_152527 solute carrier family 16, member 14 (monocarboxylic acid transporter 14)

NM_031434 transmembrane and ubiquitin-like domain containing 1

**Healthy gd20 Trophoblast > Arresting gd20 Trophoblast**

**Gene Functional Classification Result**

No differences in miRNAs observed.

**Arresting gd20 Trophoblast > Healthy gd20 Trophoblast**

**Gene Functional Classification Result**

No differences in miRNAs observed.
